# Non-operative treatment strategy versus surgery for children with simple appendicitis: non-inferiority randomised controlled trial

**DOI:** 10.1136/bmjmed-2025-002466

**Published:** 2026-05-13

**Authors:** Paul van Amstel, Said Bachiri, Max Knaapen, Johanna H van der Lee, Rik van Eekelen, Willem A Bemelman, Hester Rippen-Wagner, Taco S Bijlsma, Charlotte F J M Blanken-Peeters, Evert-Jan G Boerma, Anne Loes van den Boom, Frank J C van den Broek, Huib A Cense, Peter van Duijvendijk, Frank P Garssen, Klaas H in ’t Hof, Vanessa J Leijdekkers, Maarten A Lijkwan, Gerda W Zijp, Hugo A Heij, Joep P M Derikx, Ernest L W van Heurn, Roel Bakx, Ramon R Gorter, on behalf of the APAC collaborative study group, JH Allema

**Affiliations:** 1 Department of Paediatric Surgery, Amsterdam UMC Locatie AMC, Amsterdam, Netherlands; 2 Amsterdam Reproduction and Development Research Institute, Amsterdam, Netherlands; 3 University of Amsterdam, Amsterdam Gastroenterology Endocrinology Metabolism Research Institute, Amsterdam, Netherlands; 4 Kennisinstituut van de Federatie Medisch Specialisten, Utrecht, Netherlands; 5 Centre for Reproductive Medicine, University of Amsterdam, Amsterdam UMC, Amsterdam, Netherlands; 6 Department of Surgery, Amsterdam UMC-Locatie AMC, Amsterdam, Netherlands; 7 Stichting Kind & Zorg, Utrecht, Netherlands; 8 Department of Surgery, Noordwest Ziekenhuisgroep, Alkmaar, Netherlands; 9 Department of Surgery, Rijnstate, Arnhem, Netherlands; 10 Department of Surgery, Zuyderland, Heerlen/Sittard, Netherlands; 11 Department of Surgery, St Antonius Ziekenhuis, Nieuwegein, Netherlands; 12 Department of Surgery, Maxima Medisch Centrum, Veldhoven, Netherlands; 13 Department of Surgery, Rode Kruis Ziekenhuis, Beverwijk, Netherlands; 14 Department of Surgery, Gelre Hospital, Apeldoorn, Netherlands; 15 Department of Surgery, Amstelland Hospital, Amstelveen, Netherlands; 16 Department of Surgery, Flevo Hospital, Almere, Netherlands; 17 Department of Surgery, Onze Lieve Vrouwe Gasthuis, Amsterdam, Netherlands; 18 Department of Surgery, Albert Schweitzer Ziekenhuis, Dordrecht, Netherlands; 19 Department of Surgery, Juliana Children's Hospital, The Hague, Netherlands

**Keywords:** General surgery, Pediatrics, Emergency medicine, Gastroenterology

## Abstract

**Objective:**

To compare a non-operative treatment strategy with appendectomy for children with simple appendicitis, in terms of complications, avoidance of appendectomy, health related quality of life, and costs, after one year of follow-up.

**Design:**

Non-inferiority randomised controlled trial.

**Setting:**

15 academic or general teaching hospitals, Netherlands, 1 January 2017 to 19 October 2023.

**Participants:**

302 children, aged 7-17 years with imaging confirmed simple appendicitis, excluding those with a faecolith.

**Interventions:**

Participants were randomised to the non-operative treatment strategy group (n=151) or the appendectomy group (n=151). The non-operative treatment strategy group received 48 hours of intravenous amoxicillin-clavulanic acid and gentamicin, followed by five days of oral amoxicillin-clavulanic acid.

**Main outcome measures:**

The primary outcome was the proportion of participants with a complication within one year (non-inferiority margin of 5%). Secondary outcomes were the number of participants not requiring appendectomy, health related quality of life, and costs.

**Results:**

In the intention-to-treat population, 14/151 (9.3%) participants in the non-operative treatment strategy group versus 13/151 (8.6%) in the appendectomy group had a complication during the one year follow-up period (absolute risk difference 0.7%, upper limit of 95% confidence interval (CI) 7.1%). Appendectomy was avoided in 105/151 (69.5%) participants in the non-operative treatment strategy group. No difference was found in health related quality of life between the treatment groups. Costs (both direct and societal) were lower in the non-operative treatment strategy group (mean difference €767 (£668; US$902), 95% CI 320 to 1214 for direct costs and €874, 118 to 1631 for societal costs). In the non-operative treatment strategy group, length of hospital stay was significantly longer (3.3±2.4 *v* 1.9±1.2 days, mean difference 1.5, 95% CI 1.1 to 1.9) and more unscheduled healthcare visits occurred. Patient satisfaction measured by the Net Promoter Score was significantly lower in the non-operative treatment strategy group (18.9±4.6% *v* 48.0±5.8%, mean difference −29.1, 95% CI−30.8 to −27.4) at the one year follow-up.

**Conclusions:**

Non-inferiority of a non-operative treatment strategy for acute simple appendicitis in children could not be proven significantly. Most of the 95% CI was within the 5% non-inferiority margin, however, with a probability of 90.7% that a non-operative treatment strategy resulted in an increase in complications of ≤5%. Absolute risk difference in the proportion of participants with complications was marginal. Surgery was avoided in about 70% of participants in the non-operative treatment strategy group. Health related quality of life was comparable between the treatment strategies. A non-operative treatment strategy was associated with lower costs, but longer time in hospital, more unscheduled healthcare visits, and lower patient satisfaction at the one year follow-up, measured by the Net Promoter Score.

**Trial registration:**

NCT02848820; NTR5977.

**WHAT IS ALREADY KNOWN ON THIS TOPIC**

- The feasibility of a non-operative treatment strategy in children with simple appendicitis has been established in several cohort studies
- High quality evidence (ie, randomised trials comparing appendectomy with a non-operative treatment strategy) was not available until recently

**WHAT THIS STUDY ADDS**

- A non-operative treatment strategy was not non-inferior to appendectomy in terms of complications, but absolute differences in complications were marginal
- Advantages of a non-operative treatment strategy were the ability to prevent appendectomy in about 70% of children with simple appendicitis and lower costs at the one year follow-up
- Disadvantages of a non-operative treatment strategy were longer hospital admission and more unscheduled healthcare visits

**HOW THIS STUDY MIGHT AFFECT RESEARCH, PRACTICE, OR POLICY**

- The results of the APAC trial contribute to improved knowledge about the advantages and disadvantages of a non-operative treatment strategy in children with simple appendicitis
- The findings will enable children, their parents or caregivers, and physicians to engage in evidence based shared decision making when choosing between appendectomy and a non-operative treatment strategy as a treatment option

## Introduction

 Interest in the comparative outcomes between a non-operative treatment strategy and appendectomy in children with acute simple appendicitis in clinical practice is growing. This paradigm shift is strongly guided by the ethical and clinical principle of “primum non nocere” or “first, do no harm,” which emphasises minimising patient risk and avoiding unnecessary invasive procedures whenever possible, reserving surgical intervention for when unequivocally necessary, particularly in paediatric patients.^1^ Although surgical intervention provides immediate resolution of the disease, direct intraoperative assessment and treatment for alternative diagnoses during laparoscopy is also possible. Surgical intervention carries inherent short term postoperative risks, however, such as infection, haemorrhage, anaesthesia related complications, longer recovery times, pain, and temporary functional impairment.^2 3^ Non-operative treatment, in contrast, offers a less invasive alternative, avoiding immediate and long term harms associated with surgery. From a healthcare perspective, a non-operative treatment strategy can reduce associated costs without compromising safety in appropriately selected patients with simple appendicitis.^4^ These arguments support a treatment paradigm by avoiding unnecessary surgical harm while still effectively managing the disease. The potential disadvantages of a non-operative treatment strategy are the rate of treatment failures and recurrent appendicitis, antimicrobial resistance, and the related adverse effects of antibiotics.^5^,^7^ Also, the potential delay or masking of a non-operative treatment strategy in the event of unacknowledged complicated appendicitis or alternative diagnoses may result in potentially worse outcomes than initial surgical treatment.^8^

Several studies have explored this non-operative treatment strategy (consisting of antibiotics given intravenously, orally, or both) for acute simple appendicitis in adult and paediatric populations.^9^,^11^ In the adult population, a recent individual patient data meta-analysis, with data from six randomised controlled trials in a total of 2101 patients, showed that a non-operative treatment strategy was a safe alternative to appendectomy with comparable complication rates (non-operative treatment 5.4% *v* operative treatment 8.3%). Moreover, in two thirds of patients treated non-operatively, appendectomy could be avoided after one year of follow-up.^12^

In the paediatric population, data for the outcome of a non-operative treatment strategy for simple appendicitis are limited to cohort studies, patient preference studies, and small pilot randomised controlled trials.^7 10 11^ Recently, two large randomised controlled trials have been published in the paediatric population, concluding that non-operative treatment was inferior to appendectomy in terms of treatment failure. Nevertheless, surgery was avoided in about two thirds of children in the randomised controlled trial by St Peter et al.^13 14^

To contribute to the ongoing discussion about the role of non-operative treatment for simple appendicitis in the paediatric population, we conducted a multicentre, non-inferiority randomised controlled trial (Initial Non-operative Treatment Strategy Versus Appendectomy Treatment Strategy for Simple Appendicitis in Children (APAC) trial) in the Netherlands. When this trial was designed, small pilot data suggested that a non-operative treatment strategy would result in a reduction in complications (superiority), but the evidence was weak. Because of the lack of data to support the superiority of a non-operative treatment strategy at that time, a non-inferiority design was chosen because if equivalence could be shown in terms of complications, non-operative treatment would have other beneficial secondary outcomes to appendectomy. The aim of this study was to compare the outcomes of a non-operative treatment strategy and appendectomy for children aged 7-17 years with radiologically confirmed acute simple appendicitis, in terms of complications, avoidance of surgery after non-operative treatment, health related quality of life and costs.

## Methods

### Study design

We conducted an unblinded, multicentre, parallel, randomised controlled non-inferiority trial (adhering to the CONSORT (Consolidated Standards of Reporting Trials) guideline) with 1:1 block randomisation grouped by hospital. The study was conducted in 15 hospitals (both academic and general teaching hospitals) in the Netherlands. The protocol was developed in adherence with the SPIRIT (Standard Protocol Items: Recommendations for Interventional Trials) guideline and has been published previously (online supplemental file 1).^15 16^ The study was registered at ClinicalTrials.gov (NCT02848820) and the Dutch Trial Registry (NTR5977) before enrolment.

### Participants

All children aged 7-17 years with an imaging confirmed diagnosis of acute simple appendicitis were eligible for inclusion. The minimum age of seven years was chosen for safety reasons because children aged <7 years have an increased risk of complex appendicitis and are less able to accurately verbalise their symptoms.^17^ Simple appendicitis was defined according to clinical and radiological criteria: localised tenderness in the right iliac fossa, normal or hyperactive bowel sounds, no guarding or palpable mass, raised white blood cell count, raised C reactive protein levels, a non-compressible and painful appendix with an outer diameter of >6 mm on ultrasound, secondary signs of inflammation (ie, infiltration of the surrounding fat) on ultrasound, and hyperaemia of the appendiceal wall on ultrasound.^18^ If ultrasound was inconclusive, additional imaging (ie, magnetic resonance imaging or computed tomography scan) was performed. All participants were required to have an imaging confirmed diagnosis of uncomplicated appendicitis, but did not necessarily have to show all of the previously mentioned symptoms.

Excluded from participation in the study were patients with generalised peritonitis or sepsis (defined according to the international paediatric sepsis conference),^19^ imaging findings indicative of complex appendicitis (ie, substantial amount of free fluid, perforation of the appendix, signs of an intra-abdominal abscess, or phlegmon), presence of a faecolith on imaging (defined as an echogenic, well defined focus within the appendix with posterior acoustic shadowing), serious comorbidities, such as cardiac or pulmonary disease with major haemodynamic consequences, severe immunodeficiency, malignancy, or sickle cell disease, patients that were treated non-operatively for acute appendicitis previously, suspicion of underlying malignancy or inflammatory bowel disease, recorded type 1 allergy to the antibiotics used in the study protocol, or a complex appendicitis risk score of ≥4. The complex appendicitis risk score is based on five clinical and biochemical variables (ie, abdominal guarding, signs of complex appendicitis on ultrasound, C reactive protein level, temperature, and days of abdominal pain) that are assigned a certain number of points. In a previous study, patients with a score of <4 points had a negative predictive value for complex appendicitis of 98% (95% confidence interval (CI) 88% to 100%).^20^

### Randomisation

After obtaining written informed consent, participants were randomised to the non-operative treatment strategy group or the appendectomy group with the web based randomisation programme, Castor Electronic Data Capture version 4.10 (Castor). For concealment of allocation, a 1:1 variable block randomisation was used, grouped by hospital. Masking of participants, the treating surgeon, or (local) investigators conducting follow-up of patients was not feasible. Data analysis was conducted unblinded because the analyst had access to the treatment allocation throughout the analytical process.

### Study setting and procedures

**Figure 1:**
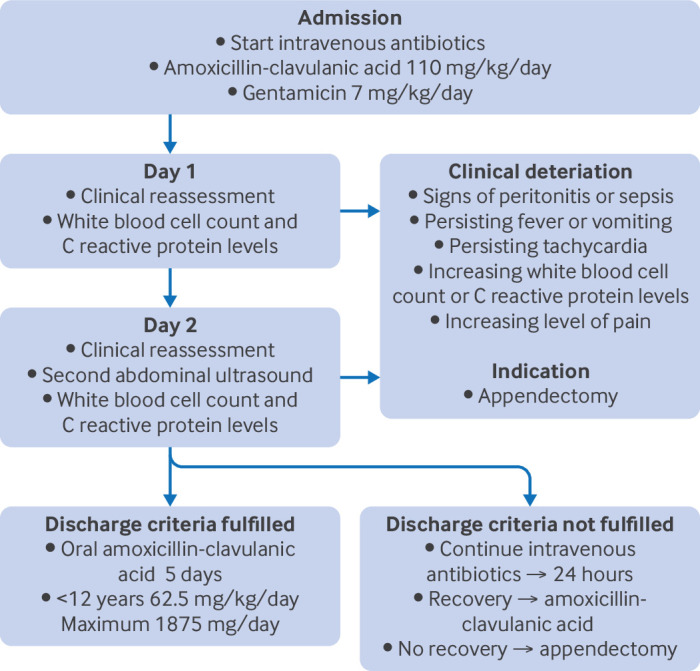
Flowchart of non-operative treatment strategy. Online supplemental table S1 shows the predefined discharge criteria

Participants randomly assigned to the non-operative treatment strategy group were treated with intravenous amoxicillin-clavulanic acid (25/2.5 mg/kg every six hours, maximum dose 6000/600 mg/day, respectively) and gentamicin (7 mg/kg once daily) for a total of 48 hours. If predefined discharge criteria were met (including a follow-up ultrasound with no signs of complex appendicitis), participants were discharged with oral antibiotics (amoxicillin-clavulanic acid 50/12.5 mg/kg in three daily doses). If discharge criteria were not met after 48 hours, intravenous antibiotics were continued to a maximum total duration of 72 hours. The total duration of antibiotics (combination of intravenous and oral use) was seven days. During hospital admission, participants were assessed daily, and infection parameters were measured at 24 and 48 hours to detect clinical deterioration at an early stage. If predefined signs of clinical deterioration were met, the treating physician could decide to proceed with urgent appendectomy (figure 1). In instances of recurrent appendicitis during follow-up, participants underwent an appendectomy.

Participants randomly assigned to the appendectomy group received intravenous fluids and drug treatment for pain according to the same protocol as the non-operative treatment strategy group. Appendectomy was performed according to the Dutch national guideline.^21^ The surgical approach (open or laparoscopy) was at the discretion of the treating surgeon. Antibiotic prophylaxis and postoperative care were given according to local protocols. In the event of unexpected findings of complicated appendicitis during operation, participants were treated with postoperative antibiotics according to local protocols. Participants were discharged when the same predefined discharge criteria were met as described in the non-operative treatment strategy group (online supplemental table S1).

All participants or their parents were contacted by telephone by the study coordinators at seven days, six months, and one year after randomisation. The treating physician scheduled an outpatient visit after four weeks. Questionnaires were sent at these follow-up times to measure health related quality of life, patient satisfaction, and costs. To optimise the response rate to the questionnaire, automated reminders were scheduled at each follow-up time point and were sent two weeks after the first dispatch of the questionnaire.

### Study outcomes

The primary outcome of the study was the proportion of participants who had a complication during the one year follow-up period. A detailed list of potential complications has been reported in the previously published study protocol.^16^ Complications were defined as, but not limited to: complications of antibiotic use (eg, allergic reactions or gastrointestinal distress); requirement of surgical or radiological intervention other than appendectomy (but still related to appendicitis); readmission for an indication related to the allocated treatment, apart from recurrent appendicitis (reported as a separate outcome); and complications associated with appendectomy, such as surgical site infections, stump leakage or stump appendicitis, secondary bowel obstruction, anaesthesia related complications, and incisional hernia.

An independent adjudication committee reviewed all reported complications to assess their relation to the allocated treatment, and categorised the severity of the complications according to the Clavien-Dindo and Clavien-Madadi systems.^22 23^ Minor complications were defined as Clavien-Dindo or Clavien-Madadi grade 1 and 2 complications, and major complications as Clavien-Dindo or Clavien-Madadi grade 3 or higher. The adjudication committee comprised four independent reviewers who each assessed all potential complications separately and without knowledge of the initial treatment allocation. Consensus was defined as agreement among at least three of the four reviewers. To reach consensus, three written, blinded review rounds were conducted. If consensus was not reached during these rounds, the event was then discussed in a closed meeting of the committee members, after which a final consensus was established.

Any form of delayed appendectomy, including early failure during admission and recurrent appendicitis during follow-up, was not considered a complication because the non-operative treatment strategy was considered a step-up approach in which appendectomy was warranted for all participants if antibiotic treatment did not resolve the symptoms. Furthermore, for fair comparison between treatment strategies, early failure and recurrent appendicitis were considered important outcomes, and therefore we displayed these outcomes separately rather than as part of a composite outcome for independent assessment and interpretation. Complications resulting from a delayed appendectomy were included in the primary outcome. The rate of delayed appendectomy was reported as a secondary outcome, including early failure during initial admission, recurrent appendicitis, and interval appendectomy with no signs of appendicitis on histopathological examination. The percentage of participants in whom appendectomy could be avoided was also reported as a secondary outcome. Types of complications were also reported as secondary outcomes. Although this trial had already commenced before the development of the core outcome set for appendicitis in children, the trial aligns with the outcome reporting, as agreed by a large group of experts in 2022.^24^ Other secondary outcomes were categorised as listed below. All endpoints were evaluated at the one year follow-up unless stated otherwise.

### Appendectomy related endpoints

Appendectomy related endpoints were: percentage of participants with a missed diagnosis of complex appendicitis; percentage of participants undergoing appendectomy during the first antibiotic course (ie, early failure of non-operative treatment); participants with recurrent appendicitis (histopathologically confirmed); percentage of participants undergoing interval appendectomy on parental request (histopathologically no sign of recurrent appendicitis); and percentage of participants with a negative appendectomy.

### Participant related endpoints

Level of pain was assessed with the Numeric Rating Scale and total use of drug treatments for pain on day 7. Health related quality of life was assessed with the validated Child Health Questionnaire-Child Form 87 (version 2013),^25^ the validated European Quality of Life-5 Dimensions-Youth questionnaire (child perspective) (version 6.0), and the validated European Quality of Life-5 Dimensions-Proxy questionnaire (parent perspective)^26^ (version 6.0) at baseline, and at follow-up at seven days, one month, six months, and one year.

Patient satisfaction was assessed with the Net Promoter Score after seven days, one month, six months, and one year, and the validated Patient Satisfaction Questionnaire-18 after seven days, one month, and one year.^27^ Number of days absent from school, or social or sport events (participant level) was calculated, as well as the number of days absent from work (parent level). Total number of extra visits to the outpatient clinic, general practitioner's office, or emergency department for abdominal pain was recorded and total length of hospital stay (days) during the follow-up period, including admissions for complications related to the allocated treatment. The length of initial hospital stay was included and reported separately.

### Cost related endpoints

The cost endpoints differed slightly from those published in our study protocol because of updates in the cost manual of the Dutch Healthcare Institute that was used to define the cost endpoints in this trial.^28^ For medical costs or actual healthcare costs, variables included were, but were not limited to, the number of follow-up outpatient clinic visits, number of visits to a general practitioner, number of emergency department visits, and actual in-hospital generated costs. Non-medical costs included transfer costs (eg, transfers by public transportation, ambulance costs, and vehicle parking costs), costs of informal caregivers, and other costs not directly related to the treatment of appendicitis. To calculate these costs, we used the Medical Consumption Questionnaire^29^ (version February 2013) and the Productivity Cost Questionnaire^30^ (version February 2013), adapted for use in children and parents. These questionnaires were dispatched at the one month, six month, and one year follow-up times. Societal costs consisted of a combination of direct medical and non-medical costs and indirect costs associated with loss of productivity.

### Statistical analyses

The sample size calculation has been described in our previously published study protocol.^16^ At the time this trial was designed, small pilot data suggested that the non-operative treatment strategy could reduce the complication rate by 50% compared with appendectomy. As the evidence was still weak for superiority, we decided to conduct a non-inferiority trial in which at least non-operative treatment was non-inferior to appendectomy, but could lead to substantially more favourable secondary outcomes in terms of less disability days, better quality of life, and lower costs than appendectomy. Based on our previous pilot study, the overall complication rate after appendectomy for children with simple appendicitis was set at 10%.^31^ We powered on a non-inferiority margin of 5%, which means that we were willing to, at most, accept a 5% increase in complication rate (to 15%) in the non-operative treatment strategy group compared with the appendectomy group, given the other advantages of the non-operative treatment strategy.

We also decided on what we assumed to be the true difference in complication rate for the non-inferiority design. Based on the literature available at the time of the sample size calculation, we assumed that avoiding surgery with the non-operative treatment strategy would reduce the complication rate by 50%, resulting in a true complication rate of 5% in the non-operative treatment strategy group.^32^,^35^ With a one sided α value of 2.5% (according to non-inferiority principles, such that the value coincides with the standard 95% CI), we needed 150 participants in each treatment group to achieve 90% power to exclude a difference in favour of the appendectomy group of ≥5%. We assumed a dropout rate of 10%, resulting in a sample size of 334 participants. After inclusion of >90% of participants, however, only two participants were lost to follow-up and therefore the sample size was reduced to 302 participants. No interim analysis was performed.

The primary analysis of the outcomes was done according to the intention-to-treat principle (by author RvE). Only participants who changed from the randomly assigned treatment to the other treatment group based on participant or parent preference without medical grounds were considered treatment arm crossover participants. Therefore, participants who received an appendectomy for early failure or recurrence of disease were not considered as a crossover. Because the intention-to-treat analysis can underestimate the treatment effect in non-inferiority trials, leading to an inappropriate rejection of the null hypothesis, a secondary per protocol analysis and an as-treated analysis were also performed.^36^ In the intention-to-treat analysis, the entire study population was evaluated according to the initial randomisation of the treatment strategy, regardless of any crossover that may have occurred. In the per protocol analysis, only participants who strictly adhered to the assigned treatment according to the randomisation or study protocol and who completed the full study follow-up were included. Finally, the as-treated analysis assessed outcomes based on the actual treatment received by participants, irrespective of their original randomisation assignment.

The primary outcome was calculated based on risk differences with a one sided 97.5% CI upper limit, in accordance with the non-inferiority design. Absolute differences in proportions are shown for the primary outcome. We used confidence distributions (based on the CI of the difference in proportions between groups) to state the estimated probability that the non-operative treatment strategy resulted in an increase in complications of ≤5%.^37^ Finally, as a sensitivity analysis, we included centre as a clustering variable for robust standard errors with a binomial model, with identity link function and a sandwich variance estimator, with the cluster term being centre, given that other approaches, such as random intercepts for centre, were not feasible because of the rarity of the primary outcome complications.

Secondary outcomes were visually tested for normal distribution with histograms and box plots. Normally distributed continuous secondary outcomes were analysed with unpaired t tests, and skewed distributed outcomes were analysed with the Mann-Whitney U test. Dichotomous secondary outcomes were analysed with χ^2^ tests. Outcomes are presented as differences in proportions and as absolute mean differences for continuous outcomes, each accompanied by corresponding 95% CIs. For all outcomes, the percentage of missing data was calculated and reported. No imputation of missing data was performed (online supplemental file 2).

All statistical analyses were performed with IBM SPSS Statistics, version 28.0 (IBM, Armonk, NY) and R version 4.4.2 and R Studio (R Core Team (2023)); R: A Language and Environment for Statistical Computing and R Foundation for Statistical Computing, Vienna, Austria (https://www.R-project.org).

### Patient and public involvement

The design and conduct of the trial was co-created in collaboration with a patient representative (HR-W) from the foundation Stichting Kind & Zorg and they will be involved in dissemination of the results to the public through their communication lines. HR-W is a coauthor of this manuscript.

## Results

**Figure 2:**
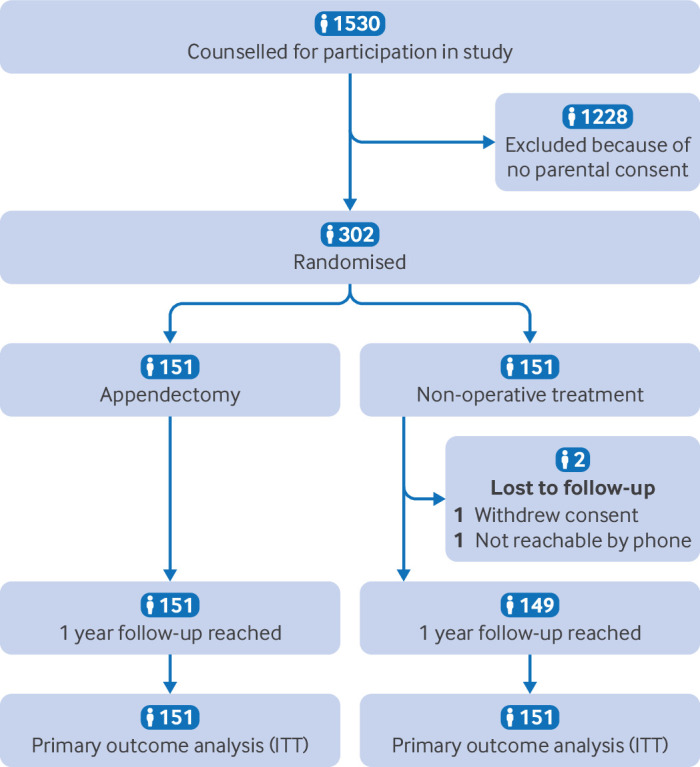
Flowchart of participants. Two patients in the non-operative treatment strategy group had previously had a known complication before being lost to follow-up and could therefore be included in the primary outcome analysis: proportion of patients with a complication at the one year follow-up. ITT=intention-to-treat population

Between 1 January 2017 and 31 October 2023, 1530 patients were eligible for inclusion and counselled for participation in the study. Of these, 302 (19.7%) patients were included from 15 hospitals in the Netherlands. Participants were evenly distributed; 151 participants in the appendectomy group (all underwent laparoscopic appendectomy) and 151 participants in the non-operative treatment strategy group (figure 2). One participant in the non-operative treatment strategy group withdrew consent 13 days after treatment and was no longer willing to complete the study. Consent was given to use the data already collected. The second loss to follow-up, also from the non-operative treatment strategy group, could not be reached from the one month follow-up period onwards. Table 1 shows the baseline clinical characteristics of the study population.

**Table 1:** Baseline characteristics of the study population

Characteristics	Appendectomy group (n=151)	Non-operative treatment strategy group (n=151)
Median (IQR) age (years)	13.1 (10.5-15.3)	12.0 (9.9-14.5)
Male sex	85 (56.3)	85 (56.3)
Median (IQR) height (cm)	159.0 (142.0-172.0)	158.0 (145.0-170.0)
Median (IQR) weight (kg)	44.5 (32.0-56.0)	45.0 (33.7-55.5)
Median (IQR) body mass index	18.4 (16.1-20.8)	18.6 (16.2-21.8)
Allergies to drug treatments	1 (0.7)	0 (0.0)
Past relevant illnesses	12 (7.9)	13 (8.6)
Median (IQR) No of days of abdominal pain	1.0 (1.0-2.0)	1.0 (1.0-1.5)
Fever (>38.5 °C)	3 (2.0)	5 (3.3)
Anorexia	66 (43.7)	62 (41.1)
Nausea	92 (60.9)	94 (62.3)
Vomiting	54 (35.8)	60 (39.7)
General appearance ill	3 (2.0)	5 (3.3)
Abdomen painful during palpation	146 (96.7)	148 (98.0)
Location of abdominal pain (right lower quadrant)	143 (94.7)	142 (94.0)
Median (IQR) pulse (beats/min)	87.5 (77.8-100.0)	90.0 (80.0-102.0)
Median (IQR) temperature (°C)	37.0 (36.6-37.4)	37.2 (36.7-37.7)
Median (IQR) leucocytes (10^9^/L)	13.0 (9.0-15.7)	13.1 (9.9-16.0)
Median (IQR) C reactive protein (mg/l)	18.0 (4.7-38.0)	19.0 (7.0-40.0)
Median (IQR) Numeric Rating Scale pain score	5.0 (3.0-6.0)	4.0 (3.0-7.0)

Data are number (%) unless indicated otherwise.

IQR, interquartile range.

### Primary outcome: proportion of participants with complications

In the appendectomy group, 13/151 (8.6%) participants had a complication within one year versus 14/151 (9.3%) participants in the non-operative treatment strategy group, resulting in an absolute risk difference of 0.7% (95% CI upper limit 7.1%). Because the upper limit of the 95% CI was greater than the predefined non-inferiority margin of 5%, the null hypothesis of the non-operative treatment strategy being inferior to appendectomy could not be rejected (P value for non-inferiority=0.09). Based on the (normal) sampling distribution, the probability that the non-operative treatment strategy resulted in an increase in complications of ≤5% was 90.7%. The analysis including centre as a clustering variable for robust standard errors showed similar results and inference. The per protocol and as-treated analyses showed similar results (table 2). The point estimate for the risk difference was the same (0.7%) and the 95% CI was −6.1% to 7.4%. Thus the upper limit increased to 7.4% (instead of 7.1%, as reported in our main analysis). The total number of complications was similar in the groups (14 complications in both groups (one patient had two complications during follow-up); absolute difference 0.0%, 95% CI −6.5% to 6.5%). No relevant differences were found in the severity of complications. We found minor complications in 10/151 (6.6%) patients in the non-operative treatment strategy group versus 8/151 (5.3%) in the appendectomy group. In the non-operative treatment strategy group, 4/151 (2.6%) participants had a major complication versus 5/151 (3.3%) in the appendectomy group (table 2).

**Table 2:** Primary outcome measure for the intention-to-treat, per protocol, and as-treated populations (non-inferiority margin of 5%) in the appendectomy and non-operative treatment strategy groups

Primary outcome measure	Appendectomy group (n=151)		Non-operative treatment strategy group *(*n=151)		Risk difference (%) (95% CI upper limit)	P value (for non-inferiority)
	No (%) of patients	Missing data (No (%))	No (%) of patients	Missing data (No (%))		
Intention-to-treat population						
Participants with complications at 1 year follow-up	13 (8.6)	—	14 (9.3)	—	0.7 (7.1)	0.09
Severity of complications						
Clavien-Dindo I	4 (2.6)	—	3 (2.0)	—	—	—
Clavien-Dindo II	4 (2.6)	—	7 (4.6)	—	—	—
Clavien-Dindo III	5 (3.3)	—	4 (2.6)	—	—	—
Clavien-Dindo IV	0 (0.0)	—	0 (0.0)	—	—	—
Per protocol population						
Participants with complications at 1 year follow-up*	11 (7.7)	—	14 (9.5)	—	1.8 (8.1)	0.15
As-treated population						
Participants with complications at 1 year follow-up†	11 (7.7)	—	16 (9.3)	2 (1.3)	2.4 (8.8)	0.21

Clavien-Dindo and Clavien-Madadi classifications were similar in this population.

* n=142 in the appendectomy group and n=148 in the non-operative treatment strategy group.

† n=143 in the appendectomy group and n=159 in the non-operative treatment strategy group.

CI, confidence interval.

The most common complications in the appendectomy group were intra-abdominal abscesses (n=3) and surgical site infections (n=3). Mild allergic reactions to the antibiotics were most commonly reported in the non-operative treatment strategy group (n=3), followed by intra-abdominal abscesses (n=2) and prolonged intravenous antibiotic course (n=2). Table 3 shows a complete overview of the types of complications in the two groups.

**Table 3:** Types of complications for the intention-to-treat population in the appendectomy and non-operative treatment strategy groups

Appendectomy group (n=14)*	Non-operative treatment strategy group (n=14)
Intra-abdominal abscess (n=3)	Intra-abdominal abscess (n=2)
Anterior cutaneous nerve entrapment syndrome (n=1)	Anterior cutaneous nerve entrapment syndrome (n=1)
Persistent abdominal pain symptoms (n=2)	Persistent abdominal pain symptoms (n=1)
Rash or mild allergic reaction to antibiotics (n=1)	Rash or mild allergic reaction to antibiotics (n=3)
Surgical site infection (n=3)	Constipation (n=1)
Panic attack during emergence from anaesthesia, requiring re-intubation (n=1)	Subcutaneous infiltration of the intravenous system (n=1)
Overdose of antibiotics causing nausea and diarrhoea (n=1)	Torsion of adnexa caused by error in diagnosis of appendicitis (n=1)
Stump appendicitis requiring surgical reintervention (n=1)	Bacteraemia after recurrent appendicitis (n=1)
Aspiration pneumonia (n=1)	Urinary tract infection (n=1)
	Extended admission for intravenous antibiotics (n=2)

* One participant in the appendectomy group had two complications (ie, stump appendicitis requiring surgical reintervention and intra-abdominal abscess).

### Secondary outcomes: appendectomy related endpoints

In 105/151 (69.5%) participants in the non-operative treatment strategy group, an appendectomy could be avoided at the one year follow-up. Indications for appendectomy during the one year follow-up period were early failure of non-operative treatment in 16/151 (10.6%) participants, recurrent appendicitis in 28/151 (18.5%) participants, and interval appendectomy caused by persistent abdominal complaints in 2/151 (2.3%) participants (table 4).

**Table 4:** Appendectomy related endpoints in the non-operative treatment strategy group

Appendectomy related endpoints	Non-operative treatment strategy group (n=151)
No appendectomy at 1 year follow-up	105 (69.5)
Recurrent appendicitis at 1 year follow-up	28 (18.5)
Early failure of initial non-operative treatment (during first 7 days)	16 (10.6)
Interval appendectomy at 1 year follow-up	2 (1.3)

Data are number (%).

We found no significant difference between the two groups for the proportion of participants with a missed diagnosis of complex appendicitis (absolute difference 2.7%, 95% CI −2.7% to 8.0%) (table 5). A negative appendectomy occurred in 4/151 (2.6%) participants in the appendectomy group whereas no negative appendectomies occurred in the non-operative treatment strategy group.

**Table 5:** Appendectomy and participant related endpoints in the appendectomy and non-operative treatment strategy groups

Secondary outcomes	Appendectomy group (n=151)		Non-operative treatment strategy group (n=151)		Absolute difference (%) or mean difference (95% CI)	P value (superiority or inferiority)
	No (%) or mean±SD	Missing data (No (%))	No (%) or mean±SD	Missing data (No (%)		
Total No of complications at 1 year follow-up	14	—	14	1 (0.7)	0.0 (−6.5 to 6.5)	>1.00
No (%) of participants with complications						
At discharge	3 (2.0)	—	7 (4.6)	—	2.7 (−1.4 to 6.7)	0.20
At 7 day follow-up	8 (5.3)	—	10 (6.6)	—	1.3 (−4.0 to 6.7)	0.63
At 1 month follow-up	12 (7.9)	—	10 (6.6)	—	−1.3 (−7.2 to 4.5)	0.66
At 6 months follow-up	13 (8.6)	—	14 (9.3)	—	0.7 (−5.8 to 7.1)	0.84
No (%) of participants with missed diagnosis of complex appendicitis	7 (4.6)	—	11 (7.3)	—	2.7 (−2.7 to 8.0)	0.33
Mean±SD initial length of hospital stay (days)	1.7±0.9	—	2.9±1.5	1 (0.7)	1.2 (0.9 to 1.5)*	0.001
Mean±SD total length of hospital stay (days)	1.9±1.2	1 (0.7)	3.3±2.4	1 (0.7)	1.5 (1.1 to 1.9)*	<0.001
Mean±SD level of pain in the first 7 days (VAS score)	1.3±1.6	—	1.0±1.8	2 (1.3)	0.3 (0.0 to 0.7)*	0.004
Mean±SD paracetamol use in the first 7 days (days)	4.2±1.9	—	3.5±2.4	2 (1.3)	0.7 (0.5 to 0.8)*	0.001
Mean±SD NSAID use in the first 7 days (days)	2.1±2.0	1 (0.7)	1.5±2.0	1 (0.7)	0.6 (0.5 to 0.8)*	<0.001
Mean±SD morphine use in the first 7 days (days)	0.2±0.7	—	0.1±0.4	1 (0.7)	0.1 (0.0 to 0.2)*	0.41
Mean±SD No of days absent from school, social, or sport events at 1 year follow-up	9.3±6.5	48 (31.8)	10.5±7.5	37 (24.5)	1.2 (0.7 to 3.1)*	0.29
Mean±SD No of days absent from work at 1 year follow-up	3.0±3.6	52 (34.4)	3.7±4.2	40 (26.5)	0.6 (0.4 to 1.7)*	0.22
Mean±SD total No of extra visits at 1 year follow-up	0.1±0.2	1 (0.7)	0.3±0.8	2 (1.3)	0.3 (0.1 to 0.4)*	<0.001

* Data are mean difference (95% CI).

CI, confidence interval; NSAID, non-steroidal anti-inflammatory drug; SD, standard deviation; VAS, visual analogue scale.

### Secondary outcomes: participant related endpoints

The level of pain during the first seven days of treatment was lower for the non-operative treatment strategy group (mean difference in Numeric Rating Scale 0.3, 95% CI 0.0 to 0.7). This finding was also reflected in significantly fewer days of paracetamol and non-steroidal anti-inflammatory drug use in the non-operative treatment strategy group during the first seven days of treatment (mean difference 0.7 days, 95% CI 0.5 to 0.8 for paracetamol use and mean difference 0.6 days, 0.5 to 0.8 for use of non-steroidal anti-inflammatory drugs) (table 5).

Health related quality of life reported by both participants and caregivers, as measured by the European Quality of Life-5 Dimensions questionnaire (EQ-5D), was significantly better in the non-operative treatment strategy group at seven days than in the appendectomy group (mean difference 0.04, 95% CI 0.004 to 0.08 and mean difference 0.12, 0.06 to 0.18, respectively) (table 6). We found no significant differences at the one month, six month, and one year follow-up times from the child's perspective. Health related quality of life scores reported by parents or caregivers were higher in the appendectomy group at the one year follow-up (mean difference −0.04, 95% CI −0.08 to 0.00). We found no significant differences at the one month or six month follow-up (table 6). For the Child Health Questionnaire-87 Domains questionnaire, we found no significant differences between the two treatment strategy groups at any of the follow-up times (online supplemental table S2).

**Table 6:** Health related quality of life scores in the appendectomy and non-operative treatment strategy groups

Questionnaire	Appendectomy group (n=151)		Non-operative treatment strategy group (n=151)		Mean difference (95% CI)	P value (non-parametric test)
	Score (mean±SD)	Missing data (No (%))	Score (mean±SD)	Missing data (No (%))		
EQ-5D-Y-3L (scale −0.329 to 1.000)						
Baseline	0.56±0.30	69 (45.7)	0.59±0.29	70 (46.4)	0.03 (−0.07 to 0.11)	0.616
7 days	0.86±0.12	45 (29.8)	0.90±0.16	58 (38.4)	0.04(0.004 to 0.08)	<0.001
1 month	0.96±0.08	65 (43.0)	0.95±0.13	60 (39.7)	−0.01 (−0.04 to 0.03)	0.640
6 months	0.99±0.04	78 (51.7)	0.97±0.08	82 (54.3)	−0.02 (−0.04 to 0.00)	0.053
1 year	0.98±0.05	80 (53.0)	0.96±0.09	75 (49.7)	−0.02 (−0.05 to 0.00)	0.113
EQ-5D-Y-3L visual analogue scale (scale 0 to 100)						
Baseline	54.2±24.0	91 (60.3)	55.0±21.2	67 (44.4)	0.8 (−6.8 to 8.4)	0.799
7 days	77.2±19.1	114 (75.5)	80.8±18.1	86 (57.0)	3.6 (−4.1 to 11.3)	0.104
1 month	88.8±9.8	65 (37.1)	87.0±12.9	60 (39.7)	−1.8 (−5.2 to 1.6)	0.591
6 months	91.0±11.8	75 (49.7)	89.1±11.8	83 (55.0)	−1.9 (−5.8 to 2.00)	0.753
1 year	91.1±9.3	79 (64.2)	92.2±9.3	76 (50.3)	1.1 (−1.9 to 4.1)	0.562
EQ-5D-3L Proxy (scale −0.329 to 1.000)						
Baseline	0.38±0.41	57 (37.7)	0.35±0.41	60 (39.7)	−0.03 (−0.15 to 0.09)	0.462
7 days	0.75±0.21	66 (43.7)	0.87±0.21	50 (33.1)	0.12 (0.06 to 0.18)	<0.001
1 month	0.94±0.11	76 (50.3)	0.92±0.18	64 (42.4)	−0.02 (−0.06 to 0.04)	0.810
6 months	0.96±0.12	69 (45.7)	0.95±0.10	78 (51.7)	−0.02 (−0.05 to 0.02)	0.033
1 year	0.96±0.10	75 (49.7)	0.92±0.16	72 (47.7)	−0.04 (−0.08 to 0.00)	0.044
EQ-5D-3L Proxy visual analogue scale (scale 0 to 100)						
Baseline	55.2±24.1	63 (41.7)	55.5±21.1	64 (42.4)	0.3 (−6.5 to 7.1)	0.826
7 days	77.0±18.1	65 (43.0)	81.7±15.7	50 (33.1)	4.7 (−0.23 to 9.6)	0.062
1 month	89.8±9.5	72 (47.7)	86.8±14.5	64 (42.4)	−3.0 (−6.7 to 0.7)	0.370
6 months	91.5±9.7	69 (45.7)	89.8±9.8	78 (48.3)	−1.7 (−4.8 to 1.4)	0.275
1 year	91.5±8.5	75 (49.7)	90.6±10.7	71 (47.0)	−0.9 (−2.1 to 3.9)	0.993

CI, confidence interval; EQ-5D-3L, European Quality of Life-5 Dimensions 3 Level questionnaire; EQ-5D-Y-3L, European Quality of Life-5 Dimensions-Youth 3 Level questionnaire; SD, standard deviation.

According to the results of the Net Promoter Score, substantially more participants and parents would recommend operative treatment than the non-operative treatment strategy, one month, six months, and one year after treatment (table 7). The Patient Satisfaction Questionnaire-18 showed no significant differences between the two groups (online supplemental table S3).

**Table 7:** Patient satisfaction, measured with the Net Promoter Score, in the appendectomy and non-operative treatment strategy groups

	**Appendectomy group** (n=151)		**Non-operative treatment strategy group** (n=151)		Mean difference (95% CI)	P value
	Mean±SD Net Promoter Score (%)	Missing data (No (%))	**Mean±SD Net Promoter Score** (%)	Missing data (No (%))		
7 days	20.2±5.3	67 (44.4)	18.8±5.0	50 (33.1)	−1.4 (−0.1 to 2.9)	0.81
1 month	36.2±5.6	71 (47.0)	23.9±5.3	63 (41.7)	−12.3 (−14.0 to −10.7)	<0.001
6 months	29.9±5.2	74 (49.0)	20.3±4.7	77 (51.0)	−9.6 (−14.4 to −4.8)	<0.001
1 year	48.0±5.8	76 (50.3)	18.9±4.6	77 (51.0)	−29.1 (−30.8 to −27.4)	<0.001

CI, confidence interval; SD, standard deviation.

We found no significant difference in the number of days absent from school, social, or sports events at the one year follow-up, or in the number of days that parents were absent from work at the one year follow-up between the two groups (table 5). Apart from visits according to the study protocol, participants in the non-operative treatment strategy group visited the emergency department or outpatient clinic more frequently than the appendectomy group (mean difference 0.3, 95% CI 0.1 to 0.4 visits/participant) (table 8). Both the initial and total lengths of hospital stay were significantly longer in the non-operative treatment strategy group (mean differences 1.2 days, 95% CI 0.9 to 1.5 and 1.5 days, 1.1 to 1.9, respectively; table 5).

**Table 8:** Extra visits to the emergency department or outpatient clinic after one year of follow-up in the appendectomy and non-operative treatment strategy groups

Visit	Appendectomy group (n=151)	Non-operative treatment strategy group (n=151)
One extra visit	5 (3.3)	20 (13.2)
Two extra visits	1 (0.7)	7 (4.6)
Three extra visits	0 (0.0)	2 (1.3)
Four extra visits	0 (0.0)	1 (0.7)
Five extra visits	0 (0.0)	1 (0.7)

Data are number (%).

### Secondary outcomes: cost related endpoints

Direct medical costs and societal costs measured at the one year follow-up were significantly lower in the non-operative treatment strategy group than in the appendectomy group (mean differences €−767 (£−668; US$−902), 95% CI −320 to −1214, and €−874, −118 to −1631, respectively; table 9).

**Table 9:** Healthcare costs at the one year follow-up in the appendectomy and non-operative treatment strategy groups

Costs	**Appendectomy group** (n=151)		**Non-operative treatment strategy group** (n=151)		Mean difference (95% CI)	P value
	Mean±SD costs (€)*	Missing data (No (%))	Mean±SD costs (€)*	Missing data (No (%))		
Medical costs	4463.21±1469.17	0 (0.0)	3696.29±2372.86	0 (0.0)	−766.92 (−319.97 to −1213.87)	<0.001
Non-medical costs	1516.99±2713.28	21 (13.9)	1441.54±1439.76	13 (8.6)	−75.45 (−593.70 to 442.81)	0.36
Societal costs	6046.06±3196.68	21 (13.9)	5171.64±3089.89	13 (8.6)	−874.41 (−118.26 to −1630.56)	0.007

* 1€ (£0.87; US$1.18).

CI, confidence interval; SD, standard deviation.

## Discussion

### Principal findings

In this trial, we found that the non-inferiority of the non-operative treatment strategy for simple appendicitis in children, in relation to the proportion of participants with complications, could not be proven in terms of statistical significance. Nevertheless, the observed difference in proportions was only marginally higher. Based on a non-inferiority margin of 5%, the evidence indicated (probability 91%) that the non-operative treatment strategy group had a ≤5% increase in the risk of complications, but this risk was not significant (P=0.093). A non-operative treatment strategy avoided appendectomy in about 70% of participants and was associated with lower direct and societal costs at the one year follow-up. In contrast, appendectomy resulted in a shorter hospital stay, fewer emergency department and outpatient visits, greater use of analgesics, higher patient satisfaction, and the avoidance of recurrent disease. Because both strategies had distinct advantages and disadvantages but resulted in similar health related quality of life at the one year follow-up, shared decision making should have a central role in the future management of children with simple appendicitis.

### Comparison with other studies

Our findings align with those of a recently published individual patient data meta-analysis in adults, showing comparable complication rates between non-operative treatment and appendectomy (5.4% *v* 8.3%).^12^ In the paediatric population, however, published evidence remains inconsistent. A recently published paediatric randomised controlled trial of 222 patients showed that the non-inferiority of a non-operative treatment strategy compared with appendectomy in terms of complications (95.6% of patients had no complications in the appendectomy group *v* 87.4% in the non-operative treatment strategy group; proportion difference −0.082, 95% CI −0.142 to infinity).^14^ Another international randomised controlled trial of 936 children reported a higher complication rate for a non-operative treatment strategy than for appendectomy (relative risk 4.3, 95% CI 2.1 to 8.7).^13^

These discrepancies emphasise the additional value of our randomised controlled trial to the ongoing debate on this topic. Comparability between trials remains challenging because of differences in study population, (primary) outcome, (outcome) definitions, and set non-inferiority margins. In the trial of St Peter et al, imaging confirmation to diagnose appendicitis was not mandatory, potentially affecting diagnostic accuracy and population homogeneity.^13^ Despite these differences, the overall conclusion that the non-inferiority of non-operative treatment could not be shown aligns with other randomised controlled trials and our trial.^11 13 14 38^ Although we did not show non-inferiority in our trial, the 95% CI and probability assessment clearly showed that the differences were only marginal (upper limit of the 97.5% CI was 7.1%, but this value was an extreme value). The confidence or probability that the non-operative treatment strategy caused an increase in the proportion of complications of ≤5% was 91%. Moreover, non-inferiority margins in paediatric trials were ≤20%, often for composite or surrogate primary outcomes, whereas our trial applied a more conservative margin of 5%.^11 13 14 38^ When evaluating complications as the primary outcome, the total number of complications and their severity should also be considered. In our trial, the two treatment strategies had the same total number of complications at the one year follow-up (n=14). Minor complications occurred more frequently after non-operative treatment, whereas major complications were more common after appendectomy, but the differences were small. These findings suggest that complication rates are probably comparable between strategies, despite the fact that the non-inferiority of the non-operative treatment strategy could not be proved significantly.

A key distinction between our trial and previous randomised controlled trials was in the selection and definition of the primary outcome. In the trial by St Peter et al, treatment failure was defined as the need for appendectomy in the non-operative treatment group or the removal of a non-inflamed appendix in the appendectomy group.^13^ In contrast, the Australian randomised trial defined treatment failure as unplanned or unnecessary surgery and associated complications. Unplanned surgery referred to operative intervention in children assigned to non-operative treatment or additional surgery in those assigned to appendectomy, whereas unnecessary surgery was defined as resection of a histopathologically normal appendix.^14^ Although the APAC trial started before the publication of the core outcome set for simple appendicitis in children, the trial adhered to all 12 key outcomes. Unfortunately, no appropriate primary outcome was designated, and outcome definitions were not provided in the core outcome set study.^24^ Therefore, variability between published trials and thus reduced comparability remains a concern.

The primary outcome of the APAC trial was defined as the proportion of patients with complications, rather than treatment failure or success. We considered a non-operative treatment strategy for simple appendicitis in children as the initial step in a step-up approach, reserving appendectomy for children that did not respond to antibiotics or with recurrent disease. From this perspective, early failure and recurrent disease were regarded as acceptable elements of the strategy if we could avoid unnecessary surgery in a substantial portion of children. We acknowledge that both early failure and recurrent appendicitis are clear disadvantages of a non-operative treatment strategy and should be carefully evaluated, discussed, and considered in the ongoing debate of its implementation in the treatment of simple appendicitis in children. We therefore reported these outcomes separately to enhance transparency and interpretability. Classification of adverse events also varied across trials. For example, St Peter et al classified the occurrence of gastrointestinal distress after antibiotic administration as a complication, although whether this assessment is fair or whether it should be seen as treatment related side effects is debatable. In the APAC trial, complications were predefined in the published protocol and adjudicated by an independent committee, enhancing the consistency and internal validity of our primary outcome.^16^

Reported recurrence rates after non-operative treatment for simple appendicitis range from 30% to 45% within a year, contributing to hesitancy in adopting non-operative treatment and reinforcing appendectomy as the standard of care.^13 14 39^ Viewed differently, these data indicate that about 70% of children treated with non-operative treatment avoid surgery altogether. This finding has potential benefits for patients and families by reducing the physical and psychological burden of surgery, as well as for health systems, through lower healthcare utilisation and costs. Definitive interpretation, however, requires confirmation that outcomes after delayed appendectomy are comparable with those after primary appendectomy, and should acknowledge that delayed surgery necessitates readmission to hospital with financial and psychosocial consequences.

Patient and parent preferences are central to defining the role of non-operative treatment in paediatric appendicitis. In adults, many patients report a willingness to accept recurrence risks approaching 50% to avoid appendectomy.^40^ Similarly, in qualitative studies of paediatric appendicitis, 59% of parents expressed a preference for non-operative treatment over surgery.^41^ Hesitancy towards non-operative treatment has been attributed in part to its perceived novelty and to limited discussion of non-operative options during clinical decision making, with appendectomy often presented as the only treatment choice.^42^ These findings underscore the patient demand for less invasive alternatives to diseases that historically were treated surgically. From this perspective, non-operative treatment may be best conceptualised as the initial step in a step-up treatment strategy, with appendectomy reserved for patients who fail antibiotic treatment or have a recurrence. Rather than a binary success or failure strategy, this approach positions non-operative treatment as a staged clinical pathway, aimed at minimising unnecessary surgery while ensuring timely escalation of care when needed. This approach aligns with the principle of primum non nocere and is further supported by emerging insights into appendiceal biology. Once regarded as vestigial, the appendix has been hypothesised to function as a reservoir for commensal gut microbiota with potential immunomodulatory roles.^43 44^ Ongoing debate about associations between appendectomy and inflammatory bowel disease further supports a cautious approach to appendiceal removal unless clearly indicated.^45^

Future efforts to optimise outcomes of non-operative treatment should prioritise refined patient selection and protocol optimisation to better identify children most likely to benefit from non-operative treatment. Protocol refinement should look at the necessity for an appendectomy for recurrent appendicitis or that another antibiotic course may provide comparable efficacy, analogous to the CODA (Comparison of Outcomes of Antibiotic Drugs and Appendectomy) trial strategy and management of recurrent diverticulitis.^9 46^

The need for intravenous administration should also be reviewed because some adult trials have reported favourable outcomes with oral antibiotic regimens. More progressively, a recent randomised controlled trial reported promising results with supportive care alone, omitting antibiotics entirely.^47^ Ongoing randomised controlled trials in adults, including APPAC IV, are looking at this question, but paediatric data on the effectiveness of oral antibiotics or supportive care for simple appendicitis are not yet available.^48^ This evidence gap underscores the need for dedicated paediatric trials, which may also help mitigate concerns about antimicrobial resistance. Reducing the use of antibiotics in childhood may be particularly important given emerging evidence linking early life antibiotic use to an increased risk of developing inflammatory bowel disease later in life.^49^

An important benefit of the appendectomy strategy is the ability to identify unexpected intraoperative and histopathological findings, particularly neuroendocrine tumours, although these tumours are extremely rare and seldom alter clinical management.^39 50^ Other advantages of the appendectomy strategy in the APAC trial included fewer visits to outpatient and emergency departments, and shorter hospital stay than the non-operative treatment strategy. Although these advantages have been confirmed in other studies, these data should be interpreted with caution because these advantages may be a logical consequence of the non-operative treatment strategy protocol used because of safety concerns.^11 38^ With protocol refinements, some of the identified advantages found in the appendectomy group may be reduced.

In the APAC trial, health related quality of life measured by the Child Health Questionnaire-Child Form 87 was similar between the non-operative and appendectomy groups, with small differences of uncertain clinical significance in the scores on the European Quality of Life-5 Dimensions questionnaire, warranting cautious interpretation, especially due to missing data. Similarity in health related quality of life between treatment strategies was also reported in a large preference study at the one year follow-up.^11^ Disability days were comparable, contrasting with previous studies showing fewer disability days with non-operative treatment.^14^ Patients treated non-operatively had more outpatient or emergency healthcare visits, likely reflecting parental vigilance about recurrence. Patient satisfaction scores favoured appendectomy, possibly because of immediate symptom resolution and avoiding potential disease recurrence. Interpretation is limited, however, by missing data and heterogeneity in measurement instruments.

### Limitations of this study

Several limitations of our study merit consideration. Only about 20% of eligible children were enrolled, potentially introducing selection bias. Reasons for non-participation were not systematically captured, but another study performed in our recruitment phase indicated that ±60% of families favoured appendectomy.^11^ Limited familiarity with non-operative treatment may have influenced participation. Methodologically, non-inferiority trials are more complex than conventional superiority designs, requiring careful specification of non-inferiority margins and assumptions about expected group differences. All appropriate and mandatory measures were taken to deal with these challenges in the APAC trial. A distinctive feature of non-inferiority trials is the importance of an explicit assumption on the expected differences. At the beginning of the APAC trial, we had solid arguments to hypothesise lower complication rates with non-operative treatment, but we acknowledge that this assumption may have been proven to be incorrect. Health related quality of life and patient satisfaction outcomes were limited by substantial missing data (about 50%), despite rigorous follow-up. Finally, the one year follow-up aligned with other paediatric trials, but longer term recurrence remains a consideration in decision making. Available long term data suggest that delayed appendectomy beyond two years is uncommon (0-5%).^51 52^

### Conclusions

Non-inferiority of non-operative treatment could not be proven significantly in the APAC trial in children aged 7-17 years with simple appendicitis, in terms of complications at the one year follow-up. Differences in the primary outcome were small, however, and most of the 95% CI was within the predetermined non-inferiority margin. Non-operative treatment avoided surgery in 70% of participants and significantly reduced healthcare costs, at the expense of potential recurrence and greater healthcare utilisation. Overall, a non-operative treatment strategy adds a new and valuable option in the treatment of simple appendicitis in children. Non-operative treatment enhances shared decision making by offering the option to discuss the identified advantages and disadvantages of both treatment strategies and should thus have a key role in the treatment of children with acute simple appendicitis.

## Supplementary material

10.1136/bmjmed-2025-002466 online supplemental file 1

10.1136/bmjmed-2025-002466 online supplemental file 2

10.1136/bmjmed-2025-002466 online supplemental file 3

## Data Availability

Data are available upon reasonable request.
