# Supplementary material for: Non-operative treatment strategy versus surgery for children with simple appendicitis: non-inferiority randomised controlled trial
Source: BMJ Med. 2026 May 13;5(1):e002466. doi: 10.1136/bmjmed-2025-002466 (PMC13182491; doi:10.1136/bmjmed-2025-002466)
Supplement: online supplemental file 1 [file bmjmed-5-1-s001.pdf]

Initial non-operative treatment strategy versus appendectomy treatment strategy for simple appendicitis in children aged 7-17 years. Antibiotics versus Primary Appendectomy in Children with simple appendicitis; APAC study

PROTOCOL TITLE

"Initial non-operative treatment strategy versus appendectomy treatment strategy for simple appendicitis in children aged 7-17 years. Antibiotics versus Primary Appendectomy in Children with simple appendicitis; APAC study"

**Protocol ID**	**APAC 2016**
**Short title**	**Initial non-operative treatment for acute simple appendicitis in children**
**EudraCT number**	2016-003052-70
**Version**	**2.3**
**Date**	**6-12-2022**
**Coordinating investigator/project leader**	*1.* [redacted]<br>*2.* [redacted]<br>*3.* [redacted]

**Principal investigator(s)**<br>**(in Dutch: hoofdonderzoeker)**<br><br>***Multicenter research: per site***	[redacted]<br><br>[redacted]<br><br>[redacted]<br><br>[redacted]<br><br>[redacted]<br><br>[redacted]<br><br>[redacted]<br><br>[redacted]<br><br>[redacted]

	[redacted]
**Sponsor (in Dutch: verrichter/opdrachtgever)**	***Academic Medical Center of Amsterdam*** ***P.O. box 22660, 1100DD Amsterdam, The Netherlands*** ***Phone: +31 20 566 9111*** ***Fax: +31 20 566 4440***
**Subsidising party**	***ZonMw*** ***Department of pediatric surgery*** ***Emma Children's Hospital AMC & VU medical centre***
**Independent expert (s)**	[redacted]
**Laboratory sites <*if applicable*>**	***N/A***

**Pharmacy <*if applicable*>**	[redacted]

PROTOCOL SIGNATURE SHEET

**Name**	**Signature**	**Date**
**Head of Department:**<br>[redacted]		22/6/2018
**[Coordinating Investigator/Project leader/Principal Investigator]:**<br>[redacted]		22/6/2018
[redacted]		22/6/2018

TABLE OF CONTENTS

1. Introduction and Rationale	13
2. Objectives	16
3. Study design	18
4. Study population	22
5. Treatment of subjects	24
6. Investigational Product	26
7. Methods	28
8. Safety reporting	31
9. Statistical analysis	35
10. Ethical Considerations	36
11. Administrative aspects, monitoring and publication	38
12. Structured Risk Analysis	40
13. References	41
Appendices	44

LIST OF ABBREVIATIONS AND RELEVANT DEFINITIONS

ABR	ABR form, General Assessment and Registration form, is the application form that is required for submission to the accredited Ethics Committee (In Dutch, ABR = Algemene Beoordeling en Registratie)
AE	Adverse Event
AR	Adverse Reaction
CA	Competent Authority
CCMO	Central Committee on Research Involving Human Subjects; in Dutch: Centrale Commissie Mensgebonden Onderzoek
CV	Curriculum Vitae
DSMB	Data Safety Monitoring Board
EU	European Union
EudraCT	European drug regulatory affairs Clinical Trials
GCP	Good Clinical Practice
IB	Investigator's Brochure
IC	Informed Consent
IMP	Investigational Medicinal Product
IMPD	Investigational Medicinal Product Dossier
METC	Medical research ethics committee (MREC); in Dutch: medisch ethische toetsing commissie (METC)
(S)AE	(Serious) Adverse Event
SPC	Summary of Product Characteristics (in Dutch: officiële productinfomatie IB1-tekst)

**Sponsor**	**The sponsor is the party that commissions the organisation or performance of the research, for example a pharmaceutical company, academic hospital, scientific organisation or investigator. A party that provides funding for a study but does not commission it is not regarded as the sponsor, but referred to as a subsidising party.**
**SUSAR**	**Suspected Unexpected Serious Adverse Reaction**
**WBP**	**Personal Data Protection Act (in Dutch: Wet Bescherming Persoonsgevens)**
**WMO**	**Medical Research Involving Human Subjects Act (in Dutch: Wet Medisch-wetenschappelijk Onderzoek met Mensen**

SUMMARY

Rationale:
Initial non-operative treatment of acute simple appendicitis has recently been investigated in both the adult and the paediatric population. In the adult population, six RCTs showed that an appendectomy could be avoided in 40-76% of the patients at the end of the follow-up period of 12 months. Despite the fact that some patients need to undergo a delayed appendectomy, it has been demonstrated in systematic reviews that non-operative treatment strategy is associated with a significant reduction in complications, faster recovery and return to work, shorter pain duration and less analgesic medication consumption. In children only pilot data is yet available. Short-term success rates of this strategy (including our own pilot cohort study) are between the 83-92%. Long-term results (one year follow-up) are available from two studies; 62-75% did not require an appendectomy. No large RCT have yet been conducted in the paediatric population. It is therefore essential to generate high quality empirical evidence regarding this strategy in this subset of patients.

Objective:
To evaluate the effectiveness of initial non-operative treatment strategy (reserving appendectomies for those not responding or with recurrent disease), compared with appendectomy strategy in children from 7 to 17 years old with acute simple appendicitis in terms of proportion of patients experiencing complications, quality of life and costs.

Study design:
An unblinded multi-centre randomized controlled non-inferiority trial with a 1:1 block randomization stratified by hospital will be performed.

Study population:
Eligible for inclusion are all children from 7 to 17 years old, inclusive, with a radiologically confirmed simple appendicitis. Definition of simple appendicitis is based upon predefined clinical and radiological criteria.

We will exclude patients with:
- Generalized peritonitis, (suspicion of) complex appendicitis based upon clinical prediction model and/or radiological evaluation or with signs of septicaemia
- Faecolith on ultrasonography

- Serious co-morbidity
- Recurrent appendicitis
- Suspicion of an underlying malignancy or inflammatory bowel disease
- Documented type 1 allergy to the antibiotics used.

Intervention:

Intervention group (Non-operative treatment strategy): Clinical observation for 48 hours with administration of Intravenous administration of amoxicillin/clavulanic acid 25/2.5mg 6-hourly (total 100/10 mg/kg daily; maximum 6000/600mg a day) and gentamicin 7mg/kg once daily for 48 hours. If after 48 hours the patient fulfils the predefined discharge criteria, the antibiotics will be switched to oral amoxicillin/clavulanic acid 50/12.5 mg/kg 8-hourly (max 1500/375mg a day) for in total 7 days and discharge. An appendectomy is reserved for those patients with clinical deterioration, non-improvement after 72 hours or recurrent appendicitis.

Control group (Operative treatment strategy): Clinical observation and semi-urgent appendectomy. Pre-, peri- and postoperative care according to local protocol. No routine postoperative antibiotics. Discharge when the patient fulfils the predefined discharge criteria.

Main study parameters/endpoints:

Primary (patient level): The proportion of patients experiencing complications within one year-follow up.

Secondary:

Number of days absent from school, social or sport events (patient-level)

Number of days absent from work (parents-level)

Total number of extra visits (not the already scheduled ones) to the outpatient clinic, general practitioner's office or emergency department for abdominal pain.

Total length of hospital stay during the follow-up period for strategy related treatment or complications

Level of pain (measured by the validated Visual Analogue Scale) Pain medication utilization during the first seven days after admission

Proportion of patients with missed diagnosis of complex appendicitis with risk of peritonitis

Proportion of patients not having to undergone appendectomy

Proportion of patients experiencing recurrent appendicitis within one-year follow-up.

Recurrent appendicitis is defined as those patients with a clinical and radiological

high suspicion of recurrent appendicitis who undergo an appendectomy and histopathological examination confirms the diagnosis of recurrent appendicitis

Proportion of patients experiencing early failure of initial non-operative treatment.

Early failure is defined as all patients that undergo an appendectomy during the antibiotic course (iv or oral) due to persistent complaints, clinical deterioration or faecolith.

Proportion of patients that undergo interval appendectomy.

Interval appendectomy is defined as those patients that undergo an appendectomy with a clinical and radiological low suspicion of recurrent appendicitis. Histopathological examination shows no signs of recurrent appendicitis.

Proportion of patients experiencing complications after: discharge, seven days, one month and six months after treatment.

Quality of life measured by the validated CHQ-CF87, EQ-5d-Youth, EQ-5d Proxy questionnaire.

Medical, non-medical and indirect costs at one year follow up of the treatment strategy measured by the iMCQ and iPCQ (adapted to children and parents) and gathered actual health care cost.

Patient satisfaction measured by the NET PROMOTOR SCORE and validated Patient Satisfaction Questionnaire (PSQ)18.

Factors influencing implementability

Time of measurement:

- Discharge
- Seven days
- One, six and twelve months

Nature and extent of the burden and risks associated with participation, benefit and group relatedness:

1. Risks of participation:

- Specific non-operative treatment strategy: possible need for delayed operation (10-25%), recurrent appendicitis (10%).
- Both strategies**:** Allergic reaction to antibiotics (<1%), known appendectomy associated complications (5-10%).

2. Burden of participation:

- Specific non-operative treatment strategy: Extra admission day (in comparison with appendectomy), ultrasonography after 48 hours (extra non-invasive procedure), Blood samples after 24 and 48 hours for determination of C-reactive protein / Leucocytes (2x extra a 1 cc). Blood samples will be obtained through the already placed IV access. In case this is not successful, an extra venapunction will be performed.

- Both strategies:
  - Telephone interviews/Email (3x5minutes=15 minutes).
  - Filling out questionnaires (1x only QOL, 4x QOL, PSQ-18 and net promotor scale, 3x IMCQ and IPCQ).
  - Duration of filling out questionnaires:
    - QOL: 10 minutes (5x10=50 minutes)
    - iMCQ and iPCQ: 10 minutes (3x10=30 minutes)
    - PSQ/Net premotor scale: 5 minutes (4x5=20 minutes)
    - Total time per participant in one year follow up: 100 minutes

3.Benefit of non-operative treatment strategy: Avoidance of surgery (75-90%) and its related early and late morbidity, potential better quality of life.

1. INTRODUCTION AND RATIONALE

Acute appendicitis is a common disease affecting approximately 1 per 1000 inhabitants each year. [1] Rare below the age of one year, the peak incidence of acute appendicitis is around adolescence. The standard of care for acute appendicitis has been an appendectomy ever since Fitz's report in 1886. [2] Therefore in 2010, more than 15,000 appendectomies were performed in the Netherlands, of which 5,500 in patients younger than 20 years of age. [1] The rationale for the appendectomy was based upon the entrenched idea that appendicitis is an irreversible progressive disease starting as an simple inflammation and evolving in time to a potentially lethal disease with necrosis and perforation. Removal of a healthy appendix was therefore never seen as an undesirable let alone adverse event. Moreover, the risk of post-operative complications was considered to outweigh the potential risk of perforation.

Recent studies, however, have questioned the progressive nature of appendicitis. Epidemiological, imaging and pathology studies demonstrate that not all patients with appendicitis will develop complex appendicitis over time. [3-8] Rather, it appears that there are two types of appendicitis, simple, which can resolve spontaneously or with non-operative treatment, and does not progress to perforation, and complex, in which perforation usually occurs before arrival at the hospital. Due to the increased use and experience in imaging modalities and clinical experience it has been shown that we are able to distinguish between simple and complex appendicitis in over 90% of the patients [9-13]

This change in concept of the pathogenesis of appendicitis has led to the debate regarding the necessity of an appendectomy for all patients with acute appendicitis. Non-operative treatment strategy has recently been investigated in the adult population. [14-18] This is however not new as the first report of non-operative treatment for acute appendicitis was already in 1959. Potential benefits of this strategy are avoidance of surgery and its risk of complications, better quality of life and reduced costs.

Adult population

In the adult population, several large RCTs have been conducted. These studies demonstrated that 48-85% of the patients with simple appendicitis did not require surgery at one-year follow up. [14-18] Subsequently, meta-analyses have been performed evaluating these RCTs (and also some cohort studies). [19-22] Interpretation from these studies however should be with care as most RCTs are subject to methodological flaws, usage of poor definitions for diagnosis and outcome and show large heterogeneity. Still some preliminary conclusions can be made.

First of all, as already stated above, in 48-85% of the patients with acute appendicitis surgery was avoided at one-year follow up. [14-18] Therefore non-operative treatment reduces the number of appendectomies. Secondly, as a result of the reduction in appendectomies, it appears that initial non-operative treatment is able to reduce the complication rate with 31-48%. [19-22] Especially major complications have been reduced. Thirdly other potential benefits of initial non-operative treatment reported are: decreased utilization of pain medication, earlier return to work and reduced costs. [19-22]

Paediatric population

In the paediatric population, five studies have reported the outcome of initial non-operative treatment for acute simple appendicitis in children at one-year follow-up. [23-27] They included 147 children for non-operative treatment strategy and 173 children for immediate appendectomy strategy. [23-27] At one-year follow-up 73% of the 147 children did not require surgery. [23-27] The range of complications was 0-12.5% and 0-16.7% for the initial non-operative treatment strategy and immediate appendectomy strategy, respectively. [23-27] Other secondary potential benefits reported in these studies are reduction of costs and better quality of life. [23-27]

Based upon the studies in both the adult and paediatric population we can conclude that:

1. Initial non-operative treatment strategy is able to avoid an appendectomy in a significant proportion of patients. This strategy is 100% effective in treating patients with acute simple appendicitis, since patients are monitored closely and appendicitis is performed when the clinical situation does not improve or deteriorates
2. Initial non-operative treatment strategy is able to reduce the complication rate.
3. Initial non-operative treatment strategy might be associated with a reduction of costs, decreased use of pain medication and better quality of life

Based upon these results, we have conducted a prospective, multicentre pilot cohort study (NL38141.029.11) in which children from 7-17 years old with a radiologically proven simple appendicitis were offered initial non-operative treatment strategy. [28] Twenty-five of the 44 eligible patients participated. Reasons for non-participation were mainly based upon the fact that there was explicit wish for surgery by the parents in some cases. Main conclusions from this study were:

1. Success rate of non-operative treatment alone was 92% at 8 weeks follow up
2. A large scale RCT regarding this topic is feasible as the inclusion rate [95%CI] of 57 [42-70]% was significantly higher than the benchmark of 33%. [28]

Time has come to investigate these two strategies in a large national RCT in the paediatric population as one of the first in the world. The main aim of this study is to generate high-quality empirical evidence for the effectiveness of initial non-operative treatment strategy (reserving appendectomies for those not responding or with recurrent disease), compared with immediate appendectomy in children from 7 to 17 years old with acute simple appendicitis in terms of complications, quality of life and costs

2. OBJECTIVES

Primary Objective:

- To investigate the difference in proportion of children experiencing complications within one year between the initial non-operative treatment strategy and direct appendectomy strategy for acute simple appendicitis in children aged 7-17 years.

Secondary Objective(s):

- To investigate the difference in the total numbers of days absent from school, sporting – or social events (child) within one year between the initial non-operative treatment strategy and direct appendectomy strategy for acute simple appendicitis in children aged 7-17 years. Days absent counted are those days associated with treatment for acute appendicitis or treatment related complications or recurrent abdominal pain.
- To investigate the difference in the total numbers of days absent from work (parents) within one year between the initial non-operative treatment strategy and direct appendectomy strategy for acute simple appendicitis in children aged 7-17 years. Days absent counted are those days associated with treatment for acute appendicitis or treatment related complications or recurrent abdominal pain.
- To investigate the difference in level of pain measured by the Visual Analogue Scale during the first seven days of treatment between the initial non-operative treatment strategy and direct appendectomy strategy for acute simple appendicitis in children aged 7-17 years.
- To investigate the difference in pain medication utilization during the first seven days of treatment between the initial non-operative treatment strategy and direct appendectomy strategy for acute simple appendicitis in children aged 7-17 years.
- To investigate the proportion of patients experiencing early failure and recurrent appendicitis after initial non-operative treatment strategy for acute simple appendicitis in children aged 7-17 years.
- To investigate the proportion of patients not having to undergo an appendectomy after initial non-operative treatment strategy for acute simple appendicitis in children aged 7-17 years at one month, six months and one year follow up.
- To investigate the difference in quality of life during one year between the initial non-operative treatment strategy and direct appendectomy strategy for acute simple appendicitis in children aged 7-17 years.
- To investigate the difference in medical, non-medical and indirect costs during one year between the initial non-operative treatment strategy and direct appendectomy strategy for acute simple appendicitis in children aged 7-17 years.
- To investigate the obstructing and promoting factors for (de-) implementation of one of the strategies in the current (paediatric) surgical practice in the Netherlands.
- To investigate the difference in patient satisfaction during one year between the initial non-operative treatment strategy and direct appendectomy strategy for acute simple appendicitis in children aged 7-17 years.

3. STUDY DESIGN

An unblinded multi-centre randomized controlled trial (RCT) will be conducted to investigate the primary and secondary objectives mentioned in the previous section. Children, aged 7-17 years, inclusive, with a radiologically proven acute simple appendicitis are eligible for inclusion. If the patient does not fulfil the exclusion criteria, patients and parents will be asked for informed consent prior to randomization. In case of non-participation in the randomized trial, immediate appendectomy is offered as standard care, and patient and parents will be asked for consent for prospective collection of their data. In addition, the reason for non-participation will be asked and recorded.

After informed consent for participation in the randomized trial, patients will be randomized using a computer/internet based randomization program. A 1:1 variable block randomization stratified by hospital will be performed. Complete concealment of randomization sequence is warranted. Patients can be randomized to either the initial non-operative treatment strategy or the immediate operative treatment strategy. To clarify the process of informed consent and randomization a flow diagram has been added.

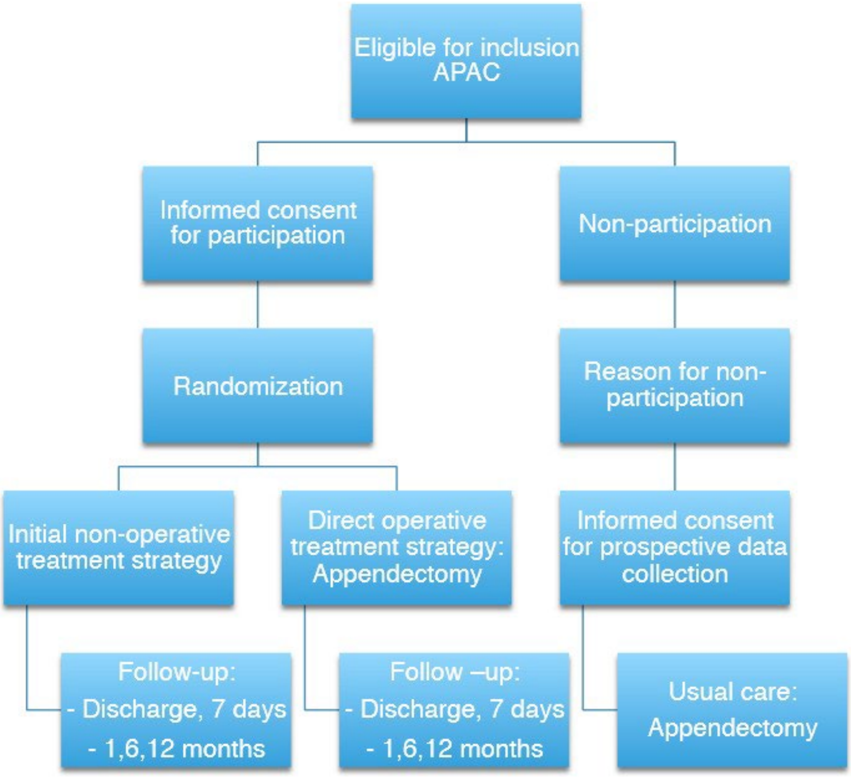

The treatment strategies will consist of the following key elements.

Initial non-operative treatment strategy

1. Admission of patients under paediatric surgical responsibility and
2. Evaluation of current health related quality of life measured by (CHQ-CF87, EQ-5d-Youth, EQ-5d-Proxy) and level of pain measured by Visual Analogue Scale (VAS)
3. Administration of intravenous antibiotics (amoxicillin/clavulanic acid 25/2.5 mg/kg 6-hourly (total 100/10mg/kg daily; maximum doses: 6000/600mg a day) and gentamicin 7mg/kg once daily) for the first 48 hours (See SPC Appendix A/B).
4. Every 6 hours evaluation by nurse:
    - Level of pain measured by Visual Analogue Scale (VAS) compared to level at admission, despite adequate pain medication
    - Blood pressure, heart rate, temperature.
    - Nausea, vomiting, urine production
        - In case of signs of clinical deterioration: contact physician
5. Every 12 hours evaluation by physician in order to detect clinical deterioration defined as:
    - Increasing level of pain (on VAS compared to level at admission, despite adequate pain medication)
    - Signs of generalized peritonitis, defined as:

      Diffuse inflammation of the peritoneum with clinical signs consisting of increasing abdominal pain, generalized tenderness, diffuse abdominal rigidity, sinus tachycardia, and signs of paralytic ileus.
    - Persistent elevated temperature >38.5 degree Celsius (>24 hours)
    - Persistent vomiting (>24 hours)
    - Signs of sepsis or organ failure (see appendix C) [29]
    - After every assessment, evaluation if patient's condition is deteriorating (see appendix D)
6. After 24 hours (in addition to clinical evaluation)
    - Biochemical tests: Infection parameters (C-Reactive Protein in mg/L, Leucocytes x10^9/L)
7. After 48 hours (in addition to clinical evaluation)
    - Biochemical tests: infection parameters C-Reactive Protein in mg/L, Leucocytes x10^9/L)
    - Ultrasonography (see appendix E)
    - Evaluation of predefined discharge criteria (see appendix F)
        1. Body temperature <38 degrees Celsius
        2. VAS score <4

   3. Oral intake adequate (No nausea or vomiting)
   4. Able to mobilize
   5. Decreasing leucocytosis
   6. Decreasing C-reactive protein
   7. No signs of complex appendicitis on ultrasound
   8. Consent of parents for discharge

8. If after 48 hours a faecolith is noted on the ultrasound, the patient and parents will be advised to undergo an appendectomy, as the risk of recurrent appendicitis is high.
9. If after 48 hours the patient meets the predefined discharge criteria, the antibiotic will be changed to oral administration of amoxicillin/clavulanic acid 50/12.5 mg/kg in three doses (maximum doses: 1500/375mg a day). If after 48 hours, the patient does not meet the predefined discharge criteria yet, intravenous administration will be continued to a maximum of 72 hours.
10. If administration is changed to oral antibiotics, the patient will be discharged with medication for a total of seven days.
11. If after 72 hours of intravenous administration of antibiotics, the patient does not meet the predefined discharge criteria, an appendectomy will be performed.
12. Follow-up by telephone at 7 days [Range 5-9 days after discharge] (Detection of early failure, level of pain measured by Visual Analogue Scale and pain medication utilization, to evaluate quality of life (CHQ-CF87, EQ-5d-Youth, EQ-5d-Proxy),, complications, days absent from work/school etcetera, patient satisfaction (PSQ-18, Net promoter scale))
13. Out-patient follow up at 1 month [Range 2-6 weeks after discharge] (usual care so this is no extra visit) (to evaluate quality of life (CHQ-CF87, EQ-5d-Youth, EQ-5d-Proxy), costs (iMCQ and iPCQ which were altered to fit the situation of a child and the situation of indirect costs resulting from decreased productivity of parents), complications, days absent from work/school etcetera, patient satisfaction (PSQ-18, Net promoter scale))
14. Follow up by telephone and email at 6 [Range 5-7 months after discharge] and 12 [Range 11-13 months after discharge] months after treatment for acute simple appendicitis. (to evaluate quality of life (CHQ-CF87, EQ-5d-Youth, EQ-5d-Proxy), costs (altered iMCQ and iPCQ), complications, days absent from work/school etcetera, patient satisfaction (PSQ-18, Net promoter scale))

In case of clinical deterioration, the decision can be made to proceed with urgent appendectomy or to perform additional imaging studies according to the discretion of the surgeon in charge of the patient. All decisions will be recorded in the case report form of each patient.

Immediate operative treatment strategy.

1. Admission of patients under paediatric surgical responsibility and
2. Evaluation of current health related quality of life measured by (CHQ-CF87, EQ-5d-Youth, EQ-5d-Proxy) and level of pain measured by Visual Analogue Scale (VAS)
3. Semi-urgent appendectomy (according to local protocol and in line with national guideline) [30]
4. Approach (open or laparoscopic) will depend on surgeon's preference
5. Pre-operatively antibiotic prophylaxis
6. Stump closure: Ligature (open), endoloops/endostaplers (laparoscopic)
7. The concept of abdominal wall protection is followed when extracting the appendix.
8. After appendectomy, the appendix will be sent to the department of pathology for routine histopathological examination.
9. Postoperative antibiotics only when indicated (due to the fact that the patient suffered from a complex appendicitis)
10. Patients will be discharged without antibiotics when they fulfil the same predefined criteria as above (appendix F).
11. Follow-up by telephone at 7 days [Range 5-9 days after discharge] (Detection of early failure, level of pain measured by Visual Analogue Scale and pain medication utilization, to evaluate quality of life (CHQ-CF87, EQ-5d-Youth, EQ-5d-Proxy), complications, days absent from work/school etcetera, patient satisfaction (PSQ-18, Net promoter scale))
12. Out-patient follow up at 1 month [Range 2-6 weeks after discharge] (usual care so this is no extra visit) (to evaluate quality of life (CHQ-CF87, EQ-5d-Youth, EQ-5d-Proxy), costs (iMCQ and iPCQ which were altered to fit the situation of a child and the situation of indirect costs resulting from decreased productivity of parents), complications, days absent from work/school etcetera, patient satisfaction (PSQ-18, Net promoter scale))
13. Follow up by telephone and email at 6 [Range 5-7 months after discharge] and 12 [11-13 months after discharge] months after treatment for acute simple appendicitis. (to evaluate quality of life (CHQ-CF87, EQ-5d-Youth, EQ-5d-Proxy), costs (altered iMCQ and iPCQ), complications, days absent from work/school etcetera, patient satisfaction (PSQ-18, Net promoter scale))

4. STUDY POPULATION

1.1. Population (base)

Acute appendicitis is a common disease affecting approximately 1 per 1000 inhabitants each year. In 2010, 16.000 appendectomies were performed in the Netherlands of which 5500 in patients younger than 20 years old. The distribution of simple and complex appendicitis in our aimed age group (7-17 years old) will probably be 50%-50%. As appendicitis is rare on the extremes of ages (i.e. very young child) around the 2000 children will probably be treated for acute simple appendicitis in the Netherlands.

1.2. Inclusion criteria

In order to be eligible to participate in this study, a subject must meet all of the following criteria:

- Age 7-17 years, inclusive
- Radiologically confirmed simple appendicitis, defined as:
  - a. Clinical findings:
    - i. Unwell, but not generally ill
    - ii. Localized tenderness in the right iliac fossa region
    - iii. Normal/hyperactive bowel sounds
    - iv. No guarding
    - v. No mass palpable
  - b. Ultrasonography (see appendix E):
    - i. Incompressible appendix with an outer diameter of ≥6 mm
    - ii. Hyperaemia within the appendiceal wall
    - iii. Without faecolith
    - iv. Infiltration of surrounding fat
    - v. No signs of perforation
    - vi. No signs of intra-abdominal abscess/phlegmon

1.3. Exclusion criteria

A potential subject who meets any of the following criteria will be excluded from participation in this study:

1. Patients with severe general illness at time of presentation:
   - a. Generalized peritonitis defined as:

     Diffuse inflammation of the peritoneum with clinical signs consisting of increasing abdominal pain, generalized tenderness, diffuse abdominal rigidity, sinus tachycardia, and signs of paralytic ileus.
   - b. Severe sepsis or septic shock, as defined by the international paediatric sepsis

consensus conference. (see appendix C) [29]

   c. Signs of complex appendicitis. For this our own developed clinical prediction rule will be used. (see appendix G) [13]

2. Children with a faecolith on ultrasonography
3. Patients with serious associated conditions or malformations such as:
   a. Congenital or acquired cardiac or pulmonary disease with significant hemodynamic consequences
   b. Immunodeficiency
   c. Malignancy
   d. Homozygous sickle cell disease
   e. Metabolic disorders
4. Patients with documented type 1 allergy to the antibiotics used
5. Patients who have been treated for acute simple appendicitis non-operatively in the past year
6. Patients with a suspicion of an underlying malignancy based upon clinical and radiological evaluation

1.4. **Sample size calculation**

A non-inferiority design will be used based upon evidence in the literature that initial non-operative treatment of appendicitis has many potential secondary advantages. It would be sufficient if this trial demonstrates that the outcome in terms of complications of initial non-operative treatment strategy is not worse than the immediate appendectomy group. Recent studies demonstrated that the overall frequency of post-operative complications is approximately 10%, meaning that 90% will be successfully treated without complications. Initial non-operative is estimated to reduce the complication rate with approximately 50%, meaning that 95% will be successfully treated without complications. Using a 1-sided alpha of 2.5%, we need about 150 patients per group to achieve 90% power to exclude a difference in favour of the usual care group of more than 5%. Although in our pilot the drop-out rate in one year was only 2%, we take into account a drop-out rate of 10%. Therefore, the number of patients to be included is 334.

Addendum 6-12-2022:

In the abovementioned sample size calculation we took into account a drop-out rate of 10%. At this moment in the inclusion phase 276 of 334 (83%) patients are included. 243 of the 334 (73%) have completed the study with 1-year follow-up. There was only one patient lost to follow-up (0.3%). As almost 75% of patients have completed the study, we do not expect a drop-out rate of 10% as described in our initial study

protocol. We discussed with the Data Safety Monitoring Board and our involved senior methodologist how to deal with this negligible loss of follow-up. It was concluded that we can re-calculate our primary outcome with adequate power with a sample size of 302 patients. Therefore we adjusted our definitive sample size accordingly.

5. TREATMENT OF SUBJECTS

5.1 Investigational product/treatment

Initial non-operative treatment (key elements also see section 3)):

- Administration of antibiotics:

  IV: Amoxicillin/clavulanic acid 25/2.5 mg/kg 6-hourly (total 100/10mg/kg daily; maximum doses: 6000/600mg a day) and gentamicin 7mg/kg once daily) for the first 48 hours. Maximum duration 72 hours

  Oral: Amoxicillin/clavulanic acid 50/12.5 mg/kg in three doses (maximum doses: 1500/375mg a day).

  Total Duration: 7 days.
- Diet: No oral intake for the first 12 hours
- IV fluids (see co-intervention)
- Pain medication (see co-intervention)
- Clinical evaluation by nurse every 6 hours and by MD every 12 hours.
- Blood testing (WBC, CRP) every 24 hours
- Repeated ultrasound after 48 hours

Direct appendectomy strategy (key elements also see section 3):

- Diet: No oral intake till after surgery
- IV fluids (see co-intervention)
- Pain medication (see co-intervention)
- Antibiotic prophylaxis: according to local protocol
- Appendectomy: Approach (open or laparoscopic) according to surgeon's preference
- Postoperative care (according to local protocol in line with national guideline)

5.2 Use of co-intervention (if applicable)

- Intravenous administration of fluids (according to local protocol)

  0-10 kg à 100 ml/kg

  10-20 kg à 50 ml/kg extra

  >20 kg à 20 ml/kg

  For instance when a child weights 20 kilogram the daily intake should be

  10x100ml + 10x50 ml à 1.5 liter
- Pain medication (common practice): According to the national pain protocol, management of pain will consist of the following medication (doses adjusted to the www.kinderformularium.nl) [31,32]):

Acetaminophen (i.v.) Start dose: 20 mg/kg. Afterwards: 60 mg/kg/day (in 4 doses)
Acetaminophen (rectal) Start dose: 40 mg/kg. Afterwards: 90 mg/kg/day (in three doses)
Diclofenac 1-3 mg/kg/day (in 3 doses) Morphine (rectal) 1.2-2.4 mg/kg/day (in 6 doses)
Morphine (I.v.) Start dose: 0.1 mg/kg (in 10 minutes. Afterwards continuous administration. Dose:0.25 mg/kg/day

In most cases, acetaminophen either intravenous or rectal, will be administered. When this turns out to be inadequate (defined as a VAS score > 4), additional pain medication will be given following a step-up/step down principle. Starting with diclofenac and in addition morphine. When the VAS score becomes < 4, attempts will be made to remove additional pain medication.

- Own medication: patients are allowed to use their regular prescribed medication, with the exception of antibiotics.

6. Intervention

The EU clinical trial regulation No 536/2014 on clinical trials on medicinal products for human use is accepted by the European Commission on 16 June 2014. It is set to replace the EU clinical trial directive 2001/20/EC. The moment of entry into force of the regulation is currently estimated to occur in 2020 (https://ec.europa.eu/health/human-use/clinical-trials/regulation_en).

In this directive a clinical trial that meets the recruitments of a ‘Low-intervention clinical trial’ and uses an investigational medicinal product that is covered by a marketing authorisation should be subject to less stringent rules, as regards monitoring, requirements for the contents of the master file and traceability of investigational medicinal products.

As this study meets these criteria we will use commercially available products for the intervention. In order to ensure subject safety and the reliability and robustness of data from clinical trials, there are arrangements for traceability, storage and destruction of the investigational medicinal products, these are described in paragraph 6.8.

6.1 Name and description of investigational product(s)

Non-operative treatment:

IV: Amoxicillin/clavulanic acid 25/2.5 mg/kg 6-hourly. Total dose: 100/10 mg/kg/day (max 72 hours)

IV: Gentamicin 7 mg/kg once daily (max 72 hours)
Oral: Amoxicillin/clavulanic acid 50/12.5 mg/kg in three doses (max is 1500/375mg a day)
Total duration antibiotic course: 7days

Operative treatment:

Diet: No oral intake till after surgery IV fluids (see co-intervention)
Pain medication (see co-intervention)
Antibiotic prophylaxis: according to local protocol
Appendectomy: Approach (open or laparoscopic) according to surgeon's preference
Postoperative care (according to local protocol in line with national guideline)

6.2 Summary of findings from non-clinical studies

See appendix SPC amoxicillin/clavulanic acid and appendix SPC gentamicin

6.3 Summary of findings from clinical studies

See appendix SPC amoxicillin/clavulanic acid and appendix SPC gentamicin

6.4 Summary of known and potential risks and benefits

See appendix SPC amoxicillin/clavulanic acid and appendix SPC gentamicin

6.5 Description and justification of route of administration and dosage

See appendix SPC amoxicillin/clavulanic acid and appendix SPC gentamicin

6.6 Dosages, dosage modifications and method of administration

See appendix SPC amoxicillin/clavulanic acid and appendix SPC gentamicin

6.7 Preparation and labelling of Investigational Medicinal Product

All medications used in this RCT are authorized medicinal products. The indication or which they are administrated in this study i.e. appendicitis is within the indication of gastrointestinal infection. We will use commercially available medication as Investigational Medicinal Products and there will be no separate preparation of labelling. Local pharmacies will be checked on adherence to EU Good Distribution Practice Guidelines and arrangements will be made to guarantee drug traceability and drug accountability.

6.8 Drug accountability

As mentioned in the previous paragraph, all medications used are already registered for the indication gastrointestinal infection. In order to ensure drug-accountability (in line with GCP guidelines) pharmacies from the participating hospitals will be involved in this research.

Moreover a general logistic plan will be set up by dr Bet (pharmacy VUmc). A drug accountability form will be made for each patient.

7. METHODS

7.1 Study parameters/endpoints

7.1.1 Main study parameter/endpoint

The proportion of patients experiencing complications within one year-follow up. The adjudication committee, consisting of [redacted] will review all complications/adverse events reported in the trial, to assess their relation with the treatment, as well as the length of hospital stay (secondary outcome measure). Review of the length of hospital stay is necessary to assess their relation with the treatment or potential complications.

Complications are defined as:

- Allergic reaction to antibiotics administered
- Need for other surgical or radiological intervention other than appendectomy but related to appendicitis
- Re-admission for an indication other than recurrent appendicitis
- Complications associated with appendectomy:
  - Superficial Site infection
  - Intra-abdominal abscess
  - Stump leakage/stump appendicitis
  - Secondary Bowel Obstruction
  - Pneumonia
  - Anaesthesia Related complications
  - Hernia cicatricalis
  - Re-admission

7.1.2 Secondary study parameters/endpoints (if applicable)

Number of days absent from school, social or sport events (patient-level)
Number of days absent from work (parents-level)
Total number of extra visits (not the already scheduled ones) to the outpatient clinic, general practitioner's office or emergency department for abdominal pain.
Total length of hospital stay during the follow-up period for strategy related treatment or complications
Level of pain (measured by the validated Visual Analogue Scale)
Pain medication utilization during the first seven days after admission

Proportion of patients with missed diagnosis of complex appendicitis with risk of peritonitis

Proportion of patients not having to undergone appendectomy

Proportion of patients experiencing recurrent appendicitis within one-year follow-up.

Recurrent appendicitis is defined as those patients with a clinical and radiological high suspicion of recurrent appendicitis who undergo an appendectomy and histopathological examination confirms the diagnosis of recurrent appendicitis

Proportion of patients experiencing early failure of initial non-operative treatment.

Early failure is defined as all patients that undergo an appendectomy during the antibiotic course (iv or oral) due to persistent complaints, clinical deterioration or faecolith.

Proportion of patients that undergo interval appendectomy.

Interval appendectomy is defined as those patients that undergo an appendectomy with a clinical and radiological low suspicion of recurrent appendicitis. Histopathological examination shows no signs of recurrent appendicitis.

Proportion of patients experiencing complications after: discharge, seven days, one month and six months after treatment.

Quality of life measured by the validated CHQ-CF87, EQ-5d-Youth, EQ-5d-Proxy questionnaire.

Medical, non-medical and indirect costs at one year follow up of the treatment strategy measured by the iMCQ and iPCQ which where adapted for use in children and parents, plus gathered actual health care cost.

Patient satisfaction measured by the NET PROMOTOR SCORE and validated Patient Satisfaction Questionnaire (PSQ)18.

Factors influencing implementability

Time of measurement:

- Discharge
- Seven days [Range 5-9 days]
- One [Range 2-6 weeks after discharge], six [Range 5-7 months after discharge] and twelve [Range 11-13 months] months

7.1.3 Other study parameters (if applicable)

Baseline/monitoring values:

Month/Year of presentation

Age

Gender

Clinical symptoms
Findings from physical examination
Biochemical testing results
Radiological imaging studies results

7.2 Randomisation, blinding and treatment allocation

This is an unblinded multi-centre RCT. Randomisation will be done using a computer/internet based randomization program (Castor EDC version 4.6; Amsterdam; The Netherlands). A variable 1:1 block randomization stratified by hospital will be performed. Complete concealment of randomization sequence is warranted.

7.3 Study procedures

See section 3.

7.4 Withdrawal of individual subjects

Subjects can leave the study at any time for any reason if they wish to do so without any consequences. The investigator can decide to withdraw a subject from the study for urgent medical reasons. Subjects will be asked the reason for withdrawal, and they will also be asked for permission to use their data.

7.5 Replacement of individual subjects after withdrawal

No replacement after withdrawal will be done.

7.6 Follow-up of subjects withdrawn from treatment

Patients withdrawn from this RCT will be treated according to the national guideline i.e. will undergo an appendectomy as this is the standard of care. Follow-up will be one month after treatment

7.7 Premature termination of the study

A Data Monitoring Committee (DMC) will monitor this study. The DMC will meet regularly at 6-month intervals, mainly to assess safety. Data review will include proportion of patients with post-appendectomy complications after delayed appendectomy (in case of failure or recurrent appendicitis after initial non-operative treatment).

8. SAFETY REPORTING

8.1 Section 10 WMO event

In accordance to section 10, subsection 4, of the WMO, the sponsor will suspend the study if there is sufficient ground that continuation of the study will jeopardise subject health or safety. The sponsor will notify the accredited METC without undue delay of a temporary halt including the reason for such an action. The study will be suspended pending a further positive decision by the accredited METC. The investigator will take care that all subjects are kept informed.

8.2 AEs, SAEs and SUSARs

8.2.1 Adverse events (AEs)

Adverse events are defined as any undesirable experience occurring to a subject during the study, whether or not considered related to the investigational products. All adverse events reported spontaneously by the subject or observed by the investigator or his staff will be recorded.

8.2.2 Serious adverse events (SAEs)

A serious adverse event is any untoward medical occurrence or effect that at any dose:

- Results in death;
- Is life threatening (at the time of the event);
- Requires hospitalisation or prolongation of existing inpatients' hospitalisation;
- Results in persistent or significant disability or incapacity;
- Is a congenital anomaly or birth defect;
- Any other important medical event that may not result in death, be life threatening, or require hospitalization, may be considered a serious adverse experience when, based upon appropriate medical judgement, the event may jeopardize the subject or may require an intervention to prevent one of the outcomes listed above.

The principal investigator/local-coordinating investigator from each participating hospital needs to contact the main coordinating investigator in case a SAE occurs. The main coordinating investigator will report the SAEs to the sponsor, which in turn will report the SAEs through the web portal *ToetsingOnline* to the accredited METC

that approved the protocol, within 15 days after the sponsor has first knowledge of the serious adverse events. SAEs will also be reported to the DMC.

SAEs that result in death or are life threatening should be reported expedited. The expedited reporting will occur not later than 7 days after the responsible investigator has first knowledge of the adverse event. This is for a preliminary report with another 8 days for completion of the report.

Based upon our previous pilot study the following SAEs do not require immediate reporting:

- Failure of the initial non-operative treatment strategy, leading to delayed appendectomy with an uneventful post-operative course period.
- Recurrent appendicitis after non-operative treatment strategy, leading to delayed appendectomy with an uneventful post-operative course.

A list of the abovementioned SAEs will be submitted through the web portal *ToetsingOnline* to the accredited METC that approved the protocol by line listing with an interval of 3 months.

8.2.3 Suspected unexpected serious adverse reactions (SUSARs)

Adverse reactions are all untoward and unintended responses to an investigational product related to any dose administered.

Unexpected adverse reactions are SUSARs if the following three conditions are met:

1. The event must be serious (see chapter 8.2.2);
2. There must be a certain degree of probability that the event is a harmful and an undesirable reaction to the medicinal product under investigation, regardless of the administered dose;
3. The adverse reaction must be unexpected, that is to say, the nature and severity of the adverse reaction are not in agreement with the product information as recorded in:
   - Summary of Product Characteristics (SPC) for an authorised medicinal product;

The sponsor will report expedited the following SUSARs through the web portal *ToetsingOnline* to the METC:

- SUSARs that have arisen in the clinical trial that was assessed by the METC;

- SUSARs that have arisen in other clinical trials of the same sponsor and with the same medicinal product, and that could have consequences for the safety of the subjects involved in the clinical trial that was assessed by the METC.

The remaining SUSARs are recorded in an overview list (line-listing) that will be submitted once every half year to the METC. This line listing provides an overview of all SUSARs from the study medicine, accompanied by a brief report highlighting the main points of concern.
The expedited reporting of SUSARs through the web portal ToetsingOnline is sufficient as notification to the competent authority.

The sponsor will report expedited all SUSARs to the competent authorities in other Member States, according to the requirements of the Member States.

The expedited reporting will occur not later than 15 days after the sponsor has first knowledge of the adverse reactions. For fatal or life threatening cases the term will be maximal 7 days for a preliminary report with another 8 days for completion of the report.

The principal investigator/local-coordinating investigator from each participating hospital needs to contact the main coordinating investigator in case a SUSAR occurs. The main coordinating investigator will report the SUSARs to the sponsor, which in turn will report the SUSARs through the web portal *ToetsingOnline* to the accredited METC that approved the protocol, within 15 days after the sponsor has first knowledge of the serious adverse events.

8.3 Annual safety report

In addition to the expedited reporting of SUSARs, the sponsor will submit, once a year throughout the clinical trial, a safety report to the accredited METC, competent authority, and competent authorities of the concerned Member States.
This safety report consists of:

- A list of all suspected (unexpected or expected) serious adverse reactions, along with an aggregated summary table of all reported serious adverse reactions, ordered by organ system, per study;
- A report concerning the safety of the subjects, consisting of a complete safety analysis and an evaluation of the balance between the efficacy and the harmfulness of the medicine under investigation.

8.4 Follow-up of adverse events

All AEs will be followed until they have abated, or until a stable situation has been reached. Depending on the event, follow up may require additional tests or medical procedures as

indicated, and/or referral to the general physician or a medical specialist. SAEs need to be reported till end of study within the Netherlands, as defined in the protocol

8.5 Data Monitoring Committee (DMC) (See DMC charter Appendix H)

An independent Data Monitoring Committee will review the accumulating data for safety six-monthly. All members of the DMC will be independent and have no conflict of interest with the sponsor of this study. All potential DMC members will have sight of the protocol/outline before agreeing to join the committee. The main aim of the DMC is to safeguard the interests of trial participants, assess the safety of the interventions during the study, and monitor the overall conduct of this study. The DMC should receive and review the progress and accruing data of this study and provide advice on the conduct of the study to the Trial Steering Committee.

The DMC should inform the Chair of the steering committee and advise premature termination of the study when, in their view:

(i) It becomes evident that patients who have to undergo a delayed appendectomy (in the non-operative treatment strategy have a significant higher risk of complications.

(ii) It becomes clear that the participation rate is too low and the aimed number of patients to be included is not feasible in the scheduled time.

The advice(s) of the DMC will only be sent to the Principal Investigator/steering committee. Should the Principal Investigator/steering committee decide not to fully implement the advice of the DMC, the Principal Investigator/steering committee will send the advice to the reviewing METC, including a note to substantiate why (part of) the advice of the DMC will not be followed.

9. STATISTICAL ANALYSIS

The primary data analysis will be done according to the Intention-To-Treat principle (ITT). However, since the ITT analysis is known to underestimate effects, which can lead to inappropriate rejection of the null-hypothesis in non-inferiority research, a per protocol analysis will be performed as well. We will use logistic and linear regression analysis for binary and continuous outcomes, respectively, to adjust for stratification factors. If necessary, continuous outcomes will be transformed beforehand to obtain a normal distribution. Differences in proportions, Numbers Needed to Treat and absolute or relative differences in continuous outcomes will be presented with their 95% Confidence Intervals, except for the proportion of patients with complications within one year (primary outcome),

for which a one-sided 97.5% CI limit will be given. In a secondary analysis the information recorded during the initial hospital stay in the initial non-operative treatment arm will be analysed in a multi-variate logistic regression model in order to identify potential predictive variables for delayed appendectomy. This will improve future selection of patients that will benefit from non-operative treatment alone.

9.1 Primary study parameter(s)

The difference in proportions of patients experiencing complications will be presented with its one-sided 97.5% Confidence Interval. Logistic regression analysis will be performed to adjust for stratification factors and for potential confounders in case the randomisation process has led to important baseline imbalance.

9.2 Secondary study parameter(s)

We will use logistic and linear regression analysis for binary and continuous outcomes, respectively, to adjust for stratification factors. If necessary, continuous outcomes will be transformed beforehand to obtain a normal distribution. Differences in proportions, Numbers Needed to Treat and absolute or relative differences in continuous outcomes will be presented with their 95% Confidence Intervals.

9.3 Interim analysis

No interim analysis will be performed for efficacy or futility. The DMC will review accumulating data for safety every 6 months as is described in the DMC charter. (Appendix H)

10. ETHICAL CONSIDERATIONS

10.1 Regulation statement

This RCT will be conducted according to the principles of the Declaration of Helsinki (version 2013 Fortaleza Brazil, October 2013) and in accordance with the Medical Research Involving Human Subjects Act (WMO) and ICP-GCP.

10.2 Recruitment and consent

Patients will be recruited at the emergency departments of the participating hospitals. When presenting with symptoms, suggestive for appendicitis, information regarding this study will be provided to the child as well as their legal guardians (age specific information letters). After the patient and his/hers legal guardian have read these documents, the supervising doctor/ investigator / research nurse will give verbal explanation and will answer any questions. When the diagnosis of appendicitis is confirmed radiologically, the child and their legal guardians will be asked for written informed consent, if they want to participate. Due to the emergency setting, time for consideration will be limited. For this reason, information will be provided when the suspicion of appendicitis is there, so that there will be sufficient time to consider their decision. For this same reason the situation could occur that both parents are not present at the time of inclusion. The physician obtaining informed consent must always aim to obtain written consent from both legal guardians. However if this is not possible in the semi acute setting, written consent must be obtained for the study participant if older than 11 years and from at least one parent. The other parent must give its informed consent by telephone which is then documented in the patient file and the written consent must be obtained at a later date.

10.3 Objection by minors or incapacitated subjects

This will be handled according to the national guideline (See appendix I)

10.4 Benefits and risks assessment, group relatedness

Risks of participation:

- Specific non-operative treatment strategy: possible need for delayed operation (10-25%), recurrent appendicitis (10%).
- Both strategies**:** Allergic reaction to antibiotics (<1%), known appendectomy associated complications (5-10%).

Burden of participation:

- Specific non-operative treatment strategy: Extra admission day (in comparison with appendectomy), ultrasonography after 48 hours (extra non-invasive procedure), Blood samples after 24 and 48 hours for determination of C-reactive protein / Leucocytes (2x extra a 1 cc). Blood samples will be obtained through the already placed IV access. In case this is not successful an extra venapunction will be performed.

- Both strategies:
  - Telephone interviews/Email (3x5minutes=15 minutes).

- Filling out questionnaires (1x only QOL, 4x QOL, iMCQ and iPCQ, PSQ-18, net promotor scale). Duration of filling out questionnaires:
  - QOL: 10 minutes (5x10=50 minutes)
  - iMCQ and iPCQ: 10 minutes (3x10=30 minutes)
  - PSQ/Net premotor scale: 5 minutes (4x5=20 minutes)
  - Total time per participant in one year follow up: 100 minutes

2. Benefit of non-operative treatment strategy: Avoidance of surgery (75-90%) and its related early and late morbidity, potential better quality of life.

11. ADMINISTRATIVE ASPECTS, MONITORING AND PUBLICATION

11.1 Handling and storage of data and documents

All data will be handled confidentially, anonymously and in accordance with the Dutch Personal Data Protection Act. Data will be inserted and gathered into an online database provided by Castor (Castor EDC version 4.6; Amsterdam; The Netherlands). Local investigators will insert data. All registration will be monitored and is in line with GCP guidelines. Access to data is limited to the research team (local investigators, coordinating investigator and principal investigator), Inspection for Healthcare and monitors and auditors. The subject identification code list will be in the hands of the coordinating and principal investigator. When necessary, the principal investigator can decide to unlock the subject identification. Data from this study will be stored for a period of at least 15 years.

11.2 Monitoring and Quality Assurance

The Clinical Research Bureau of the VU University Medical Centre will do external monitoring of this study. Prior to start of this study monitoring plan will be set up. Monitoring will be done at least once a year in all participating centres. All centres will be visited at the end of the study. Monitoring will take place with specific attention to informed consent, data monitoring and completeness of case record form.

11.3 Amendments

A ‘substantial amendment’ is defined as an amendment to the terms of the METC application, or to the protocol or any other supporting documentation, that is likely to affect to a significant degree:

- the safety or physical or mental integrity of the subjects of the trial;
  - the scientific value of the trial;
  - the conduct or management of the trial; or

- the quality or safety of any intervention used in the trial.

All substantial amendments will be notified to the METC and to the competent authority. Non-substantial amendments will not be notified to the accredited METC and the competent authority, but will be recorded and filed by the sponsor.

11.4 Annual progress report

The sponsor/investigator will submit a summary of the progress of the trial to the accredited METC once a year. Information will be provided on the date of inclusion of the first subject, numbers of subjects included and numbers of subjects that have completed the trial, serious adverse events/ serious adverse reactions, other problems, and amendments.

11.5 End of study report

The sponsor will notify the accredited METC and the competent authority of the end of the study within a period of 90 days. The end of the study is defined as the last follow-up by telephone at 12 months after discharge for their treatment of acute appendicitis.

In case the study is ended prematurely, the sponsor will notify the accredited METC and the competent authority within 15 days, including the reasons for the premature termination.

Within one year after the end of the study, the investigator/sponsor will submit a final study report with the results of the study, including any publications/abstracts of the study, to the accredited METC and the Competent Authority.

11.6 Public disclosure and publication policy

Results will be disclosed in peer-reviewed, open access, scientific journal. This study is yet registered in a public trial registry (Dutch trial register and on clinicaltrials.gov). Raw data is available on request, after discussion with the Principal Investigator and the DSMB. Standardized forms will be used for data collection. The AMC implemented a SOP for research data management. This guides, amongst others, data stewardship, control on the (software) applications and adherence to the AMC policy for privacy and security. Data storage falls under the central IT-Regime (standardized in AMC). After completion of this trial, the provided data will be used to modify/revise the new written national guidelines for acute appendicitis in children in the Netherlands from which [redacted] is the chairman (appointed by the Netherlands Association of Surgeons (NVvH) and the Order of Medical Specialists (OMS)).

12. STRUCTURED RISK ANALYSIS

For this purpose, the risk assessment tool from the Clinical Research Unit of the AMC was used.

This proposed study is a sequel study based upon our pilot study conducted with the reference number: NL 38141.029.11. This study concluded that initial non-operative treatment of acute simple appendicitis in children aged 7-17 years is safe. Our study protocol included daily blood samples (2cc in total extra) and an additional ultrasonography after 48 hours (non-invasive). These extra procedures are also included in this study protocol in order to reduce the risk of patients in the initial non-operative treatment arm. Antibiotics used in this arm are within the registered indication and therefore the SPC can be used.

Potential risks in participating to this study are:

- Appendectomy (current standard of care): allergic reaction to antibiotics (<1%) and potential post-appendectomy complications (5-10%)
- Antibiotic treatment: allergic reaction to antibiotics (<1%), need for delayed appendectomy (10-25%) and recurrent appendicitis (10%).

To reduce the latter mentioned risk of recurrent appendicitis the additional ultrasound after 48 hours is included. Children with a faecoltih are at higher risk of developing recurrent appendicitis. In case a faecolith is noted, the child will be advised to undergo an appendectomy. There is a risk of needing a delayed appendectomy during the clinical course of the initial non-operative treatment strategy due to the lack of improvement. In order to identify these patients at an early stage, daily blood samples will be obtained, clinical assessment by a physician will take place twice a day, and monitoring of the pain level and vital parameters will be done four times daily. In case of clinical deterioration, the decision will be made to proceed to appendectomy. This protocol has been proven to be safe in the previous pilot study and therefore we believe that risks in this study are reduced to a minimum. To monitor safety during the trial, a DMC will be installed. Moreover due the emergency scope of this trial and the complex nature/unusual care of appendicitis, problems may arise with the participants' rights (especially informed consent procedure for minors). Therefore extensive training will be given to all participating personnel. Moreover an external monitoring will be installed.

Overall we consider the final risk classification of this trial as moderate and due to this several safety issues such as the installation of a DMC and external monitoring will be set up.

13. REFERENCES

1. CBS. Ziekenhuispatiënten; geslacht leeftijd en diagnose 2005. Juli 2009. Available from: http://statline.cbs.nl/StatWeb/publication/default.aspx. Accessed on 13 november 2015
2. Fitz RH. Perforating inflammation of the vermiform appendix. Am J Med Sci 1886;92:321-346
3. Livingstone et al. Disconnect between the incidence of non perforated and perforated appendicitis: Implications for pathophysiology and management. Ann Surg 2007;245:886-892
4. Andersson RE. The natural history and traditional management of appendicitis revisited: spontaneous resolution and predominance of prehospital perforations imply that a correct diagnosis is more than an early diagnosis. World J Surg 2007;31:86-92
5. Narsule et al. Effect of delay in presentation on rate of perforation in children with appendicitis. Am J Emerg Med 2010; in press. Accessible through doi: 10.1016/j.ajem.2010.04.005
6. Maroju et al. Delay in surgery for acute appendicitis. ANZ J Surg 2004;74:773-776
7. Yardeni et al. Delayed versus immediate surgery in acute appendicitis: do we need to operate during the night? J Pediatr Surg 2004;39:464-469
8. Cobben et al. Spontaneously resolving appendicitis etc. Radiology 2000, 215(2): 349-52
9. Williams RF, Blakely ML, Fischer PE, Streck CJ, Dassinger MS, Gupta H, Renaud EJ, Eubanks JW, Huang EY, Hixson SD, Langham MR. Diagnosing ruptured appendicitis preoperatively in pediatric patients. J Am Coll Surg 2009;208:819-825
10. Broker MEE, Lieshout EMM van, Elst M van der, Stassen LPS, Schepers T. Discriminating between simple and perforated appendicitis. J Surg Res 2012;176:79-83
11. Peng YS, Lee HC, Yeung CY, Sheu JC, Wang NL, Tsai YH. Clinical criteria for diagnosing perforated appendix in pediatric patients. Pediatr Emerg Care 2006;22:475-479
12. Oliak D, Yamini D, Udani VM, Lewis RJ, Vargas H, Arnell T, Stamos MJ. Can perforated appendicitis be diagnosed preoperatively based on admission factors. J Gastrointest Surg 2000;4:470-474
13. Gorter et al. A scoring system to predict the severity of appendicitis in children. J Surg Res 2016;200:452-459
14. Eriksson et al. Randomized clinical trial of appendectomy versus antibiotic therapy for acute appendicitis.Br J Surg 1995;82:166-169
17. Styrud et al. Appendectomy versus antibiotic treatment in acute appendicitis. A prospective multicentre randomized controlled trial. World J Surg 2006;30:1033-1037

18. Salminen et al. Antibiotic Therapy vs Appendectomy for Treatment of Uncomplicated Acute Appendicitis: The APPAC Randomized Clinical Trial. JAMA 2015;313(23):2340-8.
19. Hansson et al. Randomized clinical trial of antibiotic therapy versus appendectomy as primary treatment of acute appendicitis in unselected patients. Br J Surg 2009;96:473-481
20. Vons et al. Amoxicillin plus clavulanic acid versus appendicectomy for treatment of acute uncomplicated appendicitis. An open label, non-inferiority, RCT. Lancet 2011;377:1573-1579
21. Mason et al Meta-analysis of randomized trials comparing antibiotic therapy with appendectomy for acute uncomplicated (no abscess or phlegmon) appendicitis. Surg Infect (Larchmt) 2012;13(2):74-84.
22. Liu et al. Use of antibiotics alone for treatment of uncomplicated acute appendicitis: a systematic review and meta analysis. Surgery 2011;150:673-683
23. Varadhan et al. Safety and efficacy of antibiotics compared with appendicectomy for treatment of uncomplicated acute appendicitis: meta-analysis of randomised controlled trials. BMJ 2012;344:e2156
24. Ansaloni et al. Surgery versus conservative antibiotic treatment in acute appendicitis: a systematic review and meta-analysis of RCT. Dig Surg 2011;28:210-221
25. Tanaka et al. Long-term outcomes of operative versus nonoperative treatment for uncomplicated appendicitis. J Pediatr Surg 2015;50:1893-1897
26. Svensson et al. Nonoperative treatment with antibiotics versus surgery for acute non perforated appendicitis in children; a pilot RCT. Ann Surg 2015;261:67-71
27. Armstrong J, et al. Non-operative management of early acute appendicitis in children. Is it safe and effective? J Peadiatr Surg 2014;49:782-785
28. Abes et al. Nonoperative treatment of acute appendicitis in children. J Pediatr Surg 2007;42:1439-1442
29. Hartwich et al. Nonoperative treatment of acute appendicitis in children: a feasibility study. J Pediatr Surg 2016;51:111-116
30. Gorter et al. Initial antibiotic treatment for acute simple appendicitis in children is safe: Short-term results from a multicenter, prospective cohort study. Surgery 2015 May;157(5):916-23.
31. Goldstein B et al. International pediatric sepsis consensus conference: definitions for sepsis and organ dysfunction in pediatrics. Pediatr Crit Care Med 2005;6:2-8
32. NVVH. Richtlijn voor diagnostiek en behandeling van acute appendicitis. Februari 2010. Available from: www.nvgic.nl. Accessed on 1 August 2015
33. Nederlands Kenniscentrum Farmacotherapie bij kinderen. Available through:

http://www.kinderformularium.nl/search/stof.php?id=82

http://www.kinderformularium.nl/search/stof.php?id=78
http://www.kinderformularium.nl/search/stof.php?id=72

32. NVK. Richtlijn Pijnmeting en behandeling van pijn bij kinderen. October 2007. Available from www.pallialine.nl. Accessed on 1 August 2015

Appendix A

SPC Augmentin RVG 29420 / RVG 24815

Appendix B.

SPC Gentamicin RVG 57572

Appendix C.

Sepsis criteria [29]

Table 2. Definitions of systemic inflammatory response syndrome (SIRS), infection, sepsis, severe sepsis, and septic shock

SIRS[a]

The presence of at least two of the following four criteria, **one of which must be abnormal temperature or leukocyte count:**

- Core[b] temperature of >38.5°C or <36°C.
- Tachycardia, defined as a mean heart rate >2 SD above normal for age in the absence of external stimulus, chronic drugs, or painf otherwise unexplained persistent elevation over a 0.5- to 4-hr time period **OR for children <1 yr old: bradycardia, defined as a mea <10th percentile for age in the absence of external vagal stimulus, β-blocker drugs, or congenital heart disease; or otherwise unex persistent depression over a 0.5-hr time period.**
- Mean respiratory rate >2 SD above normal for age or mechanical ventilation for an acute process not related to underlying neuron disease or the receipt of general anesthesia.
- Leukocyte count elevated or depressed for age (not secondary to chemotherapy-induced leukopenia) or >10% immature neutrophi

Infection

A suspected or proven (by positive culture, tissue stain, or polymerase chain reaction test) infection caused by any pathogen OR a clinic associated with a high probability of infection. Evidence of infection includes positive findings on clinical exam, imaging, or laboratory white blood cells in a normally sterile body fluid, perforated viscus, chest radiograph consistent with pneumonia, petechial or purpuric purpura fulminans)

Sepsis

SIRS in the presence of or as a result of suspected or proven infection.

Severe sepsis

Sepsis plus one of the following: **cardiovascular organ dysfunction OR acute respiratory distress syndrome OR two or more other organ dysfunctions. Organ dysfunctions are defined in Table 4.**

Septic shock

Sepsis and cardiovascular organ dysfunction as defined in Table 4.

Modifications from the adult definitions are highlighted in boldface.

[a]See Table 3 for age-specific ranges for physiologic and laboratory variables; [b]core temperature must be measured by rectal, bladder, or catheter probe.

Table 4. Organ dysfunction criteria

Cardiovascular dysfunction

Despite administration of isotonic intravenous fluid bolus ≥40 mL/kg in 1 hr

- Decrease in BP (hypotension) <5th percentile for age or systolic BP <2 SD below normal for age[a]

OR

- Need for vasoactive drug to maintain BP in normal range (dopamine >5 μg/kg/min or dobutamine, epinephrine, or norepinephrine at any do

OR

- Two of the following
  - Unexplained metabolic acidosis: base deficit >5.0 mEq/L
  - Increased arterial lactate >2 times upper limit of normal
  - Oliguria: urine output <0.5 mL/kg/hr
  - Prolonged capillary refill: >5 secs
  - Core to peripheral temperature gap >3°C

Respiratory[b]

- Pao_2/Fio_2 <300 in absence of cyanotic heart disease or preexisting lung disease

OR

- $Paco_2$ >65 torr or 20 mm Hg over baseline $Paco_2$

OR

- Proven need[c] or >50% Fio_2 to maintain saturation ≥92%

OR

- Need for nonelective invasive or noninvasive mechanical ventilation[d]

Neurologic

- Glasgow Coma Score ≤11 (57)

OR

- Acute change in mental status with a decrease in Glasgow Coma Score ≥3 points from abnormal baseline

Hematologic

- Platelet count <80,000/mm³ or a decline of 50% in platelet count from highest value recorded over the past 3 days (for chronic hematology/oncology patients)

OR

- International normalized ratio >2

Renal

- Serum creatinine ≥2 times upper limit of normal for age or 2-fold increase in baseline creatinine

Hepatic

- Total bilirubin ≥4 mg/dL (not applicable for newborn)

OR

- ALT 2 times upper limit of normal for age

BP, blood pressure; ALT, alanine transaminase.

[a]See Table 2; [b]acute respiratory distress syndrome must include a Pao_2/Fio_2 ratio ≤200 mm Hg, bilateral infiltrates, acute onset, and no eviden left heart failure (Refs. 58 and 59). Acute lung injury is defined identically except the Pao_2/Fio_2 ratio must be ≤300 mm Hg; [c]proven need assumes ox requirement was tested by decreasing flow with subsequent increase in flow if required; [d]in postoperative patients, this requirement can be met if the pa has developed an acute inflammatory or infectious process in the lungs that prevents him or her from being extubated.

Appendix D.

Klinische detorioratie

Gebaseerd op de cohort studie, spreken wij een aantal standaard situaties af waarin de behandelend chirurg na initieel non-operatieve behandeling kan overgaan tot een appendectomie.

In alle RCT's die recent zijn gepubliceerd worden er geen criteria genoemd voor de maat clinical deterioration, een belangrijk factor waarom er tijdens toediening van antibiotica toch alsnog gekozen wordt voor een appendectomie.

Op basis van de literatuur zijn wij tot het volgende lijstje gekomen:

1. Diffuse abdominal pain/tenderness/rigidity (25)
2. White blood cell count > 19.4 x 10^9/L; Elevation of the leucocytes after 48 hours (25)
3. Persistent fever (>38.5 degree Celsius) after 24 hours after start of iv antibiotics
4. Persistent tachycardia after 24 hours after start of iv antibiotics
5. Persistent or further elevation of C-reactive protein after 48 hours

Voor de situatie in Nederland worden deze situaties als volgt geoperationaliseerd:

A. Bij follow up aanwijzingen voor een diffuus geprikkelde buik
B. Bij follow up hebben van een leukocytose van >20x10^9 cells/L of een doorstijgende leukocytose na 48 uur na het starten van de intraveneuze antibiotica
C. Het hebben van persisterende koorts (>38,5 graden Celsius) 24 uur na aanvang van de intraveneuze antibiotica
D. Het hebben van een tachycardie (waarde afhankelijk van leeftijd) 24 uur na aanvang van de intraveneuze antibiotica
E. Persisterend of stijgend CRP na 48 uur na aanvang van de intraveneuze antibiotica.
F. Patiënt die niet voldoet aan de ontslagcriteria na 72 uur iv antibiotica.

Het hebben van een of meerdere criteria mag leiden

tot: Noodzaak tot verder beeldvormend onderzoek

Het besluit door een chirurg om over te gaan tot een appendectomie. Dit besluit ligt bij de behandeld chirurg en niet bij de studiegroep/studiecoördinatoren. Indien er wordt besloten tot het verrichten van een appendectomie zal de reden altijd worden genoteerd.

Appendix E.

Ultrasound

Diagnostic signs suggestive for simple appendicitis:

1. Incompressible appendix with a maximal outer diameter enlargement beyond 6mm
2. Periappendiceal hypoechogenic halo associated with wall oedema
3. Wall hyperaemia on colour Doppler
4. Echogenic oedematous mesenteric fat stranding
5. Tenderness of the appendix during examination
6. Local free clear fluid < 0,5 cm

The following signs on ultrasound are suggestive of complex appendicitis i.e. perforation:

a. Appendicolith
b. Loss of echogenic submucosal layer
c. Absent of blood flow in thickened appendiceal wall
d. Excessive free fluid defined as:
    i. The presence of complex fluid collection (cloudy/thick/air bubbles/swirling present)
    ii. More than > 1 cm of clear fluid
e. (Periappendiceal) abscess
    i. Walled off hypoechogenic complex fluid collection with enhanced through-transmission but with internal echogenics (necrotic debris, pus, gas)
f. Bacterial peritonitis
    i. Free fluid collection > 1 cm along peritoneal reflections. Diffuse spread in the abdominal cavity. Inflammatory changes of mesentery and omentum
g. Pneumoperitoneum
    i. Localized extra luminal gas in the right lower quadrant
h. Small bowel obstruction (paralytic)
    i. Multiple dilated loops with fluid

Appendix F.

Predefined discharge criteria

**Predefined Discharge Criteria:**			
**Code**	**Criteria**	**Yes**	**No**
1	***Body temperature < 38.0 degrees Celsius***		
2	***VAS/Comfort scale <4***		
3	***Adequate oral intake***		
4	***Able to mobilize***		
5	***Decreased Leucocytosis***		
6	***Decreased level of C-reactive protein***	*4.*	*5.*
7	***No signs of complex appendicitis on ultrasound***	*6.*	*7.*
8	***Consent of parents for discharge***	*8.*	*9.*

Appendix G.

Scoring system [13]

Determinants	*Adjusted OR (95% CI)*	*p value*	*Awarded points*
**Final model**			
Diffuse abdominal guarding	5.4 (1.4-21.1)	0.01	3
CRP level more than 38 mg/L	4.4 (1.4-14.6)	0.009	2
"Signs indicative of complex appendicitis"	4.2 (1.3-13.5)	0.02	2
>1 day of abdominal pain	3.9 (1.3–12.0)	0.02	2
Temperature > 37.5 degree Celsius	2.6 (0.9–7.7)	0.09	1

Cut-off value:
<4: Simple appendicitis

Appendix H.

DMC charter

Appendix I.
Appendix Codes of conduct minors.

Available through:
http://www.ccmo-online.nl/main.asp?pid=21#ri

Gedragscode bij verzet van minderjarigen die deelnemen aan medisch-wetenschappelijk onderzoek

Inleiding

De Wet Medisch-wetenschappelijk Onderzoek met mensen (WMO) is op 1 december 1999 in werking getreden. In Nederland mag medisch-wetenschappelijk onderzoek met mensen – gezonde proefpersonen en patiënten – uitsluitend worden uitgevoerd als een erkende medisch-ethische toetsingscommissie daarover positief heeft geoordeeld. Deze commissies baseren zich daarbij op criteria die in de wet zijn opgenomen. Bijzondere aandacht schenkt de wet aan het onderzoek met mensen die zelf geen toestemming hiervoor kunnen geven: wilsonbekwame volwassenen en minderjarigen. Het verlenen van toestemming aan het onderzoek met mensen die wilsonbekwaam of anderszins afhankelijk zijn, zoals minderjarigen, gebeurt op grond van het principe "nee-tenzij". De centrale commissie voor medisch-wetenschappelijk onderzoek met mensen (CCMO: Centrale Commissie Mensgebonden Onderzoek) toetst de protocollen van bepaalde vormen van onderzoek met minderjarigen en wilsonbekwame volwassenen. Wanneer hierbij sprake is van niet-therapeutisch medisch-wetenschappelijk onderzoek (dat is onderzoek waarbij de patiënt of gezonde proefpersoon geen direct belang heeft) eist de wet dat de risico's verwaarloosbaar zijn en de eventuele bezwaren minimaal. Bovendien moet dan sprake zijn van onderzoek dat niet dan met medewerking van proefpersonen uit de categorie waartoe de proefpersoon behoort, kan worden verricht (WMO, art. 4, lid 1).
Indien de betrokken proefpersoon zich bij een dergelijk onderzoek verzet tegen een handeling waaraan hij wordt onderworpen of tegen een aan hem opgelegde. gedragswijze, vindt het onderzoek niet plaats met die proefpersoon (WMO, art. 4, lid 2). Bij de behandeling van het wetsontwerp in de Tweede en Eerste Kamer zijn vragen gesteld over artikel 4, lid 2. Wat betekent in de praktijk "verzet" en hoe gaat de onderzoeker daar mee om? De Minister van VWS heeft aan de Kamer toegezegd in overleg met de betrokken beroepsgroepen te komen tot een gedragscode waarin dit wordt uitgewerkt.
De Nederlandse Vereniging voor Kindergeneeskunde (NVK) stelt hierbij gedragsregels vast voor het medisch-wetenschappelijk onderzoek met minderjarigen.

Gedragsregels

1. Elk kind reageert op een eigen wijze bij de diagnostiek en behandeling en ook bij het deelnemen aan wetenschappelijk onderzoek. Een dergelijke reactie wordt mede bepaald door de wijze waarop het kind is voorbereid op de handelingen, de ouder-kind relatie en de arts-patiënt relatie, de kindvriendelijkheid van het milieu waarin de handelingen plaatsvinden etc.
   Het ene kind vindt een prik niet erg (maar zal wel een pijnlijk gezicht tonen), het andere kind vindt het een nare ervaring. Er is een grote variatie in de reactie van het individuele kind, doch in het algemeen is er wel enige relatie met de mate van "invasiviteit" bij het onderzoek. Soms leidt de angst voor het onderzoek of de ingreep tot verzet. Met geduld en begrip zal men het kind zoveel mogelijk informeren en geruststellen en veelal verloopt het onderzoek of de ingreep daarna

zonder problemen.

Bij pasgeborenen en zuigelingen is het verzet moeilijker te beoordelen.
In het algemeen kan men stellen dat er sprake is van verzet, indien het gedrag van het kind duidelijk afwijkt of zich excessiever manifesteert dan men van de betrokkene gewoon is in van de normale dagelijkse routine afwijkende situaties. Onder van de normale dagelijkse routine afwijkende situaties worden ook begrepen diagnostische of therapeutische handelingen.

2. Alvorens toestemming voor deelname aan het onderzoek wordt gevraagd, moeten de ouders van het kind die het gezag uitoefenen dan wel de voogd uitgebreid mondeling en schriftelijk worden geïnformeerd.
   In het overleg met de ouders dient aan de orde te komen wat de onderzoekshandelingen zullen zijn en wordt een inschatting gemaakt over de te verwachten reactie bij het individuele kind. Eventueel verzet van het kind en welk gedrag als verzet zal worden aangemerkt wordt met de ouders van het kind besproken. Hierbij wordt aangegeven wat de gedragslijn is in geval van verzet van het kind.
   De toestemming van de ouders dient tevens overeenstemming te omvatten over de gedragslijn in geval van verzet van het kind.

3. In de toestemmingsverklaring van de ouders dient te worden vastgelegd dat wanneer er sprake is van verzet van het kind bij het onderzoek, toestemming voor verdere deelname aan het onderzoek komt te vervallen.

4. In geval van twijfel over het in beginsel deelnemen bij het starten van het onderzoek kan een proefparticipatie overeengekomen worden.

5. Bij deelname aan het onderzoek moet ter plaatse bij voortduring het gedrag van het kind beoordeeld worden en de afweging gemaakt worden of het gedrag zich nog binnen de grenzen beweegt van wat men van het betrokken kind gewoon is waar te nemen in van de normale dagelijkse routine afwijkende situaties.
   Gaat het gedrag hierbuiten, dan is sprake van verzet in de zin van de WMO.

6. Bij de beoordeling van het gedrag van het kind zijn de ouders, de onderzoeker(s) en eventueel een gedragswetenschapper betrokken. Beoordeling van verzet vindt niet eenmalig plaats, maar blijft een continue aandachtspunt tijdens alle fasen van het onderzoek.

7. Tijdens alle fasen van het onderzoek kunnen de ouders hun toestemming intrekken. In geval van verzet dient het onderzoek bij het betreffende kind gestaakt te worden.

8. Uitgangspunt bij wetenschappelijk onderzoek met kinderen is de belasting tot een minimum te beperken (de wet spreekt bij niet-therapeutisch onderzoek van te verwaarlozen belasting). Wetenschappelijk onderzoek vindt onder meer plaats door het onderzoek te combineren met noodzakelijk diagnostisch onderzoek.
   Wanneer invasief onderzoek plaats vindt in de vorm van een vingerprik of venapunctie zal men dit bij voorkeur combineren met een voor diagnostiek of behandeling noodzakelijke bloedafname. Zo mogelijk zal men gebruik maken van een langere tijd aanwezige naald of lijn, zodat het aantal "prikken" tot een minimum beperkt blijft.
   Verder wordt het ongemak beperkt door het gebruik van pleisters met een

lokaal anaestheticum. Een en ander wordt aangegeven in het onderzoeksprotocol en de informatie voor de ouders en de proefpersoon.

9. In het onderzoeksverslag, respectievelijk het medisch verslag (status), wordt het volgende vastgelegd:
   a. de bevindingen van een eventuele proefparticipatie;
   b. de toestemming van de ouders die het gezag uitoefenen, dan wel de voogd, inclusief de gedragslijn bij verzet;
   c. het verloop van de deelname aan het onderzoek, met de vermelding of er al of niet verzet plaats vindt;
   d. toetsing van het gedrag aan het verzetsgedrag, zoals hierboven beschreven;
   e. de namen van de beoordelaars van het verzetsgedrag, zoals hierboven beschreven;
   f. de beoordeling van het verzetsgedrag gedurende het onderzoek;
   g. de wijze waarop de belasting voor de proefpersoon tot een minimum wordt beperkt.

   In geval van medisch-wetenschappelijk onderzoek met minderjarigen wordt in het onderzoeksprotocol aangegeven dat men zich zal houden aan de door de NVK vastgestelde gedragscode hoe te handelen bij verzet van de proefpersonen tijdens het onderzoek.

10. Deze gedragscode zal na een periode van twee jaar na publicatie worden geëvalueerd in overleg met het onderzoeksveld en dan zonodig worden aangepast.

Deze gedragscode is in de vergadering van het Bestuur van de Nederlandse Vereniging voor Kindergeneeskunde (NVK) op 21 mei 2001 aanvaard, wordt gepubliceerd in Nieuwsbrief NVK nr. 3, juni 2001 en is aangeboden aan het Tijdschrift voor Kindergeneeskunde.

Appendix J.

Appendix Risk-assessment CRU.
