# Supplementary material for: Non-operative treatment strategy versus surgery for children with simple appendicitis: non-inferiority randomised controlled trial
Source: BMJ Med. 2026 May 13;5(1):e002466. doi: 10.1136/bmjmed-2025-002466 (PMC13182491; doi:10.1136/bmjmed-2025-002466)
Supplement: online supplemental file 2 [file bmjmed-5-1-s002.pdf]

Statistical analysis plan (SAP)

APAC trial

Version 1.0 - 18 sep 2024

Section 1a. Title.

What is the title of the statistical analysis plan?

Statistical analysis plan for the APAC trial: Antibiotics versus Primary Appendectomy in Children with simple appendicitis, a multicentre randomized controlled trial in order to evaluate the effectiveness of an initial non-operative treatment strategy compared to appendectomy as treatment for children with acute simple appendicitis.

Section 1c. Revision history of the statistical analysis plan.				
**What versions of the statistical analysis plan have been approved and filed and what was the reason for producing each version?**				
**Updated statistical analysis plan version**	**Protocol version**	**Section number(s) changed**	**Description of and reason for changes**	**Date of approval**
1.0	2.2	N/A	N/A	18 sep 2024
…	…	…	…	…

Section 1d. Administrative Information.

1.1. What is the trial registration number?

This study is registered in the Netherlands Trial Register under reference NL5822 and in clinical trials.gov under reference NCT02848820.

1.2. What is the planned period of observation?

The first patient was included on January 13th 2017 and the expected date of completion of follow-up of the last included patient is October 19th 2024.

1.3. What is the date and version number of the current statistical analysis plan?

This statistical analysis plan is version 1.0 dated 31-07-2024

1.4. What is the date, version number and reference number of the protocol used when writing this statistical analysis plan?

This statistical analysis plan is based on the protocol with reference number NL56792.018.16 version 2.2 and dated 20-06-2018

Section 2. Introduction.

2.1. What is the background and rationale for the study?

Initial non-operative treatment of acute simple appendicitis has been investigated in both the adult and the paediatric population. In the adult population, six RCTs showed that an appendectomy could be avoided in 40-76% of the patients at the end of the follow-up period of 12 months. Despite the fact that some patients need to undergo a delayed appendectomy, it has been demonstrated in systematic reviews that non-operative treatment strategy is associated with a significant reduction in complications, faster recovery and return to work, shorter pain duration and less analgesic medication consumption. In children, short-term success rates of this strategy (including our own pilot cohort study) are between the 83-92%. Long-term results (one year follow-up) are available from two studies; 62-75% did not require an appendectomy. No large RCTs have yet been conducted in the paediatric population. It is therefore essential to generate high quality empirical evidence regarding this strategy in this subset of patients.

2.2. What are the objectives of the study?

Primary objective is to investigate the difference in proportion of children experiencing complications within one year between the initial non-operative treatment strategy and direct appendectomy strategy for acute simple appendicitis in children aged 7-17 years.

Secondary this study will investigate:
• The difference in total numbers of days absent from school, sporting events or social events (child) within one year between the initial non-operative treatment strategy and direct appendectomy strategy for acute simple appendicitis in children aged 7-17 years. Days absent counted are those days associated with treatment for acute appendicitis or treatment related complications or recurrent abdominal pain.
• The difference in total numbers of days absent from work (parents) within one year between the initial non-operative treatment strategy and direct appendectomy strategy for acute simple appendicitis in children aged 7-17 years. Days absent counted are those days associated with treatment for acute appendicitis or treatment related complications or recurrent abdominal pain.
• The difference in level of pain measured by the Visual Analogue Scale during the first seven days of treatment between the initial non-operative treatment strategy and direct appendectomy strategy for acute simple appendicitis in children aged 7-17 years.
• The difference in pain medication utilization during the first seven days of treatment between the initial non-operative treatment strategy and direct appendectomy strategy for acute simple appendicitis in children aged 7-17 years.
• The proportion of patients experiencing early failure and recurrent appendicitis after initial non-operative treatment strategy for acute simple appendicitis in children aged 7-17 years.
• The proportion of patients not having to undergo an appendectomy after initial non-operative treatment strategy for acute simple appendicitis in children aged 7-17 years at one month, six months and one year follow up.
• The difference in quality of life during one year between the initial non-operative treatment strategy and direct appendectomy strategy for acute simple appendicitis in children aged 7-17 years.
• The difference in medical, non-medical and indirect costs during one year between the initial non-operative treatment strategy and direct appendectomy strategy for acute simple appendicitis in children aged 7-17 years.
• The difference in patient satisfaction during one year between the initial non-operative treatment strategy and direct appendectomy strategy for acute simple appendicitis in children aged 7-17 years.

Section 3. Study Methods.

3.1. What is the study design?

The study is designed as a unblinded multicentre randomized controlled non-inferiority trial with a 1:1 block randomization stratified by hospital.

3.2. Will randomization be performed in this study?

A total of 302 patients will be randomized to either initial non-operative treatment strategy or immediate operative treatment strategy, using a computer/internet based randomization program provided by Castor software (Castor EDC version 4.6; Amsterdam; The Netherlands). A 1:1 variable block randomization stratified by hospital (total of 16 hospitals) will be performed. Complete concealment of randomization sequence is warranted. Patients and treating physicians are not blinded to the treatment allocation, as this is inherently impossible.

3.3. How was the sample size calculated?

A non-inferiority design will be used based upon evidence in the literature that initial non-operative treatment of appendicitis has many potential secondary advantages. It would be sufficient if this trial demonstrates that the outcome in terms of complications of initial non-operative treatment strategy is not worse than the immediate appendectomy group. Past studies demonstrated that the overall frequency of post-operative complications is approximately 10%, meaning that 90% will be successfully treated without complications. Initial non-operative is estimated to reduce the complication rate with approximately 50%, meaning that 95% will be successfully treated without complications. Using a 1-sided alpha of 2.5%, we need about 150 patients per group to achieve 90% power to exclude a difference in favour of the usual care group of more than 5%. Although in our pilot the drop-out rate in one year was only 2%, we initially took into account a drop-out rate of 10%. Therefore, the number of patients to be included was 334. However, during the study (inclusion phase) only one patient dropped out of the study. Therefore we decided to reduce our sample size to 302 patients.

3.4. What is the hypothesis testing framework for this study?

The APAC trial uses a non-inferiority hypothesis framework for our primary outcome.

3.5. Will interim analyses be performed in this study?

No interim analyses will be performed and there are no statistical or clinical guidelines for stopping the study early.

3.6. When will the final statistical analysis of the study data be performed?

The statistical analysis of all outcomes (both primary and secondary) will be performed after follow-up of the last patient is completed (expected October 2024).

**3.7. At which time points are the outcomes measured and which “windows” are allowed?**
Outcomes will be measured at several time points, including<br>discharge, 7 days [Range 5-9 days after discharge]<br>one month [Range 2-6 weeks after discharge]<br>6 months [Range 5-7 months after discharge]<br>12 months [Range 11-13 months after discharge]

Section 4. Statistical Principles.

4.1. Which level or levels of statistical significance will be used in the study?

When analysing the primary outcome (complications) initial non-operative treatment will be viewed as non-inferior to direct operative treatment if the associated one-sided p-value is larger than 0.025. Secondary outcomes will be viewed as significantly different between initial non-operative treatment and direct operative treatment if the associated two-sided p-values are less than 0.05.

4.2. Will the analysis adjust for multiplicity of statistical testing to ensure control of type I error rate?

As there is one primary outcome measured at a single time point in this study, the analysis will not adjust for multiplicity of statistical testing.

4.3. Which confidence intervals will be reported?

The difference in proportions of patients experiencing complications (primary outcome) will be presented with its one-sided 97.5% Confidence Interval.

For our secondary outcomes differences in proportions, Numbers Needed to Treat and absolute or relative differences in continuous outcomes will be presented with their 95% Confidence Intervals.

4.4. How is compliance defined and assessed?

N/A

4.5. How will compliance be presented?

N/A

4.6. What are defined as protocol deviations in this study?

Situations marked as protocol deviation

1. Switch to antibiotics after randomization because of preference of parents or surgeon.
2. Switch to appendectomy because of preference of parents during or after primary admission without there being any medical grounds.

Situations <u>not</u> marked as a protocol deviation

1. Switch to appendectomy after meeting the criteria for clinical deterioration, signs of complicated appendicitis on the second ultrasound or not meeting the discharge criteria after 3 days of IV antibiotics.

2. Any appendectomy after primary admission, **<u>except</u>** when based on persistent desire of parents without there being any medical grounds.

4.7. How will protocol deviations be presented in the reporting of this study?

All protocol deviations are line listed for each treatment group. The number and percentage of patients experiencing any protocol deviation will be presented in a table in the final report.

4.8. Which analysis populations will be defined?

The primary data analysis will be done according to the Intention-To-Treat principle (ITT). This population includes all randomized patients, regardless of protocol deviations. These protocol deviations include switch to appendectomy after randomization for antibiotics because of parents' or surgeon's preference. Furthermore, switch to appendectomy because of preference of parents during or after primary admission without there being any medical grounds, is indicated as a protocol deviation.

However, since the ITT analysis is known to underestimate effects, which can lead to inappropriate rejection of the null-hypothesis in non-inferiority research, a per protocol analysis will be performed as well. This analysis only includes the patients who completed the treatment originally allocated, thereby excluding the patients that deviated from the protocol.

Section 5. Study populations.

5.1. Which data were collected from participants, who were screened for eligibility for inclusion in the study, and how these data will be presented in study reports?

Screening data will be reported as the number of patients screened for eligibility, the number of patients that fulfilled the inclusion criteria and the number of patients that were approached for informed consent. Due to privacy concerns, no personal data were collected from the patients that were not included in the study.

5.2. What are the inclusion and exclusion criteria for the study?

Inclusion criteria
In order to be eligible to participate in this study, a subject must meet all of the following criteria:
• Age 7-17 years, inclusive
• Radiologically confirmed simple appendicitis, defined as:
    a. Clinical findings:
        i. Unwell, but not generally ill
        ii. Localized tenderness in the right iliac fossa region
        iii. Normal/hyperactive bowel sounds
        iv. No guarding
        v. No mass palpable
    b. Ultrasonography:
        i. Incompressible appendix with an outer diameter of ≥6 mm
        ii. Hyperaemia within the appendiceal wall
        iii. Without faecolith
        iv. Infiltration of surrounding fat
        v. No signs of perforation
        vi. No signs of intra-abdominal abscess/phlegmon

Exclusion criteria
A potential subject who meets any of the following criteria will be excluded from participation in this study:
1. Patients with severe general illness at time of presentation:
    a. Generalized peritonitis defined as:
    Diffuse inflammation of the peritoneum with clinical signs consisting of increasing abdominal pain, generalized tenderness, diffuse abdominal rigidity, sinus tachycardia, and signs of paralytic ileus.
    b. Severe sepsis or septic shock, as defined by the international paediatric sepsis consensus conference
    c. Signs of complex appendicitis. For this our own developed clinical prediction rule will be used.
2. Children with a faecolith on ultrasonography
3. Patients with serious associated conditions or malformations such as:
    a. Congenital or acquired cardiac or pulmonary disease with significant hemodynamic consequences
    b. Immunodeficiency
    c. Malignancy
    d. Homozygous sickle cell disease
    e. Metabolic disorders
4. Patients with documented type 1 allergy to the antibiotics used
5. Patients who have been treated for acute simple appendicitis non-operatively in the past year
6. Patients with a suspicion of an underlying malignancy based upon clinical and radiological evaluation

5.3. Which information will be presented in the flow chart for this study?

The flow of participants will be summarized and presented according to the consolidated standards of reporting trials (CONSORT) method. The flow diagram is presented in the appendix of this statistical analysis plan.

5.4. What is the expected level of, timing of and reasons for withdrawal from the intervention and/or from follow-up and how will this be presented in the study reports?

We expected that 10% of included patients would be lost during our follow-up period of one year. This was taken into account in our sample size calculation and we therefore intended to include 33 additional patients in order to achieve sufficient power. It was expected that these 10% of patients would be lost to follow-up during the one year follow-up period. This 10% also takes into account those patients that will withdraw their consent due to preference for any of the treatment strategies, as described in the section of protocol deviations. Patients lost during follow-up (including number of weeks spent in follow-up) will be reported for each treatment arm separately.

5.5. Which baseline characteristics of participants will be presented?

<u>Baseline demographics</u>

- Age in completed years and months at randomization (continuous variable)
- Gender (categorical variable)
- Height (continuous variable)
- Weight (continuous variable)
- Body Mass Index (continuous variable)
- Allergies to medication (categorical variable)
- Past relevant illnesses (categorical variable)

<u>Baseline presentation</u>

- Number of days of abdominal pain (continuous variable)
- Fever measured at home (continuous variable)
- Anorexia at presentation (categorical variable)
- Nausea at presentation (categorical variable)
- Vomiting at presentation (categorical variable)
- General appearance (categorical variable)
- Abdomen painful during palpation (categorical variable)
- Location of abdominal pain (categorical variable)
- Pulse rate at presentation (continuous variable)
- Temperature at presentation (continuous variable)
- Leukocytes and CRP at presentation (continuous variable)
- VAS score at presentation (continuous variable)

Baseline characteristics will be presented according to the table that is presented in the appendix of this statistical analysis plan.

5.6. How will the baseline characteristics be summarized?

Continuous, normally distributed baseline variables will be presented as mean and standard deviation per allocated group. Continuous, non-normally distributed variables will be presented as median and interquartile range. Categorical baseline variables will be presented as number and percentage in each category. As this is a randomized controlled trial, baseline characteristics will not be tested for differences.

Section 6. Analysis.

6.1. How are the outcomes of this study defined?

Primary endpoint
The proportion of patients experiencing complications within one year-follow up. An independent adjudication committee will review all complications/adverse events reported in the trial, to assess their relation with the treatment, as well as the length of hospital stay (secondary outcome measure). Review of the length of hospital stay is necessary to assess their relation with the treatment or potential complications.
Complications are defined as:
• Allergic reaction to antibiotics administered
• Need for other surgical or radiological intervention other than appendectomy but related to appendicitis
• Re-admission for an indication other than recurrent appendicitis
• Complications associated with appendectomy:
  - Superficial Site infection
  - Intra-abdominal abscess
  - Stump leakage/stump appendicitis
  - Secondary Bowel Obstruction
  - Pneumonia
  - Anaesthesia Related complications
  - Hernia cicatricalis
  - Re-admission

Secondary study parameters/endpoints
Number of days absent from school, social or sport events (patient-level)
Number of days absent from work (parents-level)
Total number of extra visits (not the already scheduled ones) to the outpatient clinic, general practitioner's office or emergency department for abdominal pain.
Total length of hospital stay during the follow-up period for strategy related treatment or complications
Level of pain (measured by the validated Visual Analogue Scale)
Pain medication utilization during the first seven days after admission
Proportion of patients with missed diagnosis of complex appendicitis with risk of peritonitis
Proportion of patients not having to undergone appendectomy
Proportion of patients experiencing recurrent appendicitis within one-year follow-up.
Recurrent appendicitis is defined as those patients with a clinical and radiological high suspicion of recurrent appendicitis who undergo an appendectomy and histopathological examination confirms the diagnosis of recurrent appendicitis
Proportion of patients experiencing early failure of initial non-operative treatment.
Early failure is defined as all patients that undergo an appendectomy during the antibiotic course (iv or oral) due to persistent complaints, clinical deterioration or faecolith.
Proportion of patients that undergo interval appendectomy.
Interval appendectomy is defined as those patients that undergo an appendectomy with a clinical and radiological low suspicion of recurrent appendicitis. Histopathological examination shows no signs of recurrent appendicitis.
Proportion of patients experiencing complications after: discharge, seven days, one month and six months after treatment.
Quality of life measured by the validated CHQ-CF87, EQ-5d-Youth, EQ-5d-Proxy questionnaire.
Medical, non-medical and indirect costs at one year follow up of the treatment strategy measured by the iMCQ and iPCQ which where adapted for use in children and parents, plus gathered actual health care cost.
Patient satisfaction measured by the NET PROMOTOR SCORE and validated Patient Satisfaction Questionnaire (PSQ)18.
Factors influencing implementability

Time of measurement:
- Discharge

- Seven days
- One, six and twelve months

6.2. Will any calculations or transformations be used to derive any outcome from the original data?

For level of pain we will calculate an AUC for the VAS scores on day 1, day 2 and day 7

If necessary, continuous outcomes will be transformed beforehand to obtain a normal distribution. No additional calculations or transformations will be performed

6.3. What analysis method will be used and how the treatment effects will be presented?

The primary outcome will be tested for non-superiority using a chi-square test, one-sided 97.5% CI limit. Both a Per-protocol and ITT will be performed.

Secondary outcomes will be tested for normal distribution. Normally distributed continuous outcomes will be compared using an unpaired T-test.

Comparison of AUC outcomes will be performed using MedCalc (method by Hanley & McNeil (1982 & 1983). Dichotomous outcomes will be compared using a chi-square test, and two sided 95% CIs will be provided of differences in proportions.

Cost-effectiveness analysis

In this study, we will conduct a cost-effectiveness analysis for the proportion of patients with a complication and a cost–utility analysis for Quality of Life (QoL) and Quality-Adjusted Life Years (QALYs)

Costs will be assessed from the societal perspective, integrating healthcare costs and societal costs (loss of productivity). Total (integrated) costs, consisting of direct medical costs, indirect medical costs and indirect costs, will be evaluated for each treatment strategy. The Health and Labor Questionnaire (HLQ) will be used to measure the direct and indirect costs of health care utilization, and days absent from work and school by both the parents and children. Costs will be calculated using the answers from the HLQ. In addition, secondary data will be gathered from the patients' medical chart and financial information system from a representing sample of the participating hospitals including academic and peripheral hospitals.

Variables gathered will be:

- Health care related costs:
    - admission days
    - costs of diagnosis (biochemical testing, imaging studies)
    - costs of monitoring (biochemical testing, imaging studies)
    - costs of treatment (appendectomy / antibiotics) and secondary interventions
    - costs associated with delayed appendectomy (admission days, diagnosis, treatment)
    - costs associated with post-appendectomy complications (admission days, diagnosis, treatment)
    - costs of out-patient follow up
    - costs of extra emergency department visit due to appendicitis related complaints
    - costs of extra general practitioners visit due to appendicitis related complaints
    - costs of physiotherapist visits
    - costs of psychiatrist/psychologist visits
    - costs of occupational therapist visits
    - costs of dietician visits

- domestic help expenses
- travel expenses (including public transport, car, ambulance)

- Societal costs:
  - absenteeism from work
  - absenteeism from voluntary work

Adjustment for inflation to the year 2022 will be made using the price-index-indices as provided by statline.cbs.nl.

Outcome analysis

Total (integrated) costs consist of direct- and indirect medical costs plus indirect costs. In the cost-effectiveness analysis, we will compare the costs and the proportion of a complication between the groups. Results will be expressed as the incremental cost-effectiveness ratio (ICER), defined as the difference in costs divided by the difference in effectiveness. We will conduct bootstrapping to quantify the uncertainty around costs and effects and visualize this in a cost-effectiveness plane. If bootstrap results cross quadrants of the cost-effectiveness plane, the uncertainty surrounding the ICER is not easily expressed as identical numerical values can have very different interpretations. Thus, we will express the uncertainty around the ICER in a cost-effectiveness acceptability curve (CEAC).

In the cost–utility analyses, we will compare the costs and the QALYs. The ICER will be evaluated against a threshold of €20,000 per QALY. QALYs will be calculated using the EQ-5D youth and EQ-5D-Proxy questionnaires, extrapolating between measurement timings. As acute appendicitis is an acute disease, disutility might be short-term in our study. Therefore, QALY's will be transformed to quality-adjusted life months to aid interpretation of numerical values

Sensitivity analyses

Aside from the base case analysis described so far, we will consider several alternative scenarios and address them in sensitivity analyses to see the difference from the base case.

Budget impact analysis

General considerations

Budget impact analysis (BIA) will be performed from the budget holders' perspective, which is the healthcare insurance company. Time-frame will be 5 years as we expect, despite maximum effort, implementation needs some time. Data will be displayed each year taking into account the anticipated market penetration/implementation of the new identified optimal strategies and de-implementation of the current ones. Aim is to predict the effects on budgets after implementation of these new strategies from the stakeholders' perspective (i.e., healthcare professionals, patients and parents, and insurance companies).

6.4. Will any assumptions for statistical methods be checked?

Normality will be assessed by visual inspection of histograms and q-q plots. If necessary, continuous outcomes will be transformed beforehand to obtain a normal distribution.

6.5. Will sensitivity analyses be performed?

In case of substantial missing data (see 6.7) sensitivity analyses will be performed by best case and worst case scenario imputation, both results will be reported.

6.6. Will subgroup analyses be performed?

No subgroup analyses will be performed.

6.7. How will missing data be reported in the study reports and handled in the statistical analysis?

For all outcomes separately the percentage of missing data will be calculated and reported.

In case **less than 10%** of the outcome data is missing, this will be classified as **non-substantial,** and **no additional sensitivity analysis** will be performed.

In case **more than 10%** of the outcome data is missing, this will be classified as **substantial,** and **an additional sensitivity analysis** will be performed as described below 6.5.

We will not perform multiple imputations to correct for missing data.

6.8. Will additional analyses on the primary or secondary outcomes be performed?

N/A

6.9. How will harms be reported?

The number and percentage of patients experiencing any adverse events will be presented separately for each treatment group.

6.10. Which statistical software will be used to carry out the statistical analyses?

IBM SPSS Statistics, Version 28.0 (IBM Corp, Armonk, NY)
MedCalc - version 22.023.

Section 7. References to literature, standard operating procedures and reporting guidelines.

7.1. Are non-standard statistical procedures to be used, which have not been described in sufficient depth in the previous sections?

N/A

7.2. What is the title, date and version number of the current data management plan?

The current data management plan (Datamanagement_APAC_trial_v2.0_17mei2018), version number 2.0, is dated 17-05-2018 and is stored in both the trial master file and filed at L:\basic\divc\Onderzoek Appendicitis\APAC back-up 17aug2022\Datamanagement.

7.3. What is the title, date and version number of the current data validation and derivation plan?

See section 7.2

7.4. Where is the study master file stored?

The trial master file is stored at the Amsterdam UMC, location AMC and at the location L:\basic\divc\Onderzoek Appendicitis\APAC back-up 17aug2022

7.5. Where are the syntax files for data extraction, manipulation and preparation and statistical analysis stored?

The syntax files for data extraction and statistical analysis will be stored at the location L:\basic\divc\Onderzoek Appendicitis\APAC back-up 17aug2022

7.6. Which standard operating procedures will be adhered to when using and analysing data from this study?

For the APAC trial, researchers will adhere to the standard operating procedure AMC RDM001 Research data management, as stated in this section (question 7.2).

7.7. Which reporting guidelines will be adhered to when reporting on this study?

We will adhere to the Consolidated Standards Of Reporting Trials (CONSORT) guidelines.

Appendix. Additional Tables, Figures and Documents.

Figure 1. The flow chart of patients enrolled in the APAC trial.

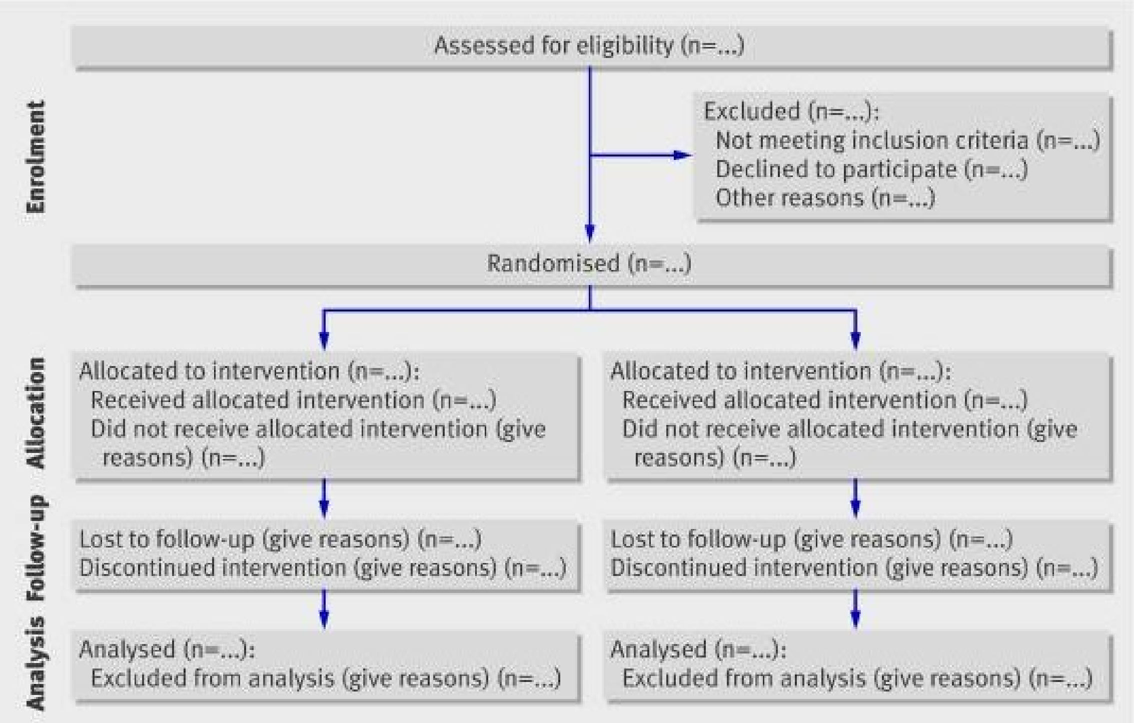

Table 1. The baseline characteristics of patients randomized in the APAC trial

	Non-operative treatment (n=	Appendectomy (n =
Age (y)	Mean (st dev)/Median (IQR)	Mean (st dev)/Median (IQR)
Gender (male)	N (% of total)	N (% of total)
Height (cm)	Mean (st dev)/Median (IQR)	Mean (st dev)/Median (IQR)
Weight (kg)	Mean (st dev)/Median (IQR)	Mean (st dev)/Median (IQR)
Body Mass Index	Mean (st dev)/Median (IQR)	Mean (st dev)/Median (IQR)
Allergies to medication	N (% of total)	N (% of total)
Past relevant ilnesses	N (% of total)	N (% of total)
Number of days abdominal pain	Median (IQR)	Median (IQR)
Fever	N (% of total)	N (% of total)
Anorexia	N (% of total)	N (% of total)
Nausea	N (% of total)	N (% of total)
Vomiting	N (% of total)	N (% of total)
General appearance (ill)	N (% of total)	N (% of total)
Abdomen painful during palpation	N (% of total)	N (% of total)
Location of abdominal pain (RLQ)	N (% of total)	N (% of total)
Pulse (Bpm)	Mean (st dev)/Median (IQR)	Mean (st dev)/Median (IQR)
Temperature (degrees Celsius)	Mean (st dev)/Median (IQR)	Mean (st dev)/Median (IQR)
Leukocytes (10E9/L)	Mean (st dev)/Median (IQR)	Mean (st dev)/Median (IQR)
CRP (mg/l)	Mean (st dev)/Median (IQR)	Mean (st dev)/Median (IQR)
VAS pain score	Mean (st dev)/Median (IQR)	Mean (st dev)/Median (IQR)

Table 2. Primary and secondary outcome measures.

	Non-operative treatment (n=	Missing data (n/% of total)	Appendectomy (n =	Missing data (n/% of total)	Difference (95% CI)
Primary outcome					
Proportion of complications at 1y	N (% of total)		N (% of total)		*one sided 97.5% CI
Secondary outcomes					
Proportion of complications at discharge	N (% of total)		N (% of total)		
Proportion of complications at 7d	N (% of total)		N (% of total)		
Proportion of complications at 1m	N (% of total)		N (% of total)		
Proportion of complications at 6m	N (% of total)		N (% of total)		
Initial length of hospital stay (d)	median (IQR)		median (IQR)		
Total length of hospital stay (d)	median (IQR)		median (IQR)		
Level of pain during the first seven days (VAS score)	Mean (st dev)/ median (IQR)		Mean (st dev)/ median (IQR)		
Paracetamol utilization during the first seven days (days)	Mean (st dev)/ median (IQR)		Mean (st dev)/ median (IQR)		
NSAID utilization during the first seven days (days)	Mean (st dev)/ median (IQR)		Mean (st dev)/ median (IQR)		
Morphine utilization during the first seven days (days)	Mean (st dev)/ median (IQR)		Mean (st dev)/ median (IQR)		
Number of days absent from school, social or sport events (at 1y)	median (IQR)		median (IQR)		
Number of days absent from work (at 1 y)	median (IQR)		median (IQR)		

Total number of extra visits (at 1y)	median (IQR)		median (IQR)		
Missed diagnosis of complex appendicitis (at 1y)	N (% of total)		N (% of total)		
Quality of life at 7d	Mean (st dev)/ median (IQR)		Mean (st dev)/ median (IQR)		
Quality of life at 1m	Mean (st dev)/ median (IQR)		Mean (st dev)/ median (IQR)		
Quality of life at 6m	Mean (st dev)/ median (IQR)		Mean (st dev)/ median (IQR)		
Quality of life at 1y	Mean (st dev)/ median (IQR)		Mean (st dev)/ median (IQR)		
Patient satisfaction at 7d	Mean (st dev)/ median (IQR)		Mean (st dev)/ median (IQR)		
Patient satisfaction at 1m	Mean (st dev)/ median (IQR)		Mean (st dev)/ median (IQR)		
Patient satisfaction at 6m	Mean (st dev)/ median (IQR)		Mean (st dev)/ median (IQR)		
Patient satisfaction at 1y	Mean (st dev)/ median (IQR)		Mean (st dev)/ median (IQR)		
Medical cost	Mean (st dev)/ median (IQR)		Mean (st dev)/ median (IQR)		
Non-medical cost	Mean (st dev)/ median (IQR)		Mean (st dev)/ median (IQR)		
Indirect cost	Mean (st dev)/ median (IQR)		Mean (st dev)/ median (IQR)		
Secondary outcomes for non-operative treatment					
No appendectomy (at 1y)	N (% of total)				
Recurrent appendicitis (at 1y)	N (% of total)				
Early failure of initial non-operative treatment during first seven days	N (% of total)				
Interval appendectomy (at 1y)	N (% of total)				
