# Supplementary material for: Non-operative treatment strategy versus surgery for children with simple appendicitis: non-inferiority randomised controlled trial
Source: BMJ Med. 2026 May 13;5(1):e002466. doi: 10.1136/bmjmed-2025-002466 (PMC13182491; doi:10.1136/bmjmed-2025-002466)
Supplement: online supplemental file 3 [file bmjmed-5-1-s003.pdf]

Supplementary tables

Table S1. Predefined discharge criteria

All criteria have to be met to allow patients to be discharged.

**Predefined discharge criteria (equal for both interventions**	
1.	Body temperature <38°C
2.	NRS<4
3.	Adequate oral intake
4.	Able to mobilize
5.	Consent of parents for discharge
**Predefined discharge criteria only for non-operative management**	
6.	Decreased leucocytosis
7.	Decreased C-reactive protein
8.	No signs of complex appendicitis on second ultrasound

Table S2. CHQ-87

**Child Health Questionnaire-87 Domains** (mean ± SD)<br><br>Scores are reported on a scale from 0-100	**Appendectomy** *(n=151)*	**Missing data** (n/% of total)	**NOT strategy** *(n=151)*	**Missing data** (n/% of total)	**Mean difference** (95%CI)	**p-value**
Global Health						
Baseline	80.6 ± 20.4	80 (53.0)	75.2 ± 23.6	87 (57.6)	-5.4(-13.0 to 2.2)	0.221
7 days	78.8 ± 18.2	67 (44.4)	81.4 ± 15.9	79 (52.3)	2.6(-2.8 to 8.0)	0.460
1 month	81.6 ± 18.3	51 (33.8)	78.0 ± 17.9	63 (41.7)	-3.6(-8.8 to 1.6)	0.115
6 months	81.1 ± 18.1	50 (33.1)	82.1 ± 18.7	74 (49.0)	1.0(-4.5 to 6.5)	0.602
**1 year**	**82.1 ± 18.0**	**82 (54.3)**	**78.0 ± 22.0**	**74 (49.0)**	**-4.1(-10.7 to 2.5)**	**0.371**
Physical Functioning						
Baseline	89.2 ± 20.8	80 (53.0)	89.2 ± 21.2	87 (57.6)	0.0(-7.2 to 7.2)	0.463
7 days	90.4 ± 17.8	68 (45.0)	90.8 ± 17.6	78 (51.7)	0.4(-5.2 to 6.0)	0.781
1 month	89.3 ± 21.8	50 (33.1)	90.9 ± 20.2	63 (41.7)	1.6(-4.4 to 7.6)	0.543
6 months	94.0 ± 14.9	50 (33.1)	91.6 ± 15.9	76 (50.3)	-2.4(-7.1 to 2.3)	0.216
**1 year**	**96.5 ± 9.8**	**82 (54.3)**	**94.4 ± 12.0**	**76 (50.3)**	**-2.1(-5.7 to 1.5)**	**0.777**
Role/Social Limitations Emotional						
Baseline	90.3 ± 21.6	80 (53.0)	93.2 ± 17.1	86 (57.0)	2.9(-3.7 to 9.5)	0.540
7 days	94.3 ± 15.4	65 (43.0)	91.6 ± 15.4	77 (51.0)	-2.7(-7.5 to 2.1)	0.158
1 month	95.4 ± 14.7	50 (33.1)	91.2 ± 22.1	63 (41.7)	-4.2(-9.7 to 1.3)	0.111
6 months	94.6 ± 15.2	50 (33.1)	96.2 ± 11.2	74 (49.0)	1.6(-2.3 to 5.5)	0.876
**1 year**	**95.8 ± 14.9**	**79 (52.3)**	**94.5 ± 16.1**	**72 (47.7)**	**-1.3(-6.3 to 3.7)**	**0.627**
Role/Social Limitations Behavioral						
Baseline	94.1 ± 15.0	80 (53.0)	93.0 ± 17.8	86 (57.0)	-1.1(-6.7 to 4.5)	0.460
7 days	96.7 ± 13.1	66 (43.7)	95.6 ± 13.5	77 (51.0)	-1.1(-5.3 to 3.1)	0.420
1 month	95.6 ± 15.2	50 (33.1)	93.1 ± 21.0	63 (41.7)	-2.5(-7.8 to 2.8)	0.660
6 months	97.5 ± 12.0	50 (33.1)	97.8 ± 9.7	74 (49.0)	0.3(-2.9 to 3.5)	0.639
**1 year**	**97.2 ± 11.5**	**79 (52.3)**	**96.2 ± 14.9**	**74 (49.0)**	**-1.0(-5.3 to 3.3)**	**0.855**
Role/Social Limitations Physical						

Baseline	61.6 ± 9.2	81 (53.6)	63.7 ± 10.1	87 (57.6)	2.1(-1.2 to 5.4)	0.079
7 days	62.7 ± 9.2	66 (43.7)	63.3 ± 6.9	79 (52.3)	0.6(-1.9 to 3.1)	0.944
1 month	64.0 ± 9.3	52 (34.4)	63.9 ± 7.7	63 (41.7)	-0.1(-2.6 to 2.4)	0.707
6 months	63.5 ± 8.4	51 (33.8)	64.4 ± 7.7	74 (49.0)	0.9(-1.5 to 3.3)	0.887
**1 year**	**64.5 ± 8.5**	**82 (54.3)**	**64.8 ± 8.1**	**74 (49.0)**	**0.3(-2.4 to 3.0)**	**0.576**
Bodily Pain/Discomfort						
Baseline	70.0 ± 22.4	82 (54.3)	70.0 ± 26.1	90 (59.6)	0.0(-8.5 to 8.5)	0.945
7 days	71.8 ± 23.2	69 (45.7)	72.6 ± 26.6	79 (52.3)	0.8(-7.2 to 8.8)	0.638
1 month	73.1 ± 23.1	53 (35.1)	73.5 ± 25.3	63 (41.7)	0.4(-6.6 to 7.4)	0.712
6 months	76.5 ± 24.5	52 (34.4)	74.8 ± 24.0	74 (49.0)	-1.7(-9.0 to 5.6)	0.514
**1 year**	**79.5 ± 23.0**	**85 (56.3)**	**76.9 ± 24.7**	**77 (51.0)**	**-2.6(-10.6 to 5.4)**	**0.583**
Behavior						
Baseline	84.8 ± 10.2	83 (55.0)	85.0 ± 10.3	89 (58.9)	0.2(-3.4 to 3.8)	0.868
7 days	84.1 ± 10.2	68 (45.0)	85.6 ± 10.7	79 (52.3)	1.5(-1.8 to 4.8)	0.920
1 month	84.4 ± 9.9	51 (33.8)	84.0 ± 9.8	64 (42.4)	-0.4(-3.3 to 2.5)	0.628
6 months	83.6 ± 9.5	53 (35.1)	83.2 ± 9.9	77 (51.0)	-0.4(-3.4 to 2.6)	0.723
**1 year**	**83.7 ± 9.2**	**83 (55.0)**	**84.7 ± 9.3**	**80 (53.0)**	**1.0(-2.1 to 4.1)**	**0.542**
Global Behavior Item						
Baseline	80.0 ± 16.8	88 (58.3)	79.2 ± 17.8	87 (57.6)	-0.8(-6.9 to 5.3)	0.893
7 days	77.3 ± 19.2	65 (43.0)	80.7 ± 15.7	78 (51.7)	3.4(-2.1 to 8.9)	0.361
1 month	78.1 ± 17.3	52 (34.4)	80.2 ± 15.5	67 (44.4)	2.1(-2.7 to 6.9)	0.454
6 months	79.4 ± 14.7	54 (35.8)	78.3 ± 18.4	79 (52.3)	-1.1(-6.3 to 4.1)	0.990
**1 year**	**82.0 ± 15.0**	**82 (54.3)**	**77.7 ± 18.0**	**78 (51.7)**	**-4.3(-9.8 to 1.2)**	**0.158**
Mental Health						
Baseline	79.3 ± 11.8	83 (55.0)	78.2 ± 11.8	86 (57.0)	-1.1(-5.2 to 3.0)	0.638
7 days	79.5 ± 11.4	68 (45.0)	76.6 ± 13.6	77 (51.0)	-2.9(-6.9 to 1.1)	0.297
1 month	78.6 ± 11.7	54 (35.8)	79.2 ± 11.1	66 (43.7)	0.6(-2.7 to 3.9)	0.802
6 months	78.2 ± 11.7	53 (35.1)	79.5 ± 10.9	76 (50.3)	1.3(-2.1 to 4.7)	0.562
**1 year**	**80.3 ± 10.8**	**82 (54.3)**	**78.2 ± 13.0**	**80(53.0)**	**-2.1(-6.1 to 1.9)**	**0.499**
Self Esteem						
Baseline	79.3 ± 10.4	83 (55.0)	81.3 ± 10.6	91 (60.3)	2.0(-1.7 to 5.7)	0.473
7 days	76.5 ± 13.7	73 (48.3)	77.4 ± 11.5	81 (53.6)	0.9(-3.2 to 5.0)	0.564
1 month	78.0 ± 12.4	69 (45.7)	79.3 ± 10.8	54 (35.8)	1.3(-2.2 to 4.8)	0.517
6 months	77.6 ± 11.6	56 (37.1)	78.9 ± 11.1	79 (52.3)	1.3(-2.2 to 4.8)	0.361

**1 year**	**79.6 ± 11.0**	**86 (57.0)**	**77.5 ± 12.6**	**84 (55.6)**	**-2.1(-6.2 to 2.0)**	**0.782**
General Health Perceptions						
Baseline	81.2 ± 12.8	83 (55.0)	83.1 ± 12.6	90 (59.6)	1.9(-2.5 to 6.3)	0.320
7 days	80.6 ± 15.2	70 (46.4)	82.3 ± 14.9	80 (53.0)	1.7(-3.1 to 6.5)	0.453
1 month	81.8 ± 14.4	52 (34.4)	79.6 ± 17.2	68 (45.0)	-2.2(-6.9 to 2.5)	0.641
6 months	80.6 ± 14.3	53 (35.1)	82.7 ± 15.3	76 (50.3)	2.1(-2.4 to 6.6)	0.151
**1 year**	**83.2 ± 11.9**	**84 (55.6)**	**82.7 ± 13.5**	**78 (51.7)**	**-0.5(-4.8 to 3.8)**	**0.943**
Family Activities						
Baseline	90.4 ± 13.7	83 (55.0)	90.7 ± 10.6	89 (58.9)	0.3(-3.9 to 4.5)	0.534
7 days	90.1 ± 12.8	69 (45.7)	88.5 ± 13.3	78 (51.7)	-1.6(-5.8 to 2.6)	0.474
1 month	90.1 ± 12.6	54 (35.8)	90.2 ± 13.0	68 (45.0)	0.1(-3.7 to 3.9)	0.945
6 months	88.4 ± 12.8	56 (37.1)	89.6 ± 13.7	78 (51.7)	1.2(-2.9 to 5.3)	0.481
**1 year**	**90.5 ± 11.3**	**85 (56.3)**	**91.6 ± 12.1**	**80 (53.0)**	**1.1(-2.9 to 5.1)**	**0.460**
Family Cohesion						
Baseline	80.4 ± 17.5	83 (55.0)	80.0 ± 19.9	91 (60.3)	-0.4(-7.0 to 6.2)	0.843
7 days	75.9 ± 21.3	69 (45.7)	78.5 ± 20.7	78 (51.7)	2.6(-4.1 to 9.3)	0.397
1 month	78.1 ± 20.0	54 (35.8)	80.2 ± 16.0	68 (45.0)	2.1(-3.2 to 7.4)	0.760
6 months	76.3 ± 18.5	57 (37.7)	77.2 ± 20.9	79 (52.3)	0.9(-5.3 to 7.1)	0.542
**1 year**	**80.2 ± 19.0**	**86 (57.0)**	**76.7 ± 19.4**	**81 (53.6)**	**-3.5(-10.0 to 3.0)**	**0.259**

Table S3. PSQ-18

**Patient Satisfaction Questionnaire-18 Domains** (mean ± SD) Scores are reported on a scale from 0-100	**Appendectomy** *(n=151)*	**Missing data** (n/% of total)	**NOT strategy** *(n=151)*	**Missing data** (n/% of total)	**Mean difference** (95%CI)	**p-value**
General satisfaction						
7 days	79.0 ± 14.3	67 (44.4%)	78.0 ± 16.0	52 (34.4%)	-1.0 (-5.4 to 3.4)	0.583
1 month	79.0 ± 14.0	73 (48.3%)	76.8 ± 16.0	67 (44.4%)	-2.2 (-6.9 to 2.5)	0.393
**1 year**	**78.0 ± 13.0**	**76 (50.3%)**	**77.2 ± 16.1**	**79 (52.3%)**	**-0.8 (-5.6 to 4.0)**	**0.989**
Technical Quality						
7 days	80.1 ± 10.0	67 (44.4%)	79.0 ± 13.0	52 (34.4%)	-1.1 (-4.5 to 2.3)	0.644
1 month	80.1 ± 11.5	75 (49.7%)	78.2 ± 12.3	67 (44.4%)	-1.9 (-5.6 to 1.8)	0.361
**1 year**	**76.8 ± 10.8**	**78 (51.7%)**	**76.4 ± 13.2**	**78 (51.7%)**	**-0.4 (-4.4 to 3.6)**	**0.859**
Interpersonal manner						
7 days	83.9 ± 11.2	67 (44.4%)	83.1 ± 11.7	52 (34.4%)	-0.8 (-4.2 to 2.6)	0.681
1 month	83.0 ± 10.5	75 (49.7%)	81.0 ± 12.0	68 (45.0%)	-2.0 (-5.5 to 1.5)	0.265
**1 year**	**81.5 ± 9.4**	**78 (51.7%)**	**79.4 ±13.1**	**83 (55.0%)**	**-2.1 (-5.9 to 1.7)**	**0.436**
Communication						
7 days	82.3 ± 12.1	67 (44.4%)	81.8 ± 11.2	52 (34.4%)	-0.5 (-3.9 to 2.9)	0.720
1 month	82.5 ± 11.3	71 (47.0%)	79.1 ± 12.6	66 (43.7%)	-3.4 (-7.1 to 0.3)	0.052
**1 year**	**79.2 ± 11.8**	**76 (50.3%)**	**81.2 ± 12.7**	**78 (51.7%)**	**2.0 (-2.0 to 6.0)**	**0.352**
Financial aspects						
7 days	81.4 ± 14.7	67 (44.4%)	82.2 ± 13.2	52 (34.4%)	0.8 (-3.3 to 4.9)	0.778
1 month	84.4 ± 11.5	73 (48.3%)	82.0 ± 13.9	67 (44.4%)	-2.4 (-6.4 to 1.6)	0.358
**1 year**	**78.0 ± 14.4**	**77 (51.0%)**	**79.6 ± 13.1**	**80 (53.0%)**	**1.6 (-2.9 to 6.1)**	**0.615**
Time spent with doctor						
7 days	75.4 ± 11.2	67 (44.4%)	74.9 ± 15.4	52 (34.4%)	-0.5 (-4.4 to 3.4)	0.900
1 month	75.4 ± 12.8	75 (49.7%)	72.7 ± 15.6	68 (45.0%)	-2.7 (-7.2 to 1.8)	0.260
**1 year**	**72.1 ± 13.9**	**78 (51.7%)**	**71.6 ± 14.4**	**83 (55.0%)**	**-0.5 (-5.2 to 4.2)**	**0.667**
Accessibility and convenience						
7 days	72.6 ± 11.0	67 (44.4%)	72.7 ± 13.1	52 (34.4%)	0.1 (-3.4 to 3.6)	0.977
1 month	74.8 ± 10.9	77 (51.0%)	73.3 ± 14.4	68 (45.0%)	-1.5 (-5.5 to 2.5)	0.925
**1 year**	**71.8 ± 10.9**	**78 (51.7%)**	**71.4 ± 13.9**	**81 (53.6%)**	**-0.4 (-4.5 to 3.7)**	**0.852**
