# Supplementary material for: Non-operative treatment strategy versus surgery for children with simple appendicitis: non-inferiority randomised controlled trial
Source: BMJ Med. 2026 May 13;5(1):e002466. doi: 10.1136/bmjmed-2025-002466 (PMC13182491; doi:10.1136/bmjmed-2025-002466)
Supplement: Supplementary file 4 [file bmjmed-2025-002466.reviewer_comments.pdf]

PEER REVIEW HISTORY

BMJ Medicine publishes all reviews undertaken for accepted manuscripts. Reviewers are asked to complete a checklist review form and are provided with free text boxes to elaborate on their assessment. These free text comments are reproduced below.

This paper was submitted to another journal from BMJ but declined for publication following peer review. The authors addressed the reviewers' comments and submitted the revised paper to BMJ Medicine. The paper was subsequently accepted for publication at BMJ Medicine.

ARTICLE DETAILS

**TITLE (PROVISIONAL)**	Non-operative treatment strategy versus surgery for children with simple appendicitis - a non-inferiority randomized controlled trial (APAC trial)
**AUTHORS**	Bachiri, Said; van Amstel, Paul; Knaapen, Max; van der Lee, Johanna; van Eekelen, Rik; Bemelman, Willem; Rippen-Wagner, Hester; Bijlsma, Taco; Blanken-Peeters, Charlotte; Boerma, Evert-Jan; van den Boom, Anne Loes; van den Broek, Frank; Cense, Huib; van Duijvendijk, Peter; Garssen, Frank; in 't Hof, Klaas H; Leijdekkers, Vanessa; Lijkwan, Maarten; Zijp, Gerda; Heij, Hugo; Derikx, Joep; van Heurn, Ernst; Bakx, Roel; Gorter, Ramon; APAC collaborative, study group

VERSION 1 - REVIEW

**REVIEWER NAME**	Mentula, Panu
**REVIEWER AFFILIATION**	Helsinki University Hospital
**REVIEWER CONFLICT OF INTEREST**	Lecture fee from Pfizer<br>Institutional reasearch grant from Finnish government
**DATE REVIEW RETURNED**	24-Oct-2025

**GENERAL COMMENTS**	In overall, this study is an excellent study without major flaws. The main findings are not surprising and are in line with the earlier studies on same subject. One could argue that excluding failure of non-operative management from complications of non-operative treatment is not solid. However, even excluding these, nonoperative management was not non-inferior to surgical treatment.<br>In the Introduction there is a sentence: " Another disadvantage of intra-abdominal adhesions is the potential impairment of female fertility". I strongly disagree with this statement. There is no scientific evidence that appendectomy impairs female fertility. (refs: Andersson R et al. Fertility Patterns after Appendicectomy: Historical Cohort Study. BMJ 318, 1999: 963–67 and Männistö J et al. Association of Complicated Appendicitis on the Risk of Later in Vitro Fertilization Treatment Requirement and Ectopic Pregnancy: A Nationwide Cohort Study." Acta Obstetricia et Gynecologica Scandinavica 100, 2021: 1490–96.) If shared decision making is suggested in selection of treatment of appendicitis, it is important that infertility is not in the list of the disadvantages of surgical treatment.<br>In the discussion, there is limited discussion on harms of antibiotics. Globally, increased antibiotic resistance would encourage doctors to avoid antibiotic use if not necessary. Also, childhood antibiotic usage is associated with increased incidence of inflammatory bowel

	disease. There is strong evidence from both epidemiological studies and from recent RCTs on adult patients that mild appendicitis can resolve spontaneously. Taking these findings into account the suggested step-up strategy with non-operative management might be better without antibiotics.<br>In the conclusions patient satisfaction requires more attention. Results of Net Promoter Score should have more visibility in the abstract and conclusions. Also, higher number of extra visits needs to be acknowledged in the abstract and conclusions. Now it seems that authors are trying to make non-operative treatment look more attractive than it is. Disadvantages of non-operative treatment are not reported in the abstract at all.

**REVIEWER NAME**	Hall, Nigel
**REVIEWER AFFILIATION**	University of Southampton
**REVIEWER CONFLICT OF INTEREST**	None
**DATE REVIEW RETURNED**	24-Oct-2025

**GENERAL COMMENTS**	Congratulations on completing this challenging but important RCT. This clearly makes a significant contribution to the field and confirms that non-operative management is a reasonable alternative to surgery. Whilst the strict non-inferiority criteria were not met the real value of this work in my opinion is the depth of data on the full range of secondary outcomes that will inform shared decision making (as the authors propose) in the future. I have some small suggestions for improvement / clarification and would suggest a complete rewrite of the discussion in the interest of being much more succinct.<br><br>INTRO<br>Line 5 I'm afraid I disagree with your first statement. The feasibility of this approach is well recognised and proven a long time ago. You may mean there is interest in the comparative outcomes compared to appendicectomy? Please revise<br><br>Line 25 - are we so certain about adhesions being related to surgery rather than the disease process of appendicitis?<br><br>Overall in this first paragraph I appreciate you are aiming to present a balance of risks and benefits with each treatment approach. I am not sure you have got it quite correct or even selected the most important risks and benefits.<br><br>P8 lines 3-20 You may wish to recognise the Australian trial as well?<br><br>METHODS<br>P9 Line 52 - was the only stratification variable 'hospital'? No sex, age etc?<br><br>P 10 Line 9 why did you do a repeat US prior to discharge home?<br><br>P10 Line 19 'predefined signs of clinical deterioration' suggest referencing Figure 1 here rather than just lower down.<br><br>P11 - line 48 how is interval appendectomy possible in this trial?<br><br>P12 - for all outcomes please state at what timepoint they were assessed. E.g. QoL - how many times and when?

	P13 Lines 7-14 - I don't understand your description here. You state that you expected 'NOT to be favourable in terms of complications....' but then later willing to accept an up to 5% higher chance of complications. The usual premise for a non-inferiority trial is that you are willing to accept something that is expected to be worse as being worse. You then go on to state that in fact you assumed the NOT complication rate would be less than that with surgery. I am confused by this.<br><br>P13 lines 45-46 - please explain what the difference between the ITT, PP and AT analyses were - which patients moved groups in which analyses?<br><br><br>RESULTS<br><br>Fig2 - your 'CONSORT' flowchart - it would be helpful to continue this to show how many in each group withdrew and how many in each group had primary outcome data available. Do you have ANY data on reasons for non-enrolment?<br><br><br>DISCUSSION<br><br>Whilst this is thorough it is also extremely long and could easily be shortened without losing meaning. It is also somewhat repetitive in places. It really would benefit from significant shortening.

**REVIEWER NAME**	Fonnes, Siv
**REVIEWER AFFILIATION**	Gastrounit, Department of Surgery, Amager Hvidovre Hospital
**REVIEWER CONFLICT OF INTEREST**	None
**DATE REVIEW RETURNED**	24-Oct-2025

**GENERAL COMMENTS**	Congratulations on conducting a fine RCT in a difficult acute setting and population to contribute to and increase our knowledge and evidence for treating uncomplicated appendicitis in children. Your hard efforts are an important contribution to the field and will aid future shared decision-making in this population. To increase the transparency of your reporting and to mitigate future risk of bias assessments of your RCT, I have made comments focused on increasing the article's transparency. Furthermore, I have made comments on statistical vs. clinical significance to improve the focus on the important message and the readability of the article to future readers. I hope this can make your article even more readable and will help it gain the impact it deserves. Please see my specific comments below.<br><br>Comments<br>1. Abstract: Please add ' parallel' to the method section (CONSORT abstract # trial design).<br>2. Abstract: Consider revising 'imaging confirmed' to 'at least ultrasound confirmation of' as this seems more in line with your protocol/method section.<br>3. Abstract: Please also describe the intervention for the appendectomy group e.g. specify if it was a laparoscopic appendectomy, open appendectomy or both. Please also add if they

	received antibiotic cover during surgery (CONSORT abstract # Interventions).<br>4. Abstract: Please add that the trial was naturally unblinded for transparent reporting (CONSORT abstract # Blinding).<br>5. Abstract: In your intention-to-treat analysis, you have missing data for two participants on complications at 1 year, however, this is not reflected in the number in the abstract. Please correct (CONSORT abstract # Numbers analysed).<br>6. Trial registration: Please remember to update the status of your trial (NCT02848820).<br>7. Please make the data sharing statement even clearer (CONSORT #4)<br>8. Please state the role of funders more clearly (COONSORT #5).<br>9. Introduction: The introduction section is a bit too long and spans almost 2 pages. Especially the middle section on previous studies can be shortened and less detailed. Some of the details are also discussed in your discussion section.<br>10. Introduction: The rationale for a non-inferiority design is missing; please add (CONSORT non-inferiority trials extension #2a+b).<br>11. Method, study design: Please provide a bit more information on the setting e.g. type of hospital: "The study was conducted in 15 hospitals in the Netherlands" (CONSORT #11).<br>12. Methods, Study setting and procedures: Please provide a bit more information on the control group that received appendectomy: laparoscopic appendectomy, open appendectomy or both, as these carry different risk of complications in adults, as seen in the Cochrane review, that you also cite. Did they receive antibiotic cover during surgery? In clinicaltrials.gov and your protocol, it only states "according to local protocol". Please provide more information (CONSORT #13).<br>13. Methods, randomization: Please describe on the block size e.g. fixed or different as this is important to evaluate the risk of bias of the RCT.<br>14. Methods, randomization: Was the data analysis blinded, please specify as this may impact the risk of bias assessment of the trial (CONSORT #20).<br>15. Figure 2: This CONSORT flow diagram contains too little information, please add A) reasons for exclusions besides parental consent e.g. fulfilled any of the exclusion criteria if applicable, B) allocation to intervention should clearly report if the allocated intervention was received or not (and if not, reasons should be given), C) lost to follow-up should be placed below allocation and should be reported for both groups to increase transparency, and D) addition of available for final analysis (CONSORT #22+24)<br>16. Results: Please provide the baseline data on laparoscopic and open appendectomy, e.g. just in the text. Table 1: Please provide a footnote giving more detail on "Allergies to medication" and "Past relevant illnesses". What past relevant illnesses are considered relevant to appendicitis?<br>17. Results: Please move the reference to "Types of complications are reported in Table 7." from the section on secondary outcomes to the section on the primary outcome, e.g. complications.<br>18. Table 2+3: I recommend that you transparently report your limited missing data in another way than to reserve two whole columns for this number in all these tables. The present presentation emphasises too much focus on this rather than the actual results, thus, increasing the non-data ink. If you instead report it in a footnote, it is still transparent and available but the important messages of your tables, the data, become even clearer while transparency is maintained.

	19. Table 1a: As noted in the comments for the abstract, you must have missing data on the 1-year outcome of complications for two patients, the one who could not be reached and the one who withdrew consent. This is not clear from the numbers in the table.<br>20. Table 1a: The 95% CI for proportions in each group is not reported; please add these (CONSORT #26 and the statistical reporting guideline SAMPL by Lang et Altman).<br>21. Results, secondary outcomes: Please consider if the statistically significant difference of a bit more than half a day on pain medication is a clinically relevant one.<br>22. Results, secondary outcomes: Similarly, is the statistical difference in HR-QoL clinically relevant – how much does the scale need to change for the patients to feel the difference in this tool?<br>23. Discussion: Your discussion section spans more than six pages and around 3,000 words. Please consider revising and shortening it. My recommendations include: A) the first paragraph of the discussion should be short and written in prose, summarising the most important findings (avoid specific percentages to increase readability) of the RCT. B) The section directly comparing your RCT to reference 16 spans almost 2 pages, and it is too detailed; consider keeping only the most important points. C) The section on harms is interesting and should be retained; however, please consider making it shorter. D) Consider if discussion HR-QoL is highly relevant here, with my comment on statistical vs. clinically relevant differences, in mind from the results section – this section could maybe be omitted.<br>24. Limitation: Please also ensure to mention the limitation on open vs. laparoscopic appendectomy and complication rates.

VERSION 1 – AUTHOR RESPONSE

Independent reviewer 1

Comment 1:
"In overall, this study is an excellent study without major flaws. The main findings are not surprising and are in line with the earlier studies on same subject. One could argue that excluding failure of non-operative management from complications of non-operative treatment is not solid. However, even excluding these, nonoperative management was not non-inferior to surgical treatment.

In the Introduction there is a sentence: " Another disadvantage of intra-abdominal adhesions is the potential impairment of female fertility". I strongly disagree with this statement. There is no scientific evidence that appendectomy impairs female fertility. (refs: Andersson R et al. Fertility Patterns after Appendicectomy: Historical Cohort Study. BMJ 318, 1999: 963–67 and Männistö J et al. Association of Complicated Appendicitis on the Risk of Later in Vitro Fertilization Treatment Requirement and Ectopic Pregnancy: A Nationwide Cohort Study." Acta Obstetricia et Gynecologica Scandinavica 100, 2021: 1490–96.) If shared decision making is suggested in selection of treatment of appendicitis, it is important that infertility is not in the list of the disadvantages of surgical treatment.

In the discussion, there is limited discussion on harms of antibiotics. Globally, increased antibiotic resistance would encourage doctors to avoid antibiotic use if not necessary. Also, childhood antibiotic usage is associated with increased incidence of inflammatory bowel disease. There is strong evidence from both epidemiological studies and from recent RCTs on adult patients that mild appendicitis can resolve spontaneously. Taking these findings into account the suggested step-up strategy with non-operative management might be better without antibiotics.
In the conclusions patient satisfaction requires more attention. Results of Net Promoter Score should have more visibility in the abstract and conclusions. Also, higher number of extra visits needs to be acknowledged in the abstract and conclusions. Now it seems that authors are trying to make non-operative treatment look more attractive than it is. Disadvantages of non-operative treatment are not reported in the abstract at all.

Reply 1:
First of all, we would like to thank the reviewer for their efforts to critically appraise our manuscript and give advice for further improvement.

We are also thankful for the insight and references regarding the impact of surgery/abdominal inflammation (in our case due to appendicitis) on female fertility. We have added these references to our manuscript and aimed to introduce more nuance to this matter/discussion.

We have also added more focus in our discussion to the impact of childhood antibiotic administration and development of IBD later in life. Also, we have addressed the matter of global antimicrobial resistance more thoroughly.

Finally, we have adjusted the text highlighting the findings about patient satisfaction, higher number of extra visits and other disadvantages of a NOT strategy.

Changes in the text:
See page 6. Lines 153-157.
See page 19. Lines 613-617
See page 19. Lines 619-652.

Independent reviewer 2

Comment 1: *"Congratulations on completing this challenging but important RCT. This clearly makes a significant contribution to the field and confirms that non-operative management is a reasonable alternative to surgery. Whilst the strict non-inferiority criteria were not met the real value of this work in my opinion is the depth of data on the full range of secondary outcomes that will inform shared decision making (as the authors propose) in the future. I have some small suggestions for improvement / clarification and would suggest a complete rewrite of the discussion in the interest of being much more succinct."*

Reply 1: We want to thank you for your efforts and critical appraisal of our manuscript and we are grateful for your acknowledgement of this important RCT and suggestions for improvement. The suggestion to shorten the discussion section shall be addressed later on in this letter.

Changes in the text: None.

Comment 2: *"INTRO Line 5 I'm afraid I disagree with your first statement. The feasibility of this approach is well recognised and proven a long time ago. You may mean there is interest in the comparative outcomes compared to appendicectomy? Please revise"*

Reply 2: Thank you for pointing out this subtle but important discrepance. We have revised the text accordingly.

Changes in the text: See page 6. Lines 138-140.

Comment 3: *"Line 25 - are we so certain about adhesions being related to surgery rather than the disease process of appendicitis?"*

Reply 3: Thank you for pointing out this matter. Reviewer 1 had similar concerns. Due to the lack of high quality evidence supporting this, we have decided to remove this text from the introduction.

Changes in the text: See Introduction. Page 6.

Comment 4: *"Overall in this first paragraph I appreciate you are aiming to present a balance of risks and benefits with each treatment approach. I am not sure you have got it quite correct or even selected the most important risks and benefits."*

Reply 4: We aimed to highlight a brief overview of associated pros and cons of both treatment strategies in the introduction. In our discussion section we have aimed to discuss these more in depth alongside the findings of our RCT.

Changes in the text: None.

Comment 5: *"P8 lines 3-20 You may wish to recognise the Australian trial as well?"*

Reply 5: Thank you for highlighting the Australian trial. We have decided to reference this trial in our introduction.

Changes in the text: See page 6. Lines 169-172.

Comment 6: "METHODS P9 Line 52 - was the only stratification variable 'hospital'? No sex, age etc?"

Reply 6: This is correct, 1:1 block randomization was stratified only by hospital, not for other variables.

Changes in the text: None.

Comment 7: *"P 10 Line 9 why did you do a repeat US prior to discharge home?"*

Reply 7: The repeat ultrasound was an extra safety measure in our protocol. Potentially missed faecoliths during the primary ultrasound could be seen or any other signs of deterioration to complex appendicitis could be identified which could play a role in the decision to opt for surgery.

Changes in the text: None.

Comment 8: *"P10 Line 19 'predefined signs of clinical deterioration' suggest referencing Figure 1 here rather than just lower down."*

Reply 8: We have changed the position of the reference to Figure 1 accordingly.

Changes in the text: See page 9. Line 249.

Comment 9: *"P11 - line 48 how is interval appendectomy possible in this trial?"*

Reply 9: There were 2 children where an interval appendectomy was performed within 1 year of a NOT strategy. These 2 did not have a recurrence, but after shared decision making between parents and surgeon there was decided to remove the appendix surgically in a planned setting due to consistent abdominal complaints/symptoms. We have added a short clarification in the text.

Changes in the text:
See page 10. Lines 288-291.
See page 10. Lines 304.

Comment 10: *"for all outcomes please state at what timepoint they were assessed. E.g. QoL - how many times and when?"*

Reply 10: We have adjusted accordingly.

Changes in the text: See page 10. Lines 296-343.

Comment 11: *"P13 Lines 7-14 - I don't understand your description here. You state that you expected 'NOT to be favourable in terms of complications....' but then later willing to accept an up to 5% higher chance of complications. The usual premise for a non-inferiority trial is that you are willing to accept something that is expected to be worse as being worse. You then go on to state that in fact you assumed the NOT complication rate would be less than that with surgery. I am confused by this."*

Reply 11: We understand that this might initially sound a bit confusing. Based on the available literature at the time this RCT was designed we indeed expected that a NOT strategy would lead to

less complications compared to appendectomy. However, for implementation in clinical practice and to convince the medical field, we wanted to at least demonstrate non-inferiority of a NOT strategy. We wanted to at least demonstrate that it was less than 5% worse compared to appendectomy in terms of proportion of patients with a complication at 1-year follow-up. We have aimed to clarify this even more.

Changes in the text: See page 11. Lines 347-351.

Comment 12: *"P13 lines 45-46 - please explain what the difference between the ITT, PP and AT analyses were - which patients moved groups in which analyses?"*

Reply 12: We have added clarification in the text about the different analyses.

Changes in the text: See page 12. Lines 375-381.

Comment 13: *"RESULTS Fig2 - your 'CONSORT' flowchart - it would be helpful to continue this to show how many in each group withdrew and how many in each group had primary outcome data available. Do you have ANY data on reasons for non-enrolment?"*

Reply 13: We have added information about withdrawal to the CONSORT flowchart. Unfortunately, we do not possess any data about reasons for non-enrolment.

Changes in the text: See page 26.

Comment 14: "DISCUSSION Whilst this is thorough it is also extremely long and could easily be shortened without losing meaning. It is also somewhat repetitive in places. It really would benefit from significant shortening."

Reply 14: We have aimed to shorten the discussion without losing any importance or relevance of the message.

Changes in the text: See Discussion. Pages 15-21

Independent reviewer 3:

Comment 1: *"Abstract: Please add ' parallel' to the method section (CONSORT abstract # trial design)."*

Reply 1: We have added the suggested addition to the abstract and methods section.

Changes in the text:
See page 5. Line 113.
See page 7. Line 187.

Comment 2: *"Abstract: Consider revising 'imaging confirmed' to 'at least ultrasound confirmation of' as this seems more in line with your protocol/method section."*

Reply 2: Thank you for your suggestive comment. We have decided to keep the use of 'imaging confirmed' as there were participants where ultrasound was inconclusive and the diagnosis was confirmed by MRI scan.

Changes in the text: None.

Comment 3: *"Abstract: Please also describe the intervention for the appendectomy group e.g. specify if it was a laparoscopic appendectomy, open appendectomy or both. Please also add if they received antibiotic cover during surgery (CONSORT abstract # Interventions)."*

Reply 3: We have added more detailed description of the surgical intervention in the methods section and also added reporting of this in the Results section. While surgical approach was at the discretion of the surgeon. All surgically managed patients were approached laparoscopically.

Changes in the text:
See page 9. Lines 253-254.
See page 13. Line 407.

Comment 4: *"Abstract: Please add that the trial was naturally unblinded for transparent reporting (CONSORT abstract # Blinding)."*

Reply 4: We have added the suggested addition to the abstract and methods section

Changes in the text:
See page 5. Lines 113.
See page 7. Lines 187.

Comment 5: *"Abstract: In your intention-to-treat analysis, you have missing data for two participants on complications at 1 year, however, this is not reflected in the number in the abstract. Please correct (CONSORT abstract # Numbers analysed)."*

Reply 5: Although there were indeed two participants that did not reach the end-point, these participants did experience a complication while in active follow-up and could therefore be included in the primary outcome ITT analysis. That clarifies why the missing data is not reported here. We have added more clarifying text in Figure 2.

Changes in the text: See page 26. Lines 877-879.

Comment 6: *"Trial registration: Please remember to update the status of your trial (NCT02848820)."*

Reply 6: We have updated the trial status.

Changes in the text: None.

Comment 7: *"Please make the data sharing statement even clearer (CONSORT #4)"*

Reply 7: We have clarified the data sharing statement

Changes in the text: See page 3. Line 70.

Comment 8: *"Please state the role of funders more clearly (COONSORT #5)."*

Reply 8: We have stated the role of the funders more clearly.

Changes in the text: See page 3. Lines 62-64.

Comment 9: *"Introduction: The introduction section is a bit too long and spans almost 2 pages. Especially the middle section on previous studies can be shortened and less detailed. Some of the details are also discussed in your discussion section."*

Reply 9: We have aimed to shorten the introduction.

Changes in the text: See Introduction. Pages 6-7.

Comment 10: *"Introduction: The rationale for a non-inferiority design is missing; please add (CONSORT non-inferiority trials extension #2a+b)."*

Reply 10: We have added the rationale for a non-inferiority design in the introduction.

Changes in the text: See page 7. Lines 175-183.

Comment 11: *"Method, study design: Please provide a bit more information on the setting e.g. type of hospital: "The study was conducted in 15 hospitals in the Netherlands" (CONSORT #11)."*

Reply 11: We have added more information about the study setting.

Changes in the text: See page 7. Lines 188-189.

Comment 12: *"Methods, Study setting and procedures: Please provide a bit more information on the control group that received appendectomy: laparoscopic appendectomy, open appendectomy or both, as these carry different risk of complications in adults, as seen in the Cochrane review, that you also cite. Did they receive antibiotic cover during surgery? In clinicaltrials.gov and your protocol, it only states "according to local protocol". Please provide more information (CONSORT #13)."*

Reply 12: We have aimed to clarify this in the method section text.

Changes in the text: See page 9. Lines 253-254.

Comment 13: *"Methods, randomization: Please describe on the block size e.g. fixed or different as this is important to evaluate the risk of bias of the RCT."*

Reply 13: We have clarified this in the method section.

Changes in the text: See page 8. Lines 232-234.

Comment 14: *"Methods, randomization: Was the data analysis blinded, please specify as this may impact the risk of bias assessment of the trial (CONSORT #20)."*

Reply 14: We have added a statement regarding the blinding aspect of the data analysis.

Changes in the text: See page 8. Lines 234-236.

Comment 15: *"Figure 2: This CONSORT flow diagram contains too little information, please add A) reasons for exclusions besides parental consent e.g. fulfilled any of the exclusion criteria if applicable, B) allocation to intervention should clearly report if the allocated intervention was received or not (and if not, reasons should be given), C) lost to follow-up should be placed below allocation and should be reported for both groups to increase transparency, and D) addition of available for final analysis (CONSORT #22+24)"*

Reply 15:We have updated the flowchart with the required information.

Changes in the text: See page 26.

Comment 16: *"Results: Please provide the baseline data on laparoscopic and open appendectomy, e.g. just in the text. Table 1: Please provide a footnote giving more detail on "Allergies to medication" and "Past relevant illnesses". What past relevant illnesses are considered relevant to appendicitis?"*

Reply 16: All patients managed surgically were approached laparoscopically. No open appendectomies were performed.

Changes in the text: None.

Comment 17: *"Results: Please move the reference to "Types of complications are reported in Table 7." from the section on secondary outcomes to the section on the primary outcome, e.g. complications."*

Reply 17: We have transferred the reference and written out the complication profiles of both treatment strategies in this section.

Changes in the text: See page 14. Lines 429-433.

Comment 18: *"Table 2+3: I recommend that you transparently report your limited missing data in another way than to reserve two whole columns for this number in all these tables. The present*

presentation emphasises too much focus on this rather than the actual results, thus, increasing the non-data ink. If you instead report it in a footnote, it is still transparent and available but the important messages of your tables, the data, become even clearer while transparency is maintained."

Reply 18: We understand that readability may be compromised by the two columns representing the missing data. However, in the interest of transparency, we have decided to consistently report this for all outcomes.

Changes in the text: None

Comment 19: *"Table 1a: As noted in the comments for the abstract, you must have missing data on the 1-year outcome of complications for two patients, the one who could not be reached and the one who withdrew consent. This is not clear from the numbers in the table."*

Reply 19: Please see Reply 5.

Changes in the text: None.

Comment 20: *"Table 1a: The 95% CI for proportions in each group is not reported; please add these (CONSORT #26 and the statistical reporting guideline SAMPL by Lang et Altman)."*

Reply 20: We have chosen not to present this for our baseline Table. We did however statistically analyze the variables and found no statistically difference in any baseline characteristic between the treatment groups as would be expected in a RCT.

Changes in the text: None.

Comment 21: *"Results, secondary outcomes: Please consider if the statistically significant difference of a bit more than half a day on pain medication is a clinically relevant one."*

Reply 21: We agree with your remark about clinical relevance. However, in the result section we aim to present the raw outcomes of our data without coupling interpretation to this. Interpretation should be reserved for the discussion section.

Changes in the text: None.

Comment 22: *"Results, secondary outcomes: Similarly, is the statistical difference in HR-QoL clinically relevant – how much does the scale need to change for the patients to feel the difference in this tool?"*

Reply 22: Please see Reply 21.

Changes in the text: None.

Comment 23: *"Discussion: Your discussion section spans more than six pages and around 3,000 words. Please consider revising and shortening it. My recommendations include: A) the first paragraph of the discussion should be short and written in prose, summarising the most important findings (avoid specific percentages to increase readability) of the RCT. B) The section directly comparing your RCT to reference 16 spans almost 2 pages, and it is too detailed; consider keeping only the most important points. C) The section on harms is interesting and should be retained; however, please consider making it shorter. D) Consider if discussion HR-QoL is highly relevant here, with my comment on statistical vs. clinically relevant differences, in mind from the results section – this section could maybe be omitted."*

Reply 23: We have aimed to shorten the discussion without losing any importance or relevance of the message.

Changes in the text: See Discussion. Pages 15-21.

Comment 24: *"Limitation: Please also ensure to mention the limitation on open vs. laparoscopic appendectomy and complication rates."*

Reply 24: Please see reply 16. Only laparoscopic appendectomies were performed.

Changes in the text: None.

References:

1. Knaapen M, Van der Lee JH, Gaillard EL, et al. Non-operative treatment of children with simple appendicitis: long-term follow-up (5 years) in a prospective cohort study. Br J Surg 2021;108(3):e99–e100. DOI: 10.1093/bjs/znaa052.

VERSION 2 – REVIEW

**REVIEWER NAME**	Hall, Nigel
**REVIEWER AFFILIATION**	
**REVIEWER CONFLICT OF INTEREST**	None
**DATE REVIEW RETURNED**	28-Dec-2025

**GENERAL COMMENTS**	I have previously reviewed this manuscript and find that the majority of my comments have been addressed satisfactorily.<br>I still think the discussion could be shortened significantly more without losing meaning but leave this to an editorial decision.<br><br>You may wish to comment on the fragility of your main outcome result. What would have happened if just one more patient in the appendicectomy arm had a complication making the risk difference zero?<br><br>There are a few typos<br>- capital H line 45 on page 21, same x 2 in line 57 page 22, line 9 page 31, line 35 page 32<br>- page 30 line 38 'an' should be 'and'

**REVIEWER NAME**	Mentula, Panu
**REVIEWER AFFILIATION**	
**REVIEWER CONFLICT OF INTEREST**	None
**DATE REVIEW RETURNED**	29-Dec-2025

**GENERAL COMMENTS**	This is my second time reviewing this manuscript. Most of the issues have been addressed adequately. Since author's suggest shared decision making, I'd like to see also negative findings of NOT strategy in the abstract to give more balanced view of the findings. These would include longer hospital stay, more extra visits and lower NPS score with NOT strategy. Healthcare costs are less relevant in shared decision making.

**REVIEWER NAME**	Fonnes, Siv
**REVIEWER AFFILIATION**	
**REVIEWER CONFLICT OF INTEREST**	None

**DATE REVIEW RETURNED**	29-Dec-2025

**GENERAL COMMENTS**	The authors have revised the manuscript accordingly. I do still find that the current presentation of the tables with multiple columns reserved for missing data decreases the readability of the tables because less emphasis is on the actual data, and that it could be just as transparently reported using footnotes. This issue could be addressed in copy editing if needed.<br>I have no further comments and congratulate the authors on their conduct and reporting of the RCT.

**REVIEWER NAME**	Riley, Richard
**REVIEWER AFFILIATION**	University of Birmingham, Institute of Applied Health Research
**REVIEWER CONFLICT OF INTEREST**	None
**DATE REVIEW RETURNED**	05-Jan-2026

**GENERAL COMMENTS**	I thank the authors for addressing my statistical comments from the Editorial meeting when they submitted to the BMJ. Generally these responses, and the changes, are appropriate. However, there remain a number of crucial suggestions and clarifications for the next revision, which I will look at, as follows:<br><br>1) I thank the authors for calculating the probability that the NOT group have an increase of complications of 5% or less. This is given as 90.7% in their response. However, in the paper, the authors should focus more on this, e.g. in the abstract, which currently says 'Non-inferiority of a NOT strategy for acute simple appendicitis in children could<br>not be statistically demonstrated, but the majority of the 95%CI fell within the 5% noninferiority margin.' – it would be far stronger and clearer to also add the probability of falling <=5% here too.<br><br>2) With this in mind, the authors have added that 'We used confidence distributions (based on the confidence interval of the difference in proportions between groups) to state the estimated probability that the NOT strategy led to an increase in complications of 5% or less" … what is unclear is what a confidence distribution is. No reference is given. I think (assume) that they take the sampling distribution (of the risk difference) as approximately normal and work out the proportion <=5%? If not, then how?<br><br>3) 'A list of potential complications has been reported in the previously published study protocol' – this is so fundamental, that the list must be reported here too. Perhaps the list is already given when (some of) the outcomes are provided later in the methods text – if so, then state this to signpost the reader.<br><br>4) Was PRISMA adhered to? If so, state this<br><br>5) 'Finally, as a sensitivity analysis, we included centre as a clustering variable for robust<br>standard errors' – this does not explain how the robust standard errors were calculated<br><br>6) 'Based on the confidence interval, the probability that the NOT strategy led to an increase in complications of 5% or less was

	90.7%.' – this is a strange statement. It is not the CI that gives the probability, but the sampling distribution. Moreover, assuming non-informative prior distributions, the authors must be assuming that the sampling distribution is approximately normal, and thus the posterior distribution for the risk difference is approximately normal? Or something of that ilk, in order to make probabilistic statements here (which I still agree are very useful).<br><br>7) 'The analysis including centre as a clustering variable for robust standard errors showed similar results and inference.' – please provide the results, at least in a supp material<br><br>8) 'Non-inferiority of the NOT strategy for simple appendicitis in children in terms of the<br>proportion of participants experiencing complications could not be demonstrated in this trial.' – is this really correct? It's too broad brush. Better to say that: Based on a non inferiority margin of 5%, there is some evidence (probability 91%) that the NOT group have a <= 5% increase in risk of complication, though this is not statistically significant (p-value 0.093) suggesting further research would still be welcome. This, to me, is a fairer conclusion that simply saying 'could not be demonstrated'.<br><br>9) The length of the discussion section should be reduced with more concise wording.<br><br>10) NNT should be provided with a 95% CI, and the limitations of this measure should be noted<br>https://discourse.datamethods.org/t/problems-with-nnt/195<br><br>11) Some tables show p-values for superiority, and others p-values for non-inferiority. Please be consistent. Some don't make sense: e.g. p-value for superiority is given for the outcome of length of stay, and the mean length of stay is higher for the NOT group, yet the p-value for 'superiority' is given as 0.001. Why is a longer stay 'superior'? Please check other outcomes too.<br><br>I look forward to the next revision and hope these comments are constructive.

VERSION 2 – AUTHOR RESPONSE

Reviewer 1:
Prof. Nigel Hall, University of Southampton

I have previously reviewed this manuscript and find that the majority of my comments have been addressed satisfactorily. I still think the discussion could be shortened significantly more without losing meaning but leave this to an editorial decision.

Thank you for reviewing our manuscript again and for your additional suggestions for improvement. We are pleased to note that our revisions to date have been largely satisfactory. We have aimed to further shorten the discussion as multiple reviewers have raised this issue.

See pages 17-21. Lines 536-696.

You may wish to comment on the fragility of your main outcome result. What would have happened if just one more patient in the appendicectomy arm had a complication making the risk difference zero?

> **In our discussion, we aimed to nuance our interpretation that conservative treatment for children with simple appendicitis is not non-inferior compared to appendicectomy in terms of complications, by indicating that approximately 91% of the confidence interval of the risk difference leans toward non-inferiority of conservative treatment. In all likelihood, conservative treatment would be considered non-inferior in practice, given the marginal differences observed in terms of complications in our primary outcome and secondary benefits. We have made no new changes in the manuscript.**

There are a few typos
- capital H line 45 on page 21, same x 2 in line 57 page 22, line 9 page 31, line 35 page 32
- page 30 line 38 'an' should be 'and'.

> **Thank you for pointing out these typos. We have corrected these in the manuscript.**

Reviewer 2:
Dr. Panu Mentula, University of Helsinki

This is my second time reviewing this manuscript. Most of the issues have been addressed adequately. Since author's suggest shared decision making, I'd like to see also negative findings of NOT strategy in the abstract to give more balanced view of the findings. These would include longer hospital stay, more extra visits and lower NPS score with NOT strategy. Healthcare costs are less relevant in shared decision making.

> **Thank you for reviewing our manuscript again and for your additional suggestions for improvement. We are pleased to note that our revisions to date have been largely satisfactory. The abstract has been revised to present a more balanced overview of both treatment strategies, supporting transparent shared decision making.**
>
> **See page 6. Lines 156-160 & 167-168.**

Reviewer 3:
Dr. Siv Fonnes, Herlev Hospital

The authors have revised the manuscript accordingly. I do still find that the current presentation of the tables with multiple columns reserved for missing data decreases the readability of the tables because less emphasis is on the actual data, and that it could be just as transparently reported using footnotes. This issue could be addressed in copy editing if needed.
I have no further comments and congratulate the authors on their conduct and reporting of the RCT.

> **We thank you for reviewing our manuscript again and for your additional suggestions for improvement. We are pleased to note that our revisions to data have been largely satisfactory. We understand your concerns regarding the readability of the tables. The column indicating missing data was included to ensure transparency for each outcome. We leave it to the discretion of the editorial board whether this information should remain in the table or be presented more appropriately as footnotes.**

Reviewer: 4

Prof. Richard Riley, University of Birmingham

Comments to the Author

I thank the authors for addressing my statistical comments from the Editorial meeting when they submitted to the BMJ. Generally these responses, and the changes, are appropriate. However, there remain a number of crucial suggestions and clarifications for the next revision, which I will look at, as follows:

1) I thank the authors for calculating the probability that the NOT group have an increase of complications of 5% or less. This is given as 90.7% in their response. However, in the paper, the authors should focus more on this, e.g. in the abstract, which currently says 'Non-inferiority of a NOT strategy for acute simple appendicitis in children could not be statistically demonstrated, but the majority of the 95%CI fell within the 5% noninferiority margin.' – it would be far stronger and clearer to also add the probability of falling <=5% here too.

> **Thank you - we have emphasized this more in the conclusion of the abstract.**
>
> **See page 6. Lines 163-164**

2) With this in mind, the authors have added that 'We used confidence distributions (based on the confidence interval of the difference in proportions between groups) to state the estimated probability that the NOT strategy led to an increase in complications of 5% or less" … what is unclear is what a confidence distribution is. No reference is given. I think (assume) that they take the sampling distribution (of the risk difference) as approximately normal and work out the proportion <=5%? If not, then how?

> **Our apologies for the lack of a reference and an additional explanation on our approach on this. We followed this paper by Prof. Ian Marscher (https://doi.org/10.1002/sim.10000). In essence, it is a frequentist approach to probabilistic statements such as requested in the previous round of revisions, without the need to specify priors. It indeed uses the estimated uncertainty or sampling distribution around the risk difference between the groups, taken to be a normal distribution. In a superiority design, this is useful for probabilistic statements on specific effect sizes as opposed to the simple null hypothesis. However, as this was a non-inferiority design, we already specified the non-inferiority margin of 5% to formalize the effect size for the alternative hypothesis. You will thus notice that the complement of the probabilistic statement coincides with the p-value for non-inferiority, which here seems to be the case by design. We have added the reference to the manuscript and hope that this is now sufficiently clear.**

3) 'A list of potential complications has been reported in the previously published study protocol' – this is so fundamental, that the list must be reported here too. Perhaps the list is already given when (some of) the outcomes are provided later in the methods text – if so, then state this to signpost the reader.

> **We have decided to provide a list with potential complications for clarity.**
>
> **See page 10. Lines 301-311.**

4) Was PRISMA adhered to? If so, state this

This study was a non-inferiority RCT such that we adhered to the CONSORT reporting guideline. We have added some text to the manuscript to clarify.

See page 8. Line 222.

5) 'Finally, as a sensitivity analysis, we included centre as a clustering variable for robust

standard errors' – this does not explain how the robust standard errors were calculated

Using a binomial model with identity link function and a sandwich variance estimator, with the cluster term being centre – now added to this sentence in the manuscript.

See page 14. Lines 431-432.

6) 'Based on the confidence interval, the probability that the NOT strategy led to an increase in complications of 5% or less was 90.7%.' – this is a strange statement. It is not the CI that gives the probability, but the sampling distribution. Moreover, assuming non-informative prior distributions, the authors must be assuming that the sampling distribution is approximately normal, and thus the posterior distribution for the risk difference is approximately normal? Or something of that ilk, in order to make probabilistic statements here (which I still agree are very useful).

We apologise for our confusing terminology. For more explanation, please see question 2. We have now revised 'based on the confidence interval' to 'based on the (normal) sampling distribution' in the manuscript.

See page 15. Line 472.

7) 'The analysis including centre as a clustering variable for robust standard errors showed similar results and inference.' – please provide the results, at least in a supp material

The point estimate for the risk difference was the same (0.7%) and the 95%CI was -6.1% to 7.4%. Thus, the upper limit increased to 7.4% (instead of 7.1% as reported in our main analysis). We have added this to the manuscript.

See page 15. Lines 475-478.

8) 'Non-inferiority of the NOT strategy for simple appendicitis in children in terms of the

proportion of participants experiencing complications could not be demonstrated in this trial.' – is this really correct? It's too broad brush. Better to say that: Based on a non inferiority margin of 5%, there is some evidence (probability 91%) that the NOT group have a <= 5% increase in risk of complication, though this is not statistically significant (p-value 0.093) suggesting further research would still be welcome. This, to me, is a fairer conclusion that simply saying 'could not be demonstrated'.

Thank you – We have made this adjustment for more nuance in our Discussion.

See page 17. Lines 540-542.

9) The length of the discussion section should be reduced with more concise wording.

We have aimed to shorten the Discussion, as this concern was raised by several reviewers in addition to yourself.

See pages 17-21. Lines 536-696.

10) NNT should be provided with a 95% CI, and the limitations of this measure should be noted https://discourse.datamethods.org/t/problems-with-nnt/195

Following Altman 1998, Confidence intervals for the number needed to treat | The BMJ (as the 95%CI for the risk difference includes zero, leading to opposite interpretations of the CI), the estimated NNTH was 151 (NNTB 17 to ∞ to NNTH 14). We have added this to the manuscript in Results and added your reference, too.

See page 15. Lines 469-472.

11) Some tables show p-values for superiority, and others p-values for non-inferiority. Please be consistent. Some don't make sense: e.g. p-value for superiority is given for the outcome of length of stay, and the mean length of stay is higher for the NOT group, yet the p-value for 'superiority' is given as 0.001. Why is a longer stay 'superior'? Please check other outcomes too.

We only use the term 'superiority' to distinguish the type of hypothesis testing from non-inferiority – yes, this can include both superiority or inferiority of the studied intervention. We have now revised this to 'superiority/inferiority'.

As the trial was powered on the primary endpoint complications with 1 year, a non-inferiority margin was only considered for the confirmatory test for the primary endpoint. Thus, other endpoints could not be tested with an alternative hypothesis for non-inferiority in mind, such that we tested it with the regular hypotheses i.e. superiority or inferiority. This is why the hypothesis testing changes. For clarity, we have separate tables for the primary endpoint (or its components, such as Clavien-Dindo) and the secondary endpoints to have a clear distinction between non-inferiority and superiority/inferiority.

See pages 28-29. Table 3a

Editor in Chief:

Dr. Sophie Cook

I was interested that in the discussion they don't make any reference to patient/parental preferences in terms of surgery vs conservative treatment when discussing risks and benefits. I imagine parents would find conservative treatment more acceptable/preferable for their child if the evidence were

equivocal, but the focus mostly on the surgical and anaesthetic aspects in their intro discussion.

Thank you for pointing out this important matter. We have aimed to shed more light and references to patient/parental preference in terms of surgery versus nonoperative treatment for simple appendicitis.

See page 19. Lines 618-624.

While I understand that they might find it harder to get a history from those younger than 7, their cut off seems a bit arbitrary to me. Some five year olds could be quite eloquent and able to voice their pain and symptoms, can they justify the choice of age cut off a bit more?

The rationale for selecting a minimum age cutoff of 7 years was twofold. First, based on a study conducted by our group (referenced/illustrated in the figure below) we observed that the proportion of complex appendicitis decreases in children aged 7 years and older compared with younger age groups. To enhance patient safety and to minimize the risk of inadvertently including children with complex appendicitis in the trial, this age threshold was therefore applied. Second, from an ethical and regulatory perspective, the establishment of a minimum age of 7 years was considered essential by our Medical Ethics Review Committee (METC), as it contributed to safeguarding participant safety within this trial. We do acknowledge that most comparable studies have applied a minimum age of 5 years; however, in our trial there was deliberately chosen for a more conservative threshold in light of the observed age-related risk profile and safety considerations.

No changes were made to the manuscript.

Figure 2. Age of included patients for simple and complex appendicitis. Cumulative number of patients with appendicitis over a time-span of 10 years: simple appendicitis N = 524, complex appendicitis N = 372.

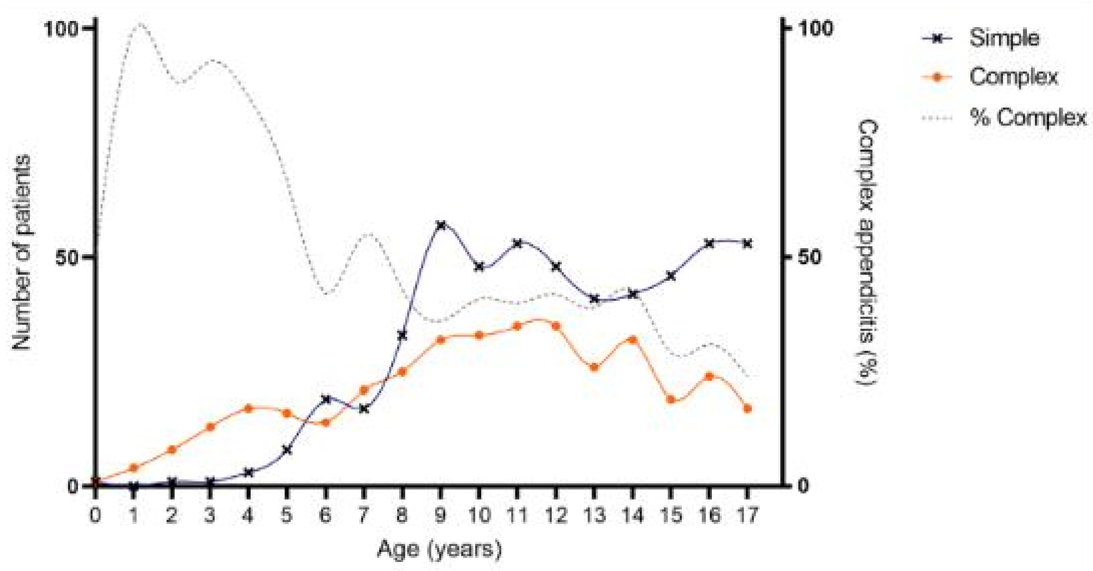

[Trends in Simple and Complex Appendicitis in Children and the Potential Correlation to

Common Viral Pathogens-A Retrospective Cohort Study between 2010 and 2019 in The Netherlands - PubMed

Likewise I think they need to justify the choice of non-inferiority design a bit more and also the potential pitfalls of their choice here.

The choice of a non-inferiority design was motivated by the objective to determine whether nonoperative treatment would not be 'worse' than surgical treatment with respect to complication rates, while offering several potential secondary benefits resulting from the avoidance of surgery. While a nonoperative treatment strategy inherently carries some risk, it avoids an invasive procedure in majority of children, thereby preventing surgery-related complications and postoperative morbidity, as well as reducing healthcare costs. Importantly, children who showed clinical deterioration or recurrent disease were and should be promptly treated with appendicectomy in the bigger framework. This would make nonoperative treatment a valid (first) treatment option for children with simple appendicitis.

From a methodological perspective, a non-inferiority trial is more complex than a conventional superiority design with respect to study design, sample size calculation, and statistical analysis. Nevertheless, all appropriate and necessary measures were taken to address these challenges in the APAC trial. A distinctive feature of non-inferiority designs is that, in addition to defining an acceptable non-inferiority margin, one must also make an explicit assumption regarding the expected difference between treatment groups. We had sound clinical grounds to hypothesize a lower complication rate in the non-operative group; however, we acknowledge that this assumption (like all assumptions inherent to trial design) may ultimately prove to be incorrect.

We have added some text to the Discussion section to address this.

See page 21. Lines 676-682.

VERSION 3 - REVIEW

**REVIEWER NAME**	Riley, Richard
**REVIEWER AFFILIATION**	University of Birmingham, Institute of Applied Health Research
**REVIEWER CONFLICT OF INTEREST**	None
**DATE REVIEW RETURNED**	09-Feb-2026

**GENERAL COMMENTS**	I thank the authors for their clear response and revision; I don't have any further comments
